# (CF_3_CO)_2_O/CF_3_SO_3_H-mediated synthesis of 1,3-diketones from carboxylic acids and aromatic ketones

**DOI:** 10.3762/bjoc.10.236

**Published:** 2014-09-26

**Authors:** JungKeun Kim, Elvira Shokova, Victor Tafeenko, Vladimir Kovalev

**Affiliations:** 1 Laboratory of Macrocyclic Receptors, Department of Chemistry, Moscow State University, Lenin’s Hills, Moscow 119991, Russia

**Keywords:** Claisen condensation, 1,3-diketones, heterocycles, triflic acid, trifluoroacetic acid anhydride

## Abstract

A very simple and convenient reaction for 1,3-diketone preparation from carboxylic acids and aromatic ketones in TFAA/TfOH system is described. When the β-phenylpropionic acids were used as starting materials, they initially gave 1-indanones and then underwent further acylation with the formation of 2-(β-phenylpropionyl)-1-indanones as the main reaction products. In addition, the application of the proposed protocol allowed for the synthesis of selected polysubstituted pyrazoles in a one-pot procedure directly from acids and ketones.

## Introduction

1,3-Diketones represent one of the most important class of organic compounds, since they are applied as key structural blocks in organic syntheses, exhibit different kinds of biological activities, and display a broad range of ionophoric properties [1–3]. The method most frequently used for 1,3-diketone synthesis is the Claisen condensation, which comprises the C-acylation of the α-position of ketones in the form of their metal enolates, enamines or silyl ethers, with or without a catalyst. To appear as an acylating agent one of the following compounds could be required: acyl halides and acid esters, including formates and oxalates, and acid anhydrides, dialkyl carbonates, methoxymagnesium methyl carbonate, *N*-acylimidazoles, acyl cyanides, and acylbenzotriazoles [4]. Though recently many modifications of this method have been proposed [5–11], it is noticeable that none of these techniques implements a direct synthesis of β-diketones from acids and ketones with an immediate activation of both carbonyl and methylene components in the course of the reaction.

Herein, we would like to describe a direct and operationally simple TFAA/TfOH-mediated synthesis of 1,3-diketones from unmodified carboxylic acids and ketones.

## Results and Discussion

Initially, an unusual transformation of the β-phenylpropionic acids was observed by us in a TFAA/TfOH/CH_2_Cl_2_ system (Table 1), which gave the impulse for this research. Surprisingly, it turned out that β-phenylpropionic acid (**1a**) in a TFAA/CH_2_Cl_2_ medium in the presence of TfOH (0.25 equiv, Table 1, entry 1) gave 2-(β-phenylpropionyl)-1-indanone (**3а**) in 51% yield as the major product, even though we expected 1-indanone (**2а**, <2%). When 0.5 equiv of TfOH was applied, **3а** and **2a** were obtained in 75 and 16% yield, respectively (Table 1, entry 2). Evidently, in this reaction, 1-indanone (**2а**), which was initially formed as the result of an intramolecular cyclization of **1a**, underwent a further acylation with the formation of 1,3-diketone **3a**. In contrast, γ-phenylbutanoic acid (**1c**) was quantitatively transformed only to the tetralone **2с** (Table 1, entries 10 and 11).

**Table 1:** TFAA/TfOH-mediated self-acylation of ω-phenylalkanoic acids **1a**–**c**^a^.

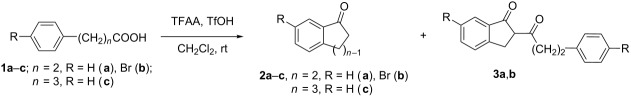					

Entry	**1**	TfOH (equiv)	Time (h)	Yield, %^b^	

				**2**	**3**

1	**1a**	0.25	2	**2a**, <2	**3a**, 55, (51)^c^
2	**1a**	0.5	2	**2a**, 19, (16)	**3a**, 79, (75)
3	**1a**	1.0	1	**2a**, 81	**3a**, 16
4	**1a**	1.5	0.5	**2a**, 96, (94)	**3a**, <1
5	**1b**	0.25	2	**2b**, 0	**3b**, 27
6	**1b**	0.5	2	**2b**, 0	**3b**, 67, (59)
7	**1b**	1.0	2	**2b**, 9, (8)	**3b**, 78, (70)
8	**1b**	1.5	2	**2b**, 28	**3b**, 58
9	**1b**	3.0	2	**2b**, 67, (62)	**3b**, <1
10	**1c**	0.25	1.5	**2с**, 98, (96)	
11	**1c**	0.5	1	**2с**, 96	

^a^ Reactions were performed by the addition of TfOH to a solution of **1a**–**c** (1.0 mmol) and TFAA (6 mmol) in 1.0 mL of dry CH_2_Cl_2_ at rt; ^b^yield determined by^1^H NMR; ^c^yield of individual product, purified by using silica gel column chromatography, is given in parentheses.

The acid-catalyzed cyclization of 3-arylpropanoic and 4-arylbutanoic acids to 1-indanones and 1-tetralones is well-known [12–18], but it appears that the further acylation and β-diketone formation has not been reported yet. While the use of acyl trifluoroacetates, generated in situ from a carboxylic acid and TFAA, for the aromatic acylation catalyzed by acid (H_3_PO_4_ [19–22] or TfOH [23–25]) has been reported, the 1,3-diketone formation has not been observed yet.

We concluded that in the work of reference [25], an apparently larger quantity of the super acidic TfOH was employed (4 equiv vs 0.25–1.5 equiv in our work), which possibly slowed down the reaction of ketone acylation. This is corroborated in the case of phenylpropionic acid **1а**. Here, the yield of the diketone **3a** decreased with an increase of the quantity of TfOH, whereas the yield of 1-indanone (**2a**) increased and reached 94% with 1.5 equiv of TfOH (Table 1, entry 4).

In the case of β-(4-bromophenyl)propionic acid (**1b**) with 0.5 equiv of TfOH, the reaction chemoselectively proceeds to give the only diketone **3b** (59%, Table 1, entry 6). The maximal yield of the diketone **3b** is obtained with 1 equiv of TfOH (70%, Table 1, entry 7), and a further increase of the TfOH quantity to 3 equiv results in the indanone **2b** as the only product (62%, Table 1, entry 9). Evidently, the intra- and intermolecular acylation of β-phenylpropionic acids in this reaction is dependent on the used TfOH quantity and the nature of the substituent in the phenyl moiety.

On the basis of the above results, we supposed that the acylation of ketones with carboxylic acids in a TFAA/TfOH/CH_2_Cl_2_ system could be applied as an effective method for the synthesis of β-diketone. It turned out that the acylation of different alkyl aryl ketones **2a–k** (indanones, tetralone, acetophenones, 2-acetylthiophene and methyl benzyl ketone) with alkanoic acids RCOOH **1d**–**h** (where R = 1-adamantylmethyl, neopentyl, isopropyl, methyl, phenyl) gave the corresponding β-diketones **3c–t** in 37–86% yields (Table 2).

**Table 2:** TFAA/TfOH-mediated acylation of aromatic ketones with alkanoic acids^а^.

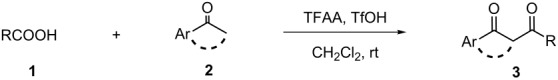					

Entry	Acid **1**, R		Ketone	1,3-Diketone	Yield,%

1	1-AdCH_2_	**1d**	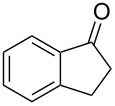 **2a**	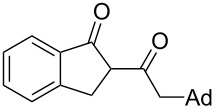 **3c**	79
2	*t*-BuCH_2_	**1e**	**2a**	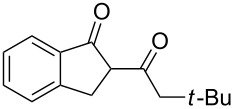 **3d**	74
3	iPr	**1f**	**2a**	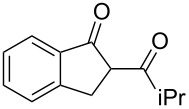 **3e**	50
4	Me	**1g**	**2a**	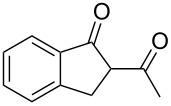 **3f**	77^b^
5	Ph	**1h**	**2a**	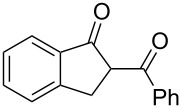 **3g**	66^c^
6	1-AdCH_2_	**1d**	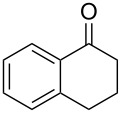 **2c**	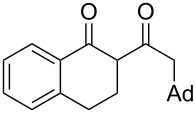 **3h**	(65)^d^
7	*t*-BuCH_2_	**1e**	**2c**	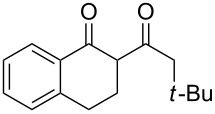 **3i**	57
8	Me	**1g**	**2c**	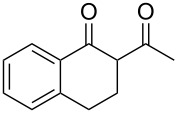 **3j**	53^b^
9	*t*-BuCH_2_	**1e**	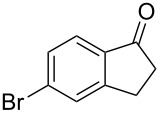 **2d**	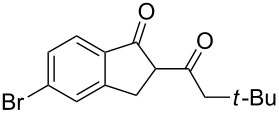 **3k**	86
10	1-AdCH_2_	**1d**	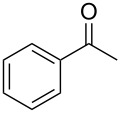 **2e**	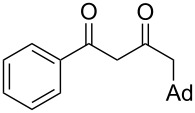 **3l**	47
11	*t*-BuCH_2_	**1e**	**2e**	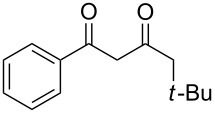 **3m**	69^b^
12	*t*-BuCH_2_	**1e**	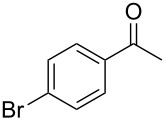 **2f**	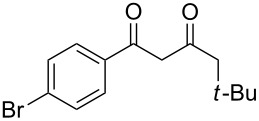 **3n**	61
13	Me	**1g**	**2f**	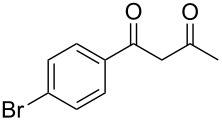 **3o**	41^b,c^
14	1-AdCH_2_	**1d**	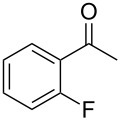 **2g**	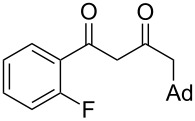 **3p**	48
15	1-AdCH_2_	**1d**	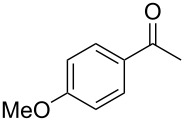 **2h**	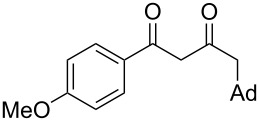 **3q**	37
16	1-AdCH_2_	**1d**	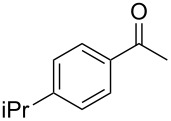 **2i**	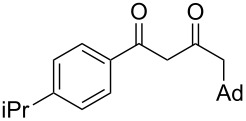 **3r**	49
17	1-AdCH_2_	**1d**	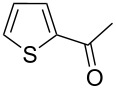 **2j**	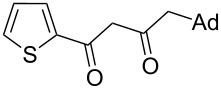 **3s**	43
18	1-AdCH_2_	**1d**	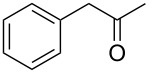 **2k**	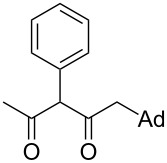 **3t**	64

^a^Reaction conditions: ketone (1 mmol), acid (1 mmol), TFAA (6 mmol), TfOH (0.5 mmol) in 1 mL CH_2_Cl_2_, 2–4 h, rt; ^b^2 mmol of acid was used; ^c^1.5 mmol of TfOH was used; ^d^yield determined by ^1^H NMR.

In most cases reactions were carried out at the molar ratios of acid:ketone:TFAA:TfOH = 1:1:6:0.5. For the reaction of 1-indanone (**2а**) and 1-adamantylacetic acid (**1d**) it was shown that in the absence of TfOH the yield of diketone **3c** significantly dropped (<2%). With 0.25–1.5 equiv of TfOH the yield of the diketone **3c** reached its maximal value (~80%) and decreased with a greater excess (3 equiv) of TfOH (57%). Apparently, the reduction of the TFAA excess (from 6 to 3 equiv) slightly lowered the yield of the diketone **3c** to 68%, although the quantity of TFAA was not optimized for these reactions. An excess of the acid **1e** (2 equiv vs 1 equiv) in the acylation of acetophenone (**2e**) only modestly increase the yield of diketone **3d** from 64 to 69% (Table 2, entry 11), and usually we added equimolecular quantities of an acid and a ketone. An excess of acetic acid in acylation reactions (Table 2, entries 4, 8 and 13) was employed considering that this acid was partially self-acylated under the reaction conditions. A probable mechanism of the reaction of ketone acylation by acids in TFAA/TfOH media may be that after the acyl trifluoroacetates are generated in situ, triflic acid becomes involved in the enolization of ketones and increases the acylating ability of acyl trifluoroacetates. However, with an increase of the TfOH quantity (>1.5 equiv) a retardation of the ketone acylation is observed, presumably as a result of its protonation.

**Scheme 1:**
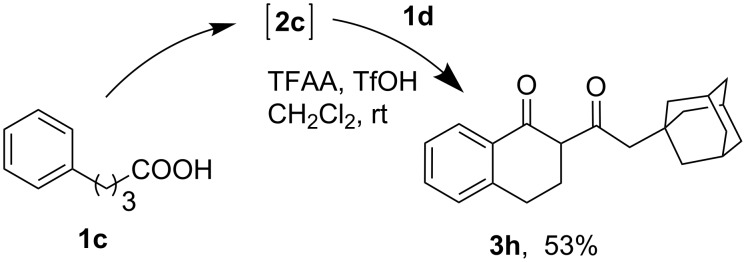
One-pot synthesis of diketone **3h** from acids **1d** and **1c**.

Since the γ-phenylbutanoic acid (**1c**) was quantitatively converted to 1-tetralone (**2c**) under the acylation conditions, the diketone **3h** (Table 2, entry 6) was also obtained by a two-stage one-pot approach, i.e., the intramolecular cyclization of the acid **1c** (to form the tetralone **2c**) and the following acylation with 1-adamantylacetic acid (**1d**) (Scheme 1).

The acylation of the methyl benzyl ketone (**2k**) by 1-adamantylacetic acid (**1d**) proceeded with regioselectivity at the α-СН_2_-group and gave the diketone **3t** in good yield (Table 2, entry 18), which is apparently associated with the enolization ability of **2k** in the benzyl fragment of the molecule.

**Scheme 2:**
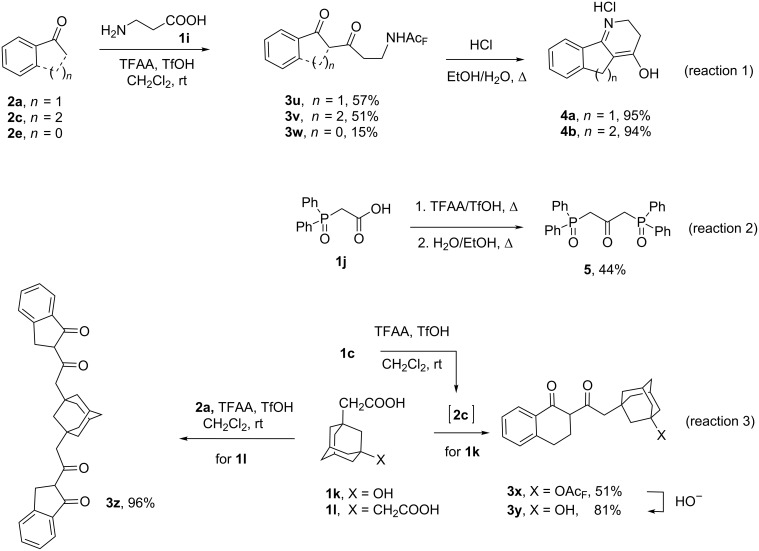
Scope and limitations.

To enhance the application of the considered reaction, we investigated the acylation of the ketones by several functionally substituted carboxylic acids. Whereas glycine did not react with the ketones, β-alanine (**1i**) reacting with 1-indanone, 1-tetralone and acetophenone formed the corresponding trifluoroacylated β-aminodiketones **3u–w** (reaction 1 in Scheme 2). A fuller acylation for **2a** and **2c** was achieved when 1.5 equiv of the acid **1i** and 1 equiv of TfOH were used, whereas the yield of diketone **3w** still remained low under these conditions. The hydrolysis of trifluoroacetates **3u**, **3v** under reflux in dilute HCl was followed by the intramolecular cyclization and led to the unknown heterocycles **4а**,**b**.

**Figure 1:**
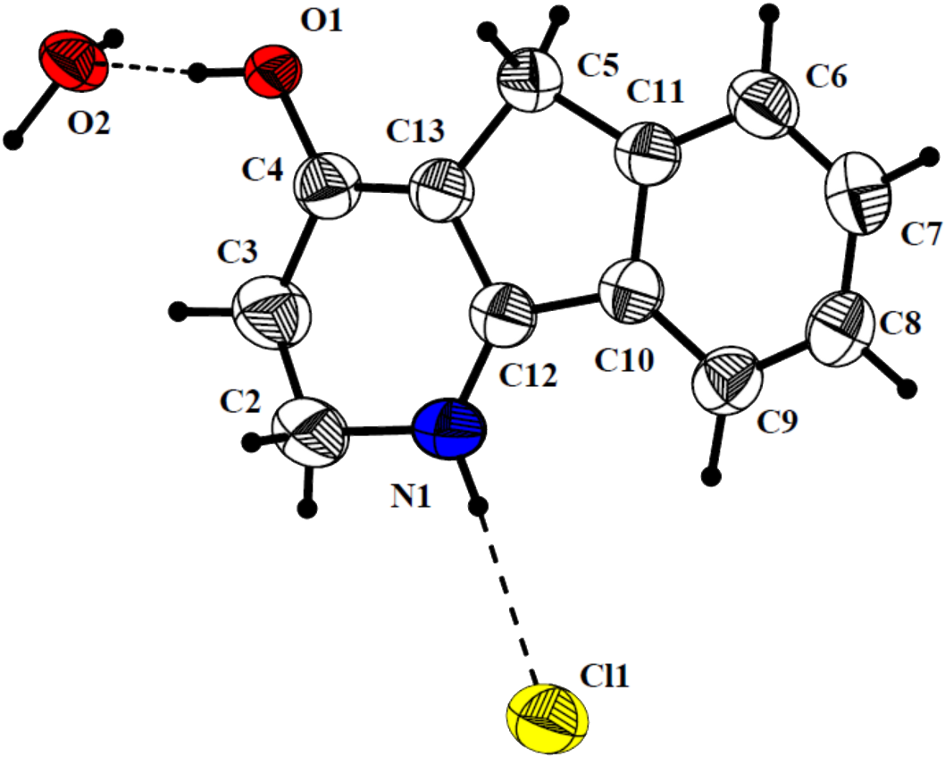
The molecular structure of **4a**.

Unambiguous evidence for the structure of heterocycle **4a** was obtained by X-ray diffraction analysis [26]. The crystal structure of **4a** is mediated by hydrogen bonds with a cation, a chloride anion and a solvating water molecule as participants (Figure 1).

By acylation of 1-indanone (**2a**) with (diphenylphosphoryl)acetic acid (**1j**), the self-acylation of the acid **1j** already occurred at room temperature, which complicated the separation of the desired dicarbonyl compound. When the acid **1j** was heated under the conditions of TFAA/TfOH-mediated self-acylation of alkanoic acids recently reported by us [27] and decarboxylation was subsequently carried out, 1,3-diphenylphosphorylated acetone **5** could be obtained (reaction 2 in Scheme 2).

The two-stage one-pot reaction of γ-phenylbutanoic acid (**1c**) and 3-hydroxy-1-adamantylacetic acid (**1k**) gave as a result the trifluoroacetylated hydroxydiketone **3x**, which could be hydrolyzed to the corresponding alcohol **3y** (reaction 3 in Scheme 2). The acylation of 1-indanone (**2а**) with 2,2’-(adamantane-1,3-diyl)diacetic acid (**1l**) gave the tetraketone **3z**.

**Scheme 3:**
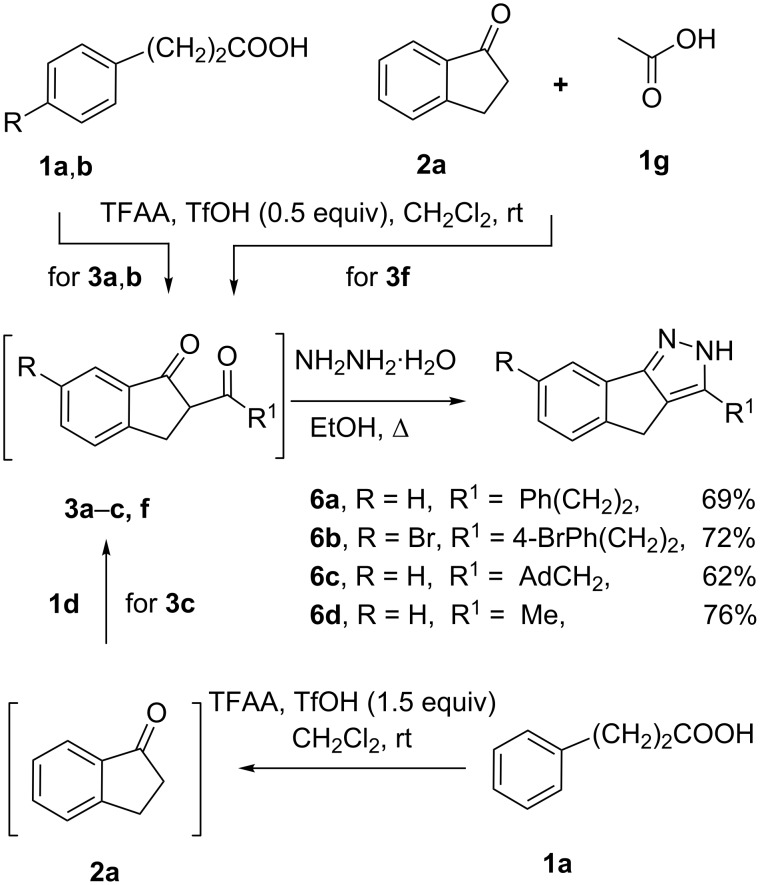
One-pot synthesis of pyrazoles **6**.

Finally, we used our method in a two-stage one-pot syntheses of pyrazoles, which find extensive use in the pharmaceutical industry [8,28]. Diketones **3a**–**c**,**f**, essential for synthesis of the pyrazoles **6a**–**d**, were obtained by one of the three routes (Scheme 3). The one-pot intra- and intermolecular acylation of β-phenylpropionic acids **1а**,**b** gave **3а**,**b**. The acylation of the 1-indanone (**2а**) by acetic acid (**1g**) yielded **3f**. The selective intramolecular cyclization of acid **1а** was used to obtain the intermediate ketone **2а**, which was further acylated by the acid **1d** to finally give **3c**. Upon the formation of the diketones, the reaction mixtures were evaporated in vacuum, the residues were dissolved in ethanol, and the following reaction with hydrazine hydrate gave the pyrazoles **6a**–**d** in 62–76% yield.

The products were identified by ^1^H and ^13^C NMR spectroscopy, microanalysis and by spectral comparison with the known compounds. The molecular structures of compounds **3с**, **3u**, **4a**, **6b** were confirmed by X-ray diffraction [26]. The obtained diketones were mainly present in the enol form in CDCl_3_ solution. The enol structures of the β-diketones **3** were supported by ^1^H/^13^C NMR spectra, which showed a set of singlets at δ 5.9–6.2 corresponding to the α-olefinic protons for **3l**–**3r**, **3s** and **3w**, signals at δ 106.5–116.1 and at δ 96.1–102.7 were assigned to the α-olefinic carbon for **3a–3k**, **3u**, **3v**, **3x–3z** and **3l–3r**, **3s**, **3w**, respectively. The minor keto tautomers (2–30%) were characterized by ^1^H multiplets or singlets between δ 3.6 and 4.2 (4.70 for **3t**) and ^13^C NMR signals between δ 59.0 and 61.5.

## Conclusion

In summary, we proposed a simple and effective synthetic approach to 1,3-diketones based on the TFAA/TfOH-mediated acylation of ketones with carboxylic acids. Advantages of this approach are the ready availability of the starting materials, the simple operational procedure, and the possibility to realize the one-pot immediate syntheses of 1,3-diketones and pyrazoles directly from acids and ketones. Moreover, 1,3-diketones and pyrazoles can be obtained from unmodified β-phenylpropionic acids. Given the importance of 1,3-dicarbonyl compounds in general, we expect that this reaction has a wide application in the realm of synthetic chemistry.

## Experimental

**General procedure for the synthesis of diketones**: A solution of carboxylic acid (1 mmol), ketone (1 mmol, if required) and TFAA (0.85 mL, 6 mmol) in dichloromethane (1 mL) was stirred for 15 min at rt. The required quantity of triflic acid (usually 44 μL, 0.5 mmol) was then added, and the resulting solution was stirred at rt for 1–4 h (24 h for **3v** and **3w**) under the conditions indicated in Scheme 1, Scheme 2, Table 1, and Table 2 (TLC monitoring). The reaction mixture was evaporated under reduced pressure, and after quenching with water, the residue was redissolved in dichloromethane (10 mL), washed with 5% NaHCO_3_ (2 × 3 mL), water (2 × 3 mL), and dried over MgSO_4_. The solvent was removed in vacuum, and the crude reaction mixture was purified by silica gel chromatography (*n*-hexane/CH_2_Cl_2_/MeOH).

As illustrative examples, compounds **3a** and **3c** are prepared as follows.

**2-(3-Phenylpropionyl)-1-indanone (3a):** Obtained from β-phenylpropionic acid (**1a**, 150 mg, 1 mmol), TFAA (0.85 mL, 6 mmol) and TfOH (44 μL, 0.5 mmol) in 75% (100 mg) yield as a red solid. Mp 65–66 °C (Lit. [29]: mp 68–69 °C); ^1^H NMR (400 MHz, CDCl_3_) keto-enol (20:80); enol tautomer: δ 7.80 (d, *J* = 7.6 MHz, H^Ar^, 1H), 7.55–7.33 (m, H^Ar^, 3H), 7.32–7.15 (m, H^Ar^, 5H), 3.40 (s, CH_2_^Ind^, 2H), 3.04 (t, *J* = 7.7 Hz, CH_2_, 2H), 2.73 (t, *J* = 7.7 Hz, CH_2_, 2H); ^13^С NMR (100 MHz, CDCl_3_) δ 191.1 (CO), 179.7 (C=*C*(OH)), 147.4 (C^Ar^), 140.6 (C^Ar^), 138.1 (C^Ar^), 132.7 (C^Ar^), 128.5 (CH^Ar^), 128.3 (CH^Ar^), 127.2 (CH^Ar^), 126.3 (CH^Ar^), 125.6 (CH^Ar^), 123.0 (CH^Ar^), 110.5 (*C*=C(OH)), 36.8 (CH_2_), 31.6 (CH_2_), 29.9 (CH_2_).

**2-[2-(1-Adamantyl)acetyl]-1-indanone (3c):** Obtained from 1-adamantylacetic acid (**1d**, 194 mg, 1 mmol), 1-indanone (**2a**, 132 mg, 1 mmol), TFAA (0.85 mL, 6 mmol) and TfOH (44 μL, 0.5 mmol) in 79% (240 mg) yield as a red solid. Mp 154 °C; ^1^H NMR (400 MHz, CDCl_3_) keto-enol (2:98), enol tautomer: δ 7.81 (d, *J* = 7.6 Hz, H^Ar^, 1H), 7.52 (t, *J* = 7.4 Hz, H^Ar^, 1H), 7.46 (d, *J* = 7.5 Hz, H^Ar^, 1H), 7.38 (t, *J* = 7.4 Hz, H^Ar^, 1H), 3.58 (s, CH_2_^Ind^, 2H), 2.18 (s, CH_2_Ad, 2H), 1.98 (bs, CH^Ad^, 3H,), 1.75–1.59 (m, CH_2_^Ad^, 12H); ^13^С NMR (100 MHz, CDCl_3_) δ 193.6 (CO), 177.1 (C=*C*(OH)), 148.0 (C^Ar^), 138.5 (C^Ar^), 132.9 (CH^Ar^), 127.2 (CH^Ar^), 125.6 (CH^Ar^), 123.2 (CH^Ar^), 111.7 (*C*=C(OH)), 48.6 (CH_2_Ad), 43.0 (CH_2_^Ad^), 36.7 (CH_2_^Ad^), 35.1 (C^Ad^), 30.7 (CH_2_), 28.7 (CH^Ad^); Anal. calcd for C_21_H_24_O_2_: C, 81.78; H, 7.84; found: C, 82.23; H 7.67.

**Typical procedure for the synthesis of heterocycles 4a,b:** A solution of diketone **3u** or **3v** (1 mmol) in an ethanol (20 mL)/water (4 mL)/HCl_conc_ (4 mL) mixture was heated under reflux for 6 h. After the reaction was completed (TLC control) the solvent was evaporated, the product was washed with diethyl ether and hexane and dried.

**1-Hydroxy-4-aza-2,3-dihydrofluorene hydrochloride (4a):** Obtained from diketone **3u** (299 mg, 1 mmol) in 95% (210 mg) yield as a brown solid. Mp 110–112 °С; ^1^H NMR (400 MHz, methanol-*d*_4_) δ 7.97 (d, *J* = 7.8 Hz, H^Ar^, 1H), 7.69 (t, *J* = 7.5 Hz, H^Ar^, 1H), 7.59 (d, *J* = 7.7 Hz, H^Ar^, 1H), 7.56 (t, *J* = 7.5 Hz, H^Ar^, 1H), 3.99 (t, *J* = 8.5 Hz, CH_2_, 2H), 3.79 (s, CH_2_, 2H), 2.91 (t, *J* = 8.5 Hz, CH_2_, 2H); ^13^С NMR (100 MHz, methanol*-d*_4_) δ 173.7 (C), 173.2 (C), 149.5 (C), 135.1 (CH), 132.0 (C), 127.4 (CH), 126.1 (CH), 123.8 (CH), 105.9 (C), 41.1 (CH_2_), 30.7 (CH_2_), 27.3 (CH_2_); Anal. calcd for C_12_H_11_NO·HCl: C, 65.02; H, 5.46; N, 6.32; found: C, 64.72; H, 5.61; N, 6.24.

**1,3-Bis(diphenylphosphoryl)acetone (5):** A solution of diphenylphosphorylacetic acid (260 mg, 1 mmol) in a mixture of TFAA (1.95 mL, 13.8 mmol) and TfOH (0.05 mL, 0.57 mmol) was kept at 60–65 °C for 1.5 h. The solvent was evaporated under reduced pressure, the obtained residue was stirred in a water (4 mL)/EtOH (4 mL) solution under reflux for 2 h, cooled and concentrated. The residue was adjusted to pH 7.5 with 1 N NaHCO_3_, the solid formed was filtered, washed with water, and dried. The product was purified by column chromatography (eluent: CH_2_Cl_2_/MeOH 50:1). Yield: 44% (101 mg), white solid; mp 173–175 °С (Lit. [30]: mp 175–176 °C); ^1^H NMR (400 MHz, CDCl_3_) δ 7.80–7.68 (m, H^Ar^, 8H), 7.57–7.41 (m, H^Ar^, 12H), 3.97 (d, *J* = 14.3 Hz, CH_2_, 4H); ^13^С NMR (100 MHz, CDCl_3_) δ 195.6 (CO), 132.2 (CH^Ar^), 131.2 (C^Ar^), 130.9 (d, *J* = 10.6 Hz, CH^Ar^,), 128.7 (CH^Ar^, *J* = 12.7 Hz), 48.2 (d, *J* = 55.7, CH_2_, Hz); ^31^P NMR (CDCl_3_) δ 27.1.

**The synthesis of pyrazoles 6**. As an illustrative example, compound **6a** is prepared as follows. A solution of β-phenylpropionic acid (**1a**, 150 mg, 1 mmol) and TFAA (0.85 mL, 6 mmol) in 1 mL CH_2_Cl_2_ was stirred for 15 min at rt. Then TfOH (44 μL, 0.5 mmol) was added, and the reaction mixture was kept for 2 h. On completion of the reaction, the solvent was removed under reduced pressure. The crude **3a** was dissolved in 5 mL ethanol and heated under reflux with hydrazine hydrate (0.1 mL, 2 mmol). After 2 h the solvent was evaporated, and the remaining oil was dissolved in CH_2_Cl_2_, washed with 5% NaHCO_3_, water, and dried over MgSO_4_. The product was purified by means of column chromatography (SiO_2_ 60, eluent: CH_2_Cl_2_/MeOH 50:1). Yield: 69% (90 mg), brown solid; mp 108–110 °С; ^1^H NMR (400 MHz, CDCl_3_) δ 7.71 (d, *J* = 7.2 Hz, H^Ar^, 1H), 7.44 (d, *J* = 7.2 Hz, H^Ar^, 1H), 7.40–7.17 (m, H^Ar^, 4H), 7.14 (d, *J* = 6.8 Hz, H^Ar^, 2H), 3.42 (s, CH_2_, 2H), 3.04 (m, CH_2_, 4H); ^13^С NMR (100 MHz, CDCl_3_) δ 148.7 (C), 140.8 (C), 137.9 (C), 134.7 (C), 128.5 (CH), 128.4 (CH), 126.4 (2CH), 126.3 (CH), 125.8 (CH), 122.7 (CH), 121.6 (C), 119.9 (CH), 34.8 (CH_2_), 28.4 (CH_2_), 27.5 (CH_2_); Anal. calcd for C_18_H_16_N_2_: C, 83.05; H, 6.19; N, 10.76; found: C, 82.67; H, 6.35; N, 10.37.

## Supporting Information

File 1 Detailed experimental procedures and characterization of compounds **3c–z**, **4b** and **6b–d**, figures of the molecular structures of compounds **3c**, **3u**, **4a** and **6b**, and copies of ^1^H, ^13^C and ^31^P NMR spectra for all new compounds.

File 2 Crystallographic information for compound **3c**.

File 3 Crystallographic information for compound **3u**.

File 4 Crystallographic information for compound **4a**.

File 5 Crystallographic information for compound **6b**.
