# Supplementary material for: (CF3CO)2O/CF3SO3H-mediated synthesis of 1,3-diketones from carboxylic acids and aromatic ketones
Source: Beilstein J Org Chem. 2014 Sep 26;10:2270–8. doi: 10.3762/bjoc.10.236 (PMC4187061; doi:10.3762/bjoc.10.236)
Supplement: File 1 — Detailed experimental procedures and characterization of compounds 3c–z, 4b and 6b–d, figures of the molecular structures of compounds 3c, 3u, 4a and 6b, and copies of 1H, 13C and 31P NMR spectra for all new compounds. [file Beilstein_J_Org_Chem-10-2270-s001.pdf]

Supporting information

for

$(CF_3CO)_2O/CF_3SO_3H$-mediated synthesis of 1,3-diketones from carboxylic acids and aromatic ketones

JungKeun Kim, Elvira Shokova, Victor Tafeenko and Vladimir Kovalev*

Address: Laboratory of Macrocyclic Receptors, Department of Chemistry, Moscow State University, Lenin's Hills, Moscow 119991, Russia

Email: Vladimir Kovalev* - kovalev@petrol.chem.msu.ru

* Corresponding author

Detailed experimental procedures and characterization of compounds **3c–z**, **4b**, and **6b–d**, figures of the molecular structures of compounds **3c**, **3u**, **4a**, **6b** and copies of ^{1}H, ^{13}C and ^{31}P NMR spectra for the new compounds

Contents:

1. General information. S2

2. Synthetic procedures and characterization data. S2

3. Molecular structures of compounds **3c**, **3u**, **4a**, and **6b** (Fig. **S1-S4**). S14

4. Copies of ^{1}H, ^{13}C and ^{31}P spectra (Fig. **S5-S69**). S16

1. General information.

^1H, ^{13}C and ^{31}P NMR spectra were measured on a Bruker Avance 400 spectrometer with solvent signals as internal reference (85% H_3PO_4 as external standard for ^{31}P NMR). To assign the chemical shifts in the ^1H and ^{13}C NMR spectra the following symbols were used: H^R and C^R for hydrogen and carbon atoms of the aryl (R = Ar), adamantyl (R = Ad) and indanone (R = Ind) fragments. Melting points are uncorrected. X-ray measurements were performed with an Enraf-Nonius CAD-4 diffractometer. Reactions were monitored by thin-layer chromatography (TLC) carried out on aluminum sheets silica gel 60 F_{254} (Merck), and the products were visualized by UV detection. Column chromatography was performed on silica gel (Merck Kieselgel 60). Chemicals were commercial grade and used without further purification. Solvents were purified and dried according to standard procedures. TFAA was freshly distilled from P_2O_5.

2. Synthetic procedures and characterization data.

General procedure for the synthesis of diketones: A solution of carboxylic acid (1 mmol), ketone (1 mmol, if required) and TFAA (0.85 mL, 6 mmol) in dichloromethane (1 mL) was stirred for 15 min at rt. The required quantity of triflic acid (usually 44 μL, 0.5 mmol) was then added, and the resulting solution was stirred at room temperature for 1-4 h (24 h for **3v** and **3w**) in conditions indicated in Schemes 1, 2 and Tables 1, 2 (TLC monitoring). Reaction mixture was evaporated under reduced pressure, and after quenching with water, the residue was redissolved in dichloromethane (10 mL), washed with 5% $NaHCO_3$ (2x3 mL), water (2x3 mL), and dried over $MgSO_4$. Solvent was removed in vacuum, and the crude reaction mixture was purified by silica gel chromatography (*n*-hexane/CH_2Cl_2/MeOH).

6-Bromo-2-[3-(4-bromophenyl)propionyl]-1-indanone (3b): Obtained from β-(4-bromophenyl)propionic acid **1b** (229 mg, 1 mmol), TFAA (0.85 mL, 6 mmol) and TfOH (88 μL, 1 mmol). Yield 70% (148 mg), red solid, mp 147-148 °C. $C_{18}H_{14}Br_2O_2$ (422.12): calcd. C

51.22, H 3.34; found C 51.01, H 3.23. ^{1}H NMR (400 MHz, $CDCl_3$): keto-enol (15:85%), enol tautomer, δ = 7.91 (s, 1H, H^{Ar}), 7.63 (d, *J* = 7.5 Hz, 1H, H^{Ar}), 7.40 (d, *J* = 7.9 Hz, 2H, H^{Ar}), 7.31 (d, *J* = 7.8 Hz, 1H, H^{Ar}), 7.10 (d, *J* = 7.9 Hz, 2H, H^{Ar}), 3.36 (s, 2H, CH_2^{Ind}), 2.99 (t, *J* = 7.4 Hz, 2H, CH_2), 2.72 (t, *J* = 7.4 Hz, 2H, CH_2). ^{13}C NMR (100 MHz, $CDCl_3$): δ 187.9 (CO), 180.7 (C=<u>C</u>(OH)), 145.2 (C^{Ar}), 139.5 (C^{Ar}), 139.1 (C^{Ar}), 135.0 (CH^{Ar}), 131.2 (CH^{Ar}), 129.7 (CH^{Ar}), 126.7 (CH^{Ar}), 125.6 (CH^{Ar}), 121.1 (C^{Ar}), 119.8 (C^{Ar}), 110.5 (<u>C</u>=C(OH)), 36. 5 (CH_2), 30.4 (CH_2), 29.5 (CH_2).

2-(3,3-Dimethylbutyryl)-1-indanone (3d): Obtained from 3,3-dimethylbutyric acid **1e** (0.116 mg, 1 mmol), 1-indanone **2a** (132 mg, 1 mmol), TFAA (0.85 mL, 6 mmol) and TfOH (44 μL, 0.5 mmol). Yield 74% (170 mg), red solid, mp 91-93 °C. $C_{15}H_{18}O_2$ (230.31): calcd. C 78.23, H 7.88; found C 78.54, H 7.71. ^{1}H NMR (400 MHz, $CDCl_3$): keto-enol (7:93%), enol tautomer, δ 7.84 (d, *J* = 7.7 Hz, 1H, H^{Ar}), 7.56 (t, *J* = 7.4 Hz, 1H, H^{Ar}), 7.50 (d, *J* = 7.5 Hz, 1H, H^{Ar}), 7.42 (t, *J* = 7.4 Hz, 1H, H^{Ar}), 3.62 (s, 2H, CH_2^{Ind}), 2.31 (s, 2H, *t*-BuCH_2), 1.10 (s, 9H, $C(CH_3)_3$). ^{13}C NMR (100 MHz, $CDCl_3$): δ = 193.4 (CO), 177.4 (C=<u>C</u>(OH)), 147.6 (C^{Ar}), 138.1 (C^{Ar}), 132.5 (CH^{Ar}), 126.9 (CH^{Ar}), 125.3 (CH^{Ar}), 122.9 (CH^{Ar}), 111.1 (<u>C</u>=C(OH)), 47.1 (*t*-BuCH_2), 32.5 (<u>C</u>(CH_3)$_3$), 30.2 (CH_2), 29.8 (C(<u>C</u>H_3)).

2-Isobutyryl-1-indanone (3e): Obtained from *iso*-butyric acid **1f** (88 mg, 1 mmol), 1-indanone **2a** (132 mg, 1 mmol), TFAA (0.85 mL, 6 mmol) and TfOH (44 μL, 0.5 mmol). Yield 50% (101 mg), yellow oil. $C_{13}H_{14}O_2$ (202.26): calcd. C 77.20, H 6.98; found C 77.01, H 6.80. ^{1}H NMR (400 MHz, $CDCl_3$): keto-enol (15:85%), enol tautomer, δ 7.79 (d, *J* = 7.4 Hz, 1H, H^{Ar}), 7.62-7.31 (m, 3H, H^{Ar}), 3.58 (c, 2H, CH_2^{Ind}), 2.73 (m, 1H, C<u>H</u>(CH_3)$_2$), 1.24 (d, *J* = 6.7 Hz, 6H, CH(C<u>H</u>$_3$)$_2$). ^{13}C NMR (100 MHz, $CDCl_3$): δ 191.2 (CO), 185.8 (C=<u>C</u>(OH)), 147.2 (C^{Ar}), 138.2 (C^{Ar}), 132.4 (CH^{Ar}), 127.1 (CH^{Ar}), 125.6 (CH^{Ar}), 122.8 (CH^{Ar}), 108.5 (<u>C</u>=C(OH)), 33.5 (CH_2), 29.9 (C<u>H</u>(CH_3)$_2$), 18.9 (CH(C<u>H</u>$_3$)$_2$).

2-Acetyl-1-indanone (3f): Obtained from acetic acid **1g** (0.12 mL, 2.0 mmol), 1-indanone **2a** (132 mg, 1 mmol), TFAA (0.85 mL, 6 mmol) and TfOH (44 μL, 0.5 mmol). Yield 77% (134

mg), red solid, mp 74-75 °C (Lit. [1] 75-76 °C). ^{1}H NMR (400 MHz, $CDCl_3$): keto-enol (15:85%), enol tautomer, δ 7.78 (d, *J* = 7.7 Hz, 1H, H^{Ar}), 7.51 (t, *J* = 7.4 Hz, 1H, H^{Ar}), 7.48-7.42 (m, 1H, H^{Ar}), 7.41-7.31 (m, 1H, H^{Ar}), 3.51 (s, 2H, CH_2^{Ind}), 2.13 (s, 3H, CH_3). ^{13}C NMR (100 MHz, $CDCl_3$): δ 190.9 (CO), 177.1 (C=C(OH)), 147.1 (C^{Ar}), 137.7 (C^{Ar}), 132.3 (CH^{Ar}), 126.8 (CH^{Ar}), 125.3 (CH^{Ar}), 122.6 (CH^{Ar}), 110.0 ($\underline{C}$=C(OH)), 29.8 (CH_2), 20.6 (CH_3).

2-Benzoyl-1-indanone (3g): Obtained from benzoic acid **1h** (120 mg, 1.0 mmol), 1-indanone **2a** (132 mg, 1 mmol), TFAA (0.85 mL, 6 mmol) and TfOH (133 µL, 1.5 mmol). Yield 66% (160 mg), yellow solid, mp 92-94 °C (Lit. [2] 94-96 °C). ^{1}H NMR (400 MHz, $CDCl_3$): keto-enol (7:93%), enol tautomer, δ 15.03 (br s, 1H), 8.00-7.92 (m, 2H, H^{Ar}), 7.89 (d, *J* = 7.6 Hz, 1H, H^{Ar}), 7.65-7.47 (m, 5H), 7.43 (t, *J* = 7.3 Hz, 1H, H^{Ar}), 3.91 (s, 2H,); ^{13}C NMR (100 MHz, $CDCl_3$): δ = 195.7 (CO), 170.7 (CO), 148.5 (C^{Ar}), 137.8 (C^{Ar}), 134.8 (C^{Ar}), 133.3 (CH^{Ar}), 131.2 (CH^{Ar}), 128.5 (CH^{Ar}), 128.1 (CH^{Ar}), 127.4 (CH^{Ar}), 125.5 (CH^{Ar}), 123.4 (CH^{Ar}), 109.4 ($\underline{C}$=C(OH)), 32.1 (CH_2).

2-[2-(1-Adamantyl)acetyl]-1-tetralone (3h): A solution of 4-phenylbutyric acid **1c** (164 mg, 1 mmol) in TFAA (0.85 mL, 6 mmol) and CH_2Cl_2 (1 mL) was stirred for 15 min at rt and then TfOH (0.44 µL, 0.5 mmol) was added. The reaction mixture was kept for 1 h (1-tetralone **2c** synthesis) and then 1-adamantylacetic acid **1d** (194 mg, 1 mmol) was added. Further manipulations were as described in the typical procedure. Yield 53% (171 mg), brown solid, mp 100-101 °C. $C_{22}H_{26}O_2$ (322.45): calcd. C 81.95, H 8.13; found C 82.19, H 8.02. ^{1}H NMR (400 MHz, $CDCl_3$): keto-enol (4:96%), enol tautomer, δ 7.98 (d, *J* = 7.7 Hz, 1H, H^{Ar}), 7.39 (t, *J* = 7.5 Hz, 1H, H^{Ar}), 7.31 (t, *J* = 7.5 Hz, 1H, H^{Ar}), 7.18 (d, *J* = 7.4 Hz, 1H, H^{Ar}), 2.83 (t, *J* = 7.0 Hz, 2H, CH_2), 2.65 (t, *J* = 7.0 Hz, 2H, CH_2), 2.26 (s, 2H, CH_2Ad), 1.96 (bs, 3H, CH^{Ad}), 1.74-1.59 (m, 12H, CH_2^{Ad}). ^{13}C NMR (100 MHz, $CDCl_3$): δ = 189.9 (CO), 182.3 (C=$\underline{C}$(OH)), 141.5 (C^{Ar}), 132.1 (C^{Ar}), 132.1 (CH^{Ar}), 127.5 (CH^{Ar}), 126.8 (CH^{Ar}), 126.3 (CH^{Ar}), 107.5 ($\underline{C}$=C(OH)), 48.2 (CH_2Ad), 42.9 (CH_2^{Ad}), 36.7 (CH_2^{Ad}), 35.7 (C^{Ad}), 28.8 (CH^{Ad}), 28.6 (CH_2), 24.0 (CH_2).

2-(3,3-Dimethylbutyryl)-1-tetralone (3i): Obtained from 3,3-dimethylbutyric acid **1e** (116 mg, 1 mmol), 1-tetralone **2c** (146 mg, 1 mmol), TFAA (0.85 mL, 6 mmol) and TfOH (44 µL, 0.5 mmol). Yield 57% (140 mg), brown oil. $C_{16}H_{20}O_2$ (244.34): calcd. C 78.65, H 8.25; found C 79.03, H 8.13. ^{1}H NMR (400 MHz, $CDCl_3$): keto-enol (6:94%), enol tautomer, δ 7.99 (d, *J* = 7.6 Hz, 1H, H^{Ar}), 7.41 (t, *J* = 7.2 Hz, 1H, H^{Ar}), 7.33 (t, *J* = 7.2 Hz, 1H, H^{Ar}), 7.20 (d, *J* = 7.3 Hz, 1H, H^{Ar}), 2.85 (t, *J* = 6.6 Hz, 2H, CH_2), 2.66 (t, *J* = 6.6 Hz, 2H, CH_2), 2.40 (s, 2H, *t*-BuCH_2), 1.08 (s, 9H, $C(CH_3)_3$). ^{13}C NMR (100 MHz, $CDCl_3$): δ = 190.40 (CO), 181.76 (C=$\underline{C}$(OH)), 141.09 (C^{Ar}), 131.69 (C^{Ar}+CH^{Ar}), 127.12 (CH^{Ar}), 126.44 (CH^{Ar}), 125.89 (CH^{Ar}), 106.81 ($\underline{C}$=C(OH)), 46.57 (*t*-BuCH_2), 32.41 ($\underline{C}(CH_3)_3$), 29.72 ($C(\underline{C}H_3)_3$), 28.16 (CH_2), 23.44 (CH_2).

2-Acetyl-1-tetralone (3j): Obtained from acetic acid **1g** (0.12 mL, 2.0 mmol), 1-tetralone **2c** (146 mg, 1 mmol), TFAA (0.85 mL, 6 mmol) and TfOH (44 µL, 0.5 mmol). Yield 53% (100 mg), brown solid, mp 56°C (Lit. [3] 54-56 ^{o}C). ^{1}H NMR (400 MHz, $CDCl_3$): keto-enol (6:94%), enol tautomer, δ 7.94 (d, *J* = 7.6 Hz, 1H, H^{Ar}), 7.39 (t, *J* = 7.3 Hz, 1H, H^{Ar}), 7.31 (t, *J* = 7.5 Hz, 1H, H^{Ar}), 7.19 (d, *J* = 7.4 Hz, 1H, H^{Ar}), 2.87 (t, *J* = 7.3 Hz, 2H, CH_2), 2.62 (t, *J* = 7.3 Hz, 2H, CH_2), 2.23 (s, 3H, CH_3). ^{13}C NMR (100 MHz, $CDCl_3$): δ = 193.8 (CO), 177.0 (C=$\underline{C}$(OH)), 140.9 (C^{Ar}), 131.9 (CH^{Ar}), 131.1 (C^{Ar}), 127.6 (CH^{Ar}), 126.8 (CH^{Ar}), 125.8 (CH^{Ar}), 106.0 ($\underline{C}$=C(OH)), 28.2 (CH_2), 23.9 (CH_3), 22.7 (CH_2).

5-Bromo-2-(3,3-dimethylbutyryl)-1-indanone (3k): Obtained from 3,3-dimethylbutyric acid **1e** (116 mg, 1 mmol), 5-bromo-1-indanone **2d** (211 mg, 1 mmol), TFAA (0.85 mL, 6 mmol) and TfOH (0.044 mL, 0.5 mmol). Yield 86% (266 mg), red solid, mp 118-119 °C. $C_{15}H_{17}BrO_2$ (309.21): calcd. C 58.27, H 5.54; found C 57.92, H 5.66. ^{1}H NMR (400 MHz, $CDCl_3$): keto-enol (2:98%), δ = 7.71-7.62 (m, 2H, H^{Ar}), 7.56 (d, *J* = 8.0 Hz, 1H, H^{Ar}), 3.59 (s, 2H, CH_2^{Ind}), 2.29 (s, 2H, CH_2*t*-Bu), 1.09 (s, 9H, $C(C\underline{H}_3)_3$).^{13}C NMR (100 MHz, $CDCl_3$): δ = 192.1 (CO), 178.0 (C=$\underline{C}$(OH)), 149.1 (C^{Ar}), 137.0 (C^{Ar}), 130.5 (CH^{Ar}), 128.6 (CH^{Ar}), 127.5

(CH^{Ar}), 124.1 (CH^{Ar}), 110.8 ($\underline{C}$=C(OH)), 47.2 (*t*-BuCH_2), 32.6 ($\underline{C}(CH_3)$), 30.0 (CH_2^{Ind}), 29.8 (C($\underline{C}H_3)_3$).

4-(1-Adamantyl)-1-phenylbutane-1,3-dione (3l): Obtained from 1-adamantylacetic acid **1d** (194 mg, 1 mmol), acetophenone **2e** (120 mg, 1 mmol), TFAA (0.85 mL, 6 mmol) and TfOH (44 μL, 0.5 mmol). Yield 47% (140 mg), yellow solid, mp 81-82 °C. $C_{20}H_{24}O_2$ (296.41): calcd. C 81.04, H 8.16; found C 81.45, H 8.01. ^{1}H NMR (400 MHz, $CDCl_3$): keto-enol (5:95%), enol tautomer, δ 7.90 (d, J = 7.3 Hz, 2H, H^{Ar}), 7.53 (t, J = 7.2 Hz, 1H, H^{Ar}), 7.46 (t, J = 7.2 Hz, 2H, H^{Ar}), 6.11 (s, 1H, C$\underline{H}$=C(OH)), 2.16 (s, 2H, CH_2Ad), 1.99 (bs, 3H, CH^{Ad}), 1.75-1.55 (m, 12H, CH_2^{Ad}). ^{13}C NMR (100 MHz, $CDCl_3$): δ = 193.2 (CO), 184.8 (CH=$\underline{C}$(OH)), 135.5 (C^{Ar}), 132.2 (CH^{Ar}), 128.5 (CH^{Ar}), 127.1 (CH^{Ar}), 98.4 ($\underline{C}$H=C(OH)), 53.4 (CH_2^{Ad}), 42.8 (CH_2^{Ad}), 36.7 (CH_2^{Ad}), 34.1 (C^{Ad}), 28.7 (CH^{Ad}).

5,5-Dimethyl-1-phenylhexane-1,3-dione (3m): Obtained from 3,3-dimethylbutyric acid **1e** (232 mg, 2 mmol), acetophenone **2e** (120 mg, 1 mmol), TFAA (0.85 mL, 6 mmol) and TfOH (44 μL, 0.5 mmol). Yield 69% (150 mg), yellow oil (Lit. [4] yellow oil). ^{1}H NMR (400 MHz, $CDCl_3$): keto-enol (4:96%), enol tautomer, δ 7.90 (d, J = 7.2 Hz, 2H, H^{Ar}), 7.53 (t, J = 7.2 Hz, 1H, H^{Ar}), 7.46 (t, J = 7.2 Hz, 2H, H^{Ar}), 6.13 (s, 1H, C$\underline{H}$=C(OH)), 2.29 (s, 2H, *t*-BuCH_2), 1.07 (s, 9H, $C(CH_3)_3$). ^{13}C NMR (100 MHz, $CDCl_3$): δ = 193.4 (CO), 184.8 (CH=$\underline{C}$(OH)), 135.1 (C^{Ar}), 131.9 (CH^{Ar}), 128.2 (CH^{Ar}), 126.7 (CH^{Ar}), 97.7 ($\underline{C}$H=C(OH)), 51.9 (*t*-BuCH_2), 31.5 ($\underline{C}(CH_3)_3$), 29.6 (C($\underline{C}H_3)_3$).

5,5-Dimethyl-1-(4-bromophenyl)hexane-1,3-dione (3n): Obtained from 3,3-dimethyl-butyric acid **1e** (116 mg, 1 mmol), 4-bromoacetophenone **2f** (199 mg, 1 mmol), TFAA (0.85 mL, 6 mmol) and TfOH (44 μL, 0.5 mmol). Yield 61% (180 mg), yellow solid, mp 62-63 °C. $C_{14}H_{17}BrO_2$ (297.19): calcd. C 56.58, H 5.77; found C 56.84, H 5.88. ^{1}H NMR (400 MHz, $CDCl_3$): keto-enol (4:96%), enol tautomer, δ 7.76 (d, J = 8.5 Hz, 2H, H^{Ar}), 7.53 (d, J = 8.5 Hz, 1H, H^{Ar}), 6.08 (s, 1H, C$\underline{H}$=C(OH)), 2.28 (s, 2H, *t*-BuCH_2), 1.06 (s, 9H, $C(CH_3)_3$).^{13}C NMR (100 MHz, $CDCl_3$): δ = 193.6 (CO), 183.8 (CH=$\underline{C}$(OH)), 134.0 (C^{Ar}), 131.5 (CH^{Ar}),

128.2 (CH^{Ar}), 126.7 (C^{Ar}), 97.6 (CH=C(OH)), 51.8 (*t*-BuCH_2), 31.6 ($C(CH_3)_3$), 29.6 ($C(CH_3)_3$).

1-(4-Bromophenyl)butane-1,3-dione (3o): Obtained from acetic acid **1g** (0.12 mL, 2.0 mmol), 4-bromoacetophenone **2f** (199 mg, 1 mmol), TFAA (0.85 mL, 6 mmol) and TfOH (133 μL, 1.5 mmol). Yield 41% (100 mg), yellow solid, mp 93°C (Lit. [5] 92.5 °C). ^{1}H NMR (400 MHz, $CDCl_3$): keto-enol (4:96%), enol tautomer, δ 7.76 (d, *J* = 8.7 Hz, 2H, H^{Ar}), 7.59 (d, *J* = 8.7 Hz, 2H, H^{Ar}), 6.14 (s, 1H, CH=C(OH)), 2.20 (s, 3H, CH_3). ^{13}C NMR (100 MHz, $CDCl_3$): δ = 193.4 (CO), 181.9 (CH=C(OH)), 133.4 (C^{Ar}), 131.5 (CH^{Ar}), 128.1 (CH^{Ar}), 126.7 (C^{Ar}), 96.2 (CH=C(OH)), 25.4 (CH_3).

4-(1-Adamantyl)-1-(2-fluorophenyl)butane-1,3-dione (3p): Obtained from 1-adamantylacetic acid **1d** (194 mg, 1 mmol), *o*-fluoroacetophenone **2g** (138 mg, 1 mmol), TFAA (0.85 mL, 6 mmol) and TfOH (44 μL, 0.5 mmol). Yield 48% (150 mg), yellow solid, mp 96-97 °C. $C_{20}H_{23}FO_2$ (314.40): calcd. C 76.41, H 7.37; found C 76.67, H 7.12. ^{1}H NMR (400 MHz, $CDCl_3$): keto-enol (4:96%), enol tautomer, δ 7.96 (m, 1H, H^{Ar}), 7.47 (m, 1H, H^{Ar}), 7.24 (m, 1H, H^{Ar}), 7.12 (m, 1H, H^{Ar}), 6.20 (d, *J* = 1.6 Hz, 1H, CH=C(OH), 2.16 (s, 2H, CH_2Ad), 1.98 (bs, 3H, CH^{Ad}), 1.75-1.59 (m, 12H, CH_2^{Ad}). ^{13}C NMR (100 MHz, $CDCl_3$): δ = 194.1 (CO), 180.0 (d, CH=C(OH)), 160.7 (d, C^{Ar}), 133.0 (d, CH^{Ar}), 129.8 (CH^{Ar}), 124.0 (d, CH^{Ar}), 123.4 (d, C^{Ar},), 116.1 (d, CH^{Ar}), 102.8 (d, CH=C(OH)), 53.1 (CH_2Ad), 42.4 (CH_2^{Ad}), 36.3 (CH_2^{Ad}), 33.8 (C^{Ad}), 28.4 (CH^{Ad}).

4-(1-Adamantyl)-1-(4-methoxyphenyl)butane-1,3-dione (3q): Obtained from 1-adamantylacetic acid **1d** (194 mg, 1 mmol), 4-methoxyacetophenone **2h** (150 mg, 1 mmol), TFAA (0.85 mL, 6 mmol) and TfOH (44 μL, 0.5 mmol). Yield 37% (120 mg), yellow solid, mp 80-82 °C. $C_{21}H_{26}O_3$ (326.44): calcd. C 77.27, H 8.03; found C 77.62, H 7.89. ^{1}H NMR (400 MHz, $CDCl_3$): keto-enol (4:96%), enol tautomer, δ 7.88 (d, *J* = 8.8 Hz, 2H, H^{Ar}), 6.95 (d, *J* = 8.8 Hz, 2H, H^{Ar}), 6.04 (s, 1H, CH=C(OH)), 3.87 (s, 3H, OCH_3), 2.12 (s, 2H, CH_2Ad), 1.98 (bs, 3H, CH^{Ad}), 1.75-1.59 (m, 12H, CH_2^{Ad}). ^{13}C NMR (100 MHz, $CDCl_3$) δ = 190.7

(CO), 185.0 (CH=C(OH)), 162.7 (C^{Ar}), 128.8 (CH^{Ar}), 127.8 (C^{Ar}), 113.5 (CH^{Ar}), 97.2 (CH=C(OH)), 55.0 (OCH_3), 52.7 (CH_2Ad), 42.4 (CH_2^{Ad}), 36.4 (CH_2^{Ad}), 33.6 (C^{Ad}), 28.3 (CH^{Ad}).

4-(1-Adamantyl)-1-(4-isopropylphenyl)butane-1,3-dione (3r): Obtained from 1-adamantylacetic acid **1d** (194 mg, 1 mmol), 4-*iso*-propylacetophenone **2i** (162 mg, 1 mmol), TFAA (0.85 mL, 6 mmol) and TfOH (44 μL, 0.5 mmol). Yield 49% (166 mg), yellow solid, mp 89-90 °C. $C_{23}H_{30}O_2$ (338.49): calcd. C 81.61, H 8.93; found C 81.40, H 8.99. ^{1}H NMR (400 MHz, $CDCl_3$): keto-enol (4:96%), enol tautomer, δ 7.84 (d, *J* = 8.3 Hz, 2H, H^{Ar}), 7.31 (d, *J* = 8.2 Hz, 2H, H^{Ar}), 6.08 (s, 1H, CH=C(OH)), 2.97 (m, 1H, $CH(CH_3)_2$), 2.15 (s, 2H, CH_2Ad), 1.98 (bs, 3H, CH^{Ad}), 1.77-1.50 (m, 12H, CH_2^{Ad}), 1.28 (d, *J* = 6.9 Hz, 6H, $CH(CH_3)_2$). ^{13}C NMR (100 MHz, $CDCl_3$) δ = 192.2 (CO), 184.7 (CH=C(OH)), 153.4 (C^{Ar}), 132.8 (C^{Ar}), 126.9 (CH^{Ar}), 126.3 (CH^{Ar}), 97.8 (CH=C(OH)), 53.0 (CH_2Ad), 42.4 (CH_2^{Ad}), 36.4 (CH_2^{Ad}), 33.8 ($CH(CH_3)_2$), 33.7 (C^{Ad}), 28.4 (CH^{Ad}), 23.3 ($CH(CH_3)_2$).

4-(1-Adamantyl)-1-(2-thienyl)butane-1,3-dione (3s): Obtained from 1-adamantylacetic acid **1d** (194 mg, 1 mmol), 2-acetylthiophene **2j** (126 mg, 1 mmol), TFAA (0.85 mL, 6 mmol) and TfOH (44 μL, 0.5 mmol). Yield 43% (130 mg), brown solid, mp 81-82 °C. $C_{18}H_{22}O_2S$ (302.44): calcd. C 71.49, H 7.33, S 10.60; found C 71.87, H 7.26, S 10.39. ^{1}H NMR (400 MHz, $CDCl_3$): keto-enol (4:96%), enol tautomer, δ 7.70 (d, *J* = 3.5 Hz, 1H, H^{Ar}), 7.60 (d, *J* = 4.8 Hz, 1H, H^{Ar}), 7.13 (t, *J* = 4.3 Hz, 1H, H^{Ar}), 5.94 (s, 1H, CH=C(OH)), 2.09 (s, 2H, CH_2Ad), 1.98 (bs, 3H, CH^{Ad}), 1.75-1.59 (m, 12H, CH_2^{Ad}). ^{13}C NMR (100 MHz, $CDCl_3$): δ = 186.9 (CO), 182.2 (CH=C(OH)), 142.0 (C^{Ar}), 132.0 (CH^{Ar}), 129.7 (CH^{Ar}), 127.8 (CH^{Ar}), 97.8 (CH=C(OH)), 51.5 (CH_2Ad), 42.4 (CH_2^{Ad}), 36.3 (CH_2^{Ad}), 33.7 (C^{Ad}), 28.3 (CH^{Ad}).

1-(1-Adamantyl)-3-phenylpentane-2,4-dione (3t): Obtained from 1-adamantylacetic acid **1d** (194 mg, 1 mmol), phenylacetone **2k** (134 mg, 1 mmol), TFAA (0.85 mL, 6 mmol) and TfOH (44 μL, 0.5 mmol). Yield 64% (200 mg), brown oil. $C_{21}H_{26}O_2$ (310.44): calcd. C 81.25, H 8.44; found C 81.65, H 8.53. ^{1}H NMR (400 MHz, $CDCl_3$): keto-enol (8:92%), enol tautomer,

δ = 7.43-7.27 (m, 3H, H^{Ar}), 7.14 (d, *J* = 7.2 Hz, 2H, H^{Ar}), 2.01-1.84 (m, 8H, CH_3+CH_2+CH^{Ad}), 1.73-1.43 (m, 12H, CH_2^{Ad}). ^{13}C NMR (100 MHz, $CDCl_3$): δ =194.5 (CO), 188.7 (C=<u>C</u>(OH)), 136.8 (C^{Ar}), 131.4 (CH^{Ar}), 128.1 (CH^{Ar}), 126.9 (CH^{Ar}), 116.1 (<u>C</u>=C(OH)), 47.9 (CH_2Ad), 42.4 (CH_2^{Ad}), 36.3 (CH_2^{Ad}), 34.2 (C^{Ad}), 28.3 (CH^{Ad}), 25.2 (CH_3).

2-[3-(*N*-Trifluroacetylamino)propionyl]-1-indanone (3u): Obtained from β-alanine **1i** (134 mg, 1.5 mmol), 1-indanone **2a** (132 mg, 1 mmol), TFAA (0.85 mL, 6 mmol) and TfOH (88 μL, 1 mmol). Yield 57% (171 mg), brown solid, mp 139-141 °C. $C_{14}H_{12}F_3NO_3$ (299.25): calcd. C 56.19, H 4.04, N 4.47; found C 56.53, H 4.18, N 4.66. ^{1}H NMR (400 MHz, $CDCl_3$): keto-enol (30:70%), enol tautomer, δ 7.81 (d, *J* = 7.6 Hz, 1H, H^{Ar}), 7.60-7.49 (m, 2H, H^{Ar}), 7.47-7.38 (m, 1H), 7.13 (bs, 1H, NH), 3.80-3.73 (m, 2H, CH_2), 3.59 (s, 2H, CH_2^{Ind}), 2.82 (t, *J* = 6.0 Hz, 2H, CH_2). ^{13}C NMR (100 MHz, acetone-d_6): 188.7 (CO), 180.1 (C=<u>C</u>(OH)), 147.5 (C^{Ar}), 137.6 (C^{Ar}), 132.8 (CH^{Ar}), 127.4 (CH^{Ar}), 125.9 (CH^{Ar}), 122.4 (CH^{Ar}), 111.3 (<u>C</u>=C(OH)), 35.9 (CH_2), 34.0 (CH_2), 29.8 (CH_2).

2-[3-(*N*-Trifluoroacetylamino)propionyl]-1-tetralone (3v): Obtained from β-alanine **1i** (134 mg, 1.5 mmol), 1-tetralone **2c** (146 mg, 1 mmol), TFAA (0.85 mL, 6 mmol) and TfOH (88 μL, 1 mmol). Yield 51% (160 mg), brown solid, mp 96-97°C. $C_{15}H_{14}F_3NO_3$ (313.28): calcd. C 57.51, H 4.50, N 4.47; found C 57.23, H 4.66, N 4.40. ^{1}H NMR (400 MHz, $CDCl_3$): keto-enol (3:97%), enol tautomer, δ 7.92 (d, *J* = 7.6 Hz, 1H, H^{Ar}), 7.41 (t, *J* = 7.4 Hz, 1H, H^{Ar}), 7.32 (t, *J* = 7.4 Hz, 1H, H^{Ar}), 7.21 (d, *J* = 7.2 Hz, 1H, H^{Ar}), 3.73 (m, 2H, CH_2), 2.92-2.79 (m, 4H, CH_2+CH_2), 2.58 (t, *J* = 7.1 Hz, 2H, CH_2). ^{13}C NMR (100 MHz, $CDCl_3$): δ = 196.5 (CO), 173.9 (C=<u>C</u>(OH)), 156.8 (q, <u>C</u>OCF$_3$), 140.1 (C^{Ar}), 131.8 (CH^{Ar}), 129.7 (C^{Ar}), 127.2 (CH^{Ar}), 126.5 (CH^{Ar}), 125.3 (CH^{Ar}), 115.4 (q, CO<u>C</u>F$_3$), 105.5 (<u>C</u>=C(OH)), 35.3 (CH_2), 34.6 (CH_2), 27.5 (CH_2), 21.3 (CH_2).

5-(*N*-Trifluoroacetylamino)-1-phenylpentan-1,3-dione (3w): Obtained from β-alanine **1i** (134 mg, 1.5 mmol), acetophenone **2e** (0.120 mg, 1 mmol), TFAA (0.85 mL, 6 mmol) and TfOH (88 μL, 1 mmol). Yield 15% (43 mg), yellow solid, mp 103 °C. $C_{13}H_{12}F_3NO_3$ (287.24):

calcd. C 54.36, H 4.21, N 4.88; found C 54.80, H 4.24, N 4.77. ^{1}H NMR (400 MHz, $CDCl_3$): keto-enol (4:96%), enol tautomer, δ 7.87 (d, *J* = 8.1 Hz, 2H, H^{Ar}), 7.58-7.42 (m, 3H, H^{Ar}), 7.18 (bs, 1H, NH), 6.18 (s, 1H, C<u>H</u>=C(OH)), 3.71 (m, 2H, CH_2), 2.79 (t, *J* = 5.6 Hz, 2H, CH_2). ^{13}C NMR (100 MHz, $CDCl_3$): δ = 196.2 (CO), 180.5 (CH=<u>C</u>(OH)), 156.8 (q, <u>C</u>OCF_3), 133.3 (C^{Ar}), 132.2 (CH^{Ar}), 128.3 (CH^{Ar}), 126.5 (CH^{Ar}), 115.4 (q, CO<u>C</u>F_3), 96.2 (<u>C</u>H=C(OH)), 37.7 (CH_2), 35.1 (CH_2).

2-[2-(3-Trifluoroacetoxy-1-adamantyl)acetyl]-1-tetralone (3x): Obtained from 4-phenylbutyric acid **1c** (164 mg, 1 mmol), 3-hydroxy-1-adamantylacetic acid **1k** (210 mg, 1 mmol), TFAA (0.85 mL, 6 mmol) and TfOH (0.44 µL, 0.5 mmol) as described for **3h**. Yield 51% (220 mg), brown oil. $C_{24}H_{25}F_3O_4$ (434.46): calcd. C 66.35, H 5.80; found C 66.01, H 5.71. ^{1}H NMR (400 MHz, $CDCl_3$): keto-enol (4:96%), enol tautomer, δ 7.98 (d, *J* = 7.7 Hz, 1H, H^{Ar}), 7.41 (t, *J* = 7.4 Hz, 1H, H^{Ar}), 7.33 (t, *J* = 7.5 Hz, 1H, H^{Ar}), 7.20 (d, *J* = 7.4 Hz, 1H, H^{Ar}), 2.85 (t, *J* = 7.0 Hz, 2H, H), 2.63 (t, *J* = 7.0 Hz, 2H, H), 2.38 (s, 2H, CH_2Ad), 2.30 (bs, 2H, CH^{Ad}), 2.22-2.05 (m, 12H, CH_2^{Ad}). ^{13}C NMR (100 MHz, $CDCl_3$): δ = 188.4 (CO), 182.6 (C=<u>C</u>(OH)), 155.8 (q, <u>C</u>OCF_3), 141.5 (C^{Ar}), 132.3 (CH^{Ar}), 131.9 (C^{Ar}), 127.6 (CH^{Ar}), 126.9 (CH^{Ar}), 126.4 (CH^{Ar}), 114.3 (q, CO<u>C</u>F_3), 107.5 (<u>C</u>=C(OH)), 86.9 (C^{Ad}), 46.7 (CH_2Ad), 45.8 (CH_2^{Ad}), 41.0 (CH_2^{Ad}), 39.9 (CH_2^{Ad}), 38.5 (CH_2^{Ad}), 34.9 (C^{Ad}), 30.8 (CH^{Ad}) 28.5 (CH_2), 23.9 (CH_2).

2-[2-(3-Hydroxy-1-adamantyl)acetyl]-1-tetralone (3y). The mixture of **3x** (434 mg, 1 mmol), ethanol (9 mL) and 1N NaOH (1 mL) was heated with stirring at 60°C for ~2 h. When the reaction went to completion (TLC control), solvent was evaporated, the residue was acidified with 1N HCl (pH ~5) and extracted with CH_2Cl_2. The organic phase was washed with H_2O, dried ($MgSO_4$) and concentrated under vacuum. Purification of the product was performed by column chromatography on silica gel using CH_2Cl_2/MeOH as eluent. Yield 81% (275 mg), brown solid, mp 102-104°C. $C_{22}H_{26}O_3$ (338.45): calcd. C 78.07, H 7.74; found C 77.84, H 7.59. ^{1}H NMR (400 MHz, $CDCl_3$): keto-enol (5:95%), enol tautomer, δ = 7.99 (d,

J = 7.6 Hz, 1H, H^{Ar}), 7.41 (t, *J* = 7.4 Hz, 1H, H^{Ar}), 7.33 (t, *J* = 7.4 Hz, 1H, H^{Ar}), 7.20 (d, *J* = 7.4 Hz, 1H, H^{Ar}), 2.85 (t, *J* = 7.0 Hz, 2H, CH_2), 2.65 (t, *J* = 7.0 Hz, 2H, CH_2), 2.35 (s, 2H, CH_2Ad), 2.21 (bs, 2H, CH^{Ad}), 1.72-1.47 (m, 12H, CH_2^{Ad}). ^{13}C NMR (100 MHz, $CDCl_3$): δ = 188.6 (CO), 182.2 (C=$\underline{C}$(OH)), 141.2 (C^{Ar}), 131.2 (CH^{Ar}), 131.2 (C^{Ar}), 127.2 (CH^{Ar}), 126.5 (CH^{Ar}), 126.0 (CH^{Ar}), 107.1 ($\underline{C}$=C(OH)), 68.6 (C^{Ad}), 49.9 (CH_2Ad), 46.6 (CH_2^{Ad}), 44.0 (CH_2^{Ad}), 41.1 (CH_2^{Ad}), 37.8 (CH_2^{Ad}), 34.8 (C^{Ad}), 30.3 (CH^{Ad}), 28.2 (CH_2), 23.6 (CH_2).

1,3-Di(indanone-1-yl-2)adamantane (3z): Obtained from 1,3-di(carboxymethyl)adamantane **1l** (126 mg, 0.5 mmol), 1-indanone **2a** (132 mg, 1 mmol), TFAA (0.85 mL, 6 mmol) and TfOH (44 μL, 0.5 mmol). Yield 96% (230 mg), yellow solid, mp 152-154 ºC. $C_{32}H_{32}O_4$ (480.61): calcd. C 79.97, H 6.71; found C 80.24, H 6.68. ^{1}H NMR (400 MHz, $CDCl_3$): keto-enol (4:96%), enol tautomer, δ = 7.81 (d, *J* = 7.6 Hz, 2H, H^{Ar}), 7.53-7.45 (m, 2H, H^{Ar}), 7.42-7.33 (m, 4H, H^{Ar}), 3.54 (s, 4H, CH_2^{Ind}), 2.18 (s, 4H, CH_2Ad), 2.07 (bs, 2H, CH^{Ad}), 1.72-1.54 (m, 12H, CH_2^{Ad}). ^{13}C NMR (100 MHz, $CDCl_3$): δ = 193.8 (CO), 176.50 (C=$\underline{C}$(OH)), 147.9 (C^{Ar}), 138.3 (C^{Ar}), 132.9 (CH^{Ar}), 127.2 (CH^{Ar}), 125.6 (CH^{Ar}), 123.2 (CH^{Ar}), 111.8 ($\underline{C}$=C(OH)), 48.3 (CH_2^{Ad}), 48.1 (CH_2Ad), 42.1 (CH_2^{Ad}), 35.8 (C^{Ad}), 35.7 (CH_2^{Ad}), 30.6 (CH_2), 29.0 (CH^{Ad}).

1-Hydroxy-4-aza-2,3,9,10-tetrahydrophenanthrene hydrochloride (4b): Obtained from diketone **3v** (313 mg, 1 mmol). Yield 94% (222 mg), yellow solid, mp 153-154 ºC. $C_{13}H_{13}NO$•HCl (235.71): calcd. C 66.24, H 5.99, N 5.94; found C 66.57, H 6.03, N 5.87. ^{1}H NMR (400 MHz, methanol-d_4): δ = 7.87 (d, *J* = 7.9 Hz, 1H, H^{Ar}), 7.64 (t, *J* = 7.6 Hz, 1H, H^{Ar}), 7.50-7.41 (m, 2H, H^{Ar}), 3.89 (t, *J* = 8.9 Hz, 2H, CH_2), 2.90 (m, 4H, CH_2), 2.67 (t, *J* = 6.9 Hz, 2H, CH_2). ^{13}C NMR (100 MHz, methanol-d_4): δ = 176.5 (C), 167.9 (C), 144.9 (C), 135.6 (CH), 130.4 (CH), 128.7 (CH), 127.5 (C), 126.9 (CH), 105.3 (C), 40.9 (CH_2), 29.0 (CH_2), 28.3 (CH_2), 20.1 (CH_2).

7-Bromo-3-[2-(4-bromophenyl)-ethyl]-1,4-dihydroindeno[1,2-*c*]pyrazole (6b): Obtained from β-(4-bromophenyl)propionic acid **1b** (229 mg, 1 mmol), TFAA (0.85 mL, 6 mmol),

TfOH (44 μL, 0.5 mmol) and N_2H_4 H_2O (0.1 mL, 2 mmol) as described for **6a**. Yield 72% (150 mg), red solid, m.p. 172-174 °C. $C_{18}H_{14}Br_2N_2$ (418.13): calcd. C 51.71, H 3.37, N 6.70; found C 51.52, H 3.24, N 6.85. ^1H NMR (400 MHz, $CDCl_3$): δ = 8.50 (bs, 1H, NH), 7.82 (s, 1H, H^{Ar}), 7.45-7.28 (m, 3H, H^{Ar}), 7.01 (d, *J* = 8.3 Hz, 2H, H^{Ar}), 3.36 (s, 2H, CH_2), 3.08-2.92 (m, 4H, CH_2).^{13}C NMR (100 MHz, $CDCl_3$): δ = 146.8 (C), 139.0 (C), 135.9 (C), 131.2 (2CH), 131.2 (C), 129.6 (2CH), 129.0 (CH), 126.8 (CH), 122.7 (CH), 120.5 (C), 119.8 (C), 33.7 (CH_2), 27.7 (CH_2), 26.9 (CH_2).

3-[(1-Adamantyl)methyl]-1,4-dihydroindeno[1,2-*c*]pyrazole (6c): A mixture of **1a** (150 mg, 1 mmol), TFAA (0.85 mL, 6 mmol) and CH_2Cl_2 (1 mL) was stirred for 15 min at rt and then TfOH (133 μL, 1.5 mmol) was added. The reaction mixture was kept for 30 min (1-indanone **2a** synthesis) and then 1-adamantylacetic acid **1d** (194 mg, 1 mmol) was added. On completion of the reaction (~1.5 h), solvent was removed under reduced pressure and then as described for **6a**. Yield 62% (190 mg), red solid, m.p. 206-207 °C. $C_{21}H_{24}N_2$ (304.44): calcd. C 82.85, H 7.95, N 9.20; found C 82.58, H 7.72, N 9.32. ^1H NMR (400 MHz, $CDCl_3$): δ = 7.77 (d, *J* = 7.4 Hz, 1H, H^{Ar}), 7.48 (d, *J* = 7.3 Hz, 1H, H^{Ar}), 7.34 (t, *J* = 7.4 Hz, 1H, H^{Ar}), 7.27 (t, *J* = 7.4 Hz, 1H, H^{Ar}), 3.59 (s, 2H, CH_2), 2.46 (s, 2H, CH_2Ad), 1.90 (bs, 3H, CH^{Ad}), 1.72-1.45 (m, 12H, CH_2^{Ad}). ^{13}C NMR (100 MHz, $CDCl_3$): δ = 148.2 (C), 134.9 (C), 134.7 (C), 126.4 (CH), 126.0 (CH), 125.4 (CH), 122.4 (C), 119.7 (CH), 42.0 (CH_2^{Ad}), 40.1 (CH_2Ad), 36.3 (CH_2^{Ad}), 34.1 (C^{Ad}), 28.7 (CH_2), 28.2 (CH^{Ad}).

3-Methyl-1,4-dihydroindeno[1,2-*c*]pyrazole (6d): Obtained from acetic acid **1g** (0.12 mL, 2.0 mmol), 1-indanone **2a** (132 mg, 1 mmol), TFAA (0.85 mL, 6 mmol), TfOH (44 μL, 0.5 mmol) and N_2H_4 H_2O (0.1 mL, 2 mmol) as described for **6a**. Yield 76% (130 mg), red solid, mp 182-183 ºC (Lit. [6] 183-184 ºC). ^1H NMR (400 MHz, $CDCl_3$): δ = 7.70 (d, *J* = 7.2 Hz, 1H, H^{Ar}), 7.49 (d, *J* = 7.0 Hz, 1H, H^{Ar}), 7.36-7.24 (m, 2H, H^{Ar}), 3.55 (s, 2H, CH_2), 2.40 (s,

3H, CH_3). ^{13}C NMR (100 MHz, $CDCl_3$): δ = 148.4 (C), 134.3 (C), 134.2 (C), 133.7 (C), 126.5 (CH), 126.4 (CH), 125.5 (CH), 121.7 (C), 119.8 (CH), 27.7 (CH_2), 10.2 (CH_3).

References

1. Görlitzer, K. *Arch. Pharm.* **1975,** *308*, 394-397. doi: 10.1002/ardp.19753080514

2. Katritzky, A. R.; Pastor, A. *J. Org. Chem*. **2000**, *65*, 3679-3682. doi: 10.1021/jo991878f

3. Minami, N.; Suzuki, Y. *Yakugaku Zasshi* **1975**, *95*, 815-821. PMID 1237583

4. Lim, D.; Fang, F.; Zhou, G.; Coltart, D. M. *Org. Lett*. **2007**, *9*, 4139-4142. doi: 10.1021/ol701599v

5. Auwers, K. V.; Heimke, P. *Liebigs Ann. Chem*. **1927,** *458*, 186-220. doi: 10.1002/jlac.19274580112

6. Braun, R. A.; Mosher, W. A. *J. Am. Chem. Soc.* **1958**, *80*, 4919-4921. doi: 10.1021/ja01551a039

3. Molecular structures of compounds 3c, 3u, 4a, and 6b (Fig. S1-S4).

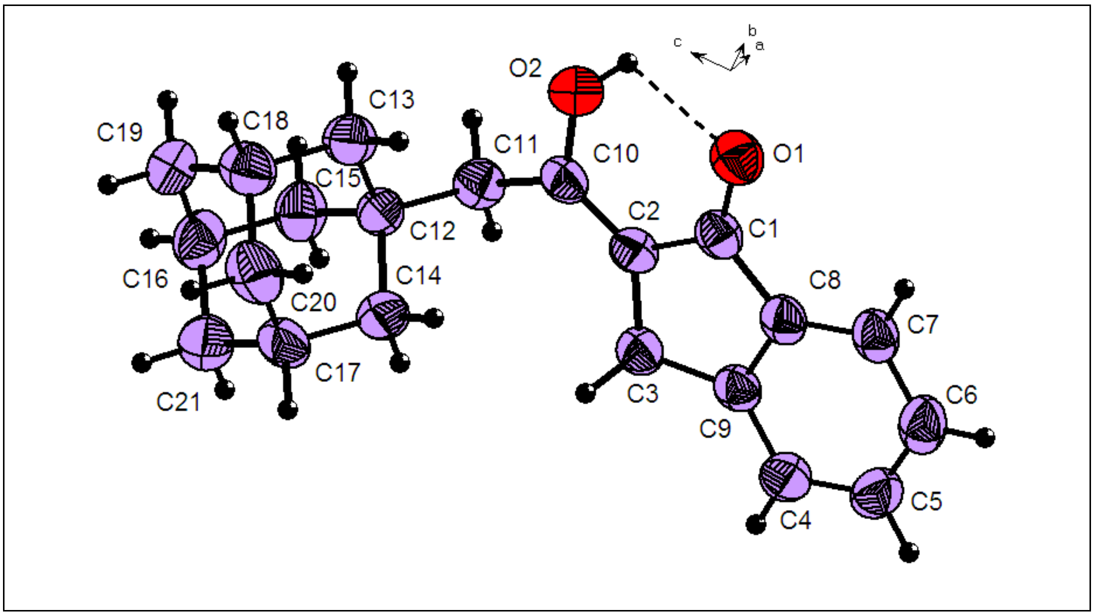

Figure S-1. Molecular structure of 2-[2-(1-adamantyl)acetyl]-1-indanone **3c** (CCDC 942538).

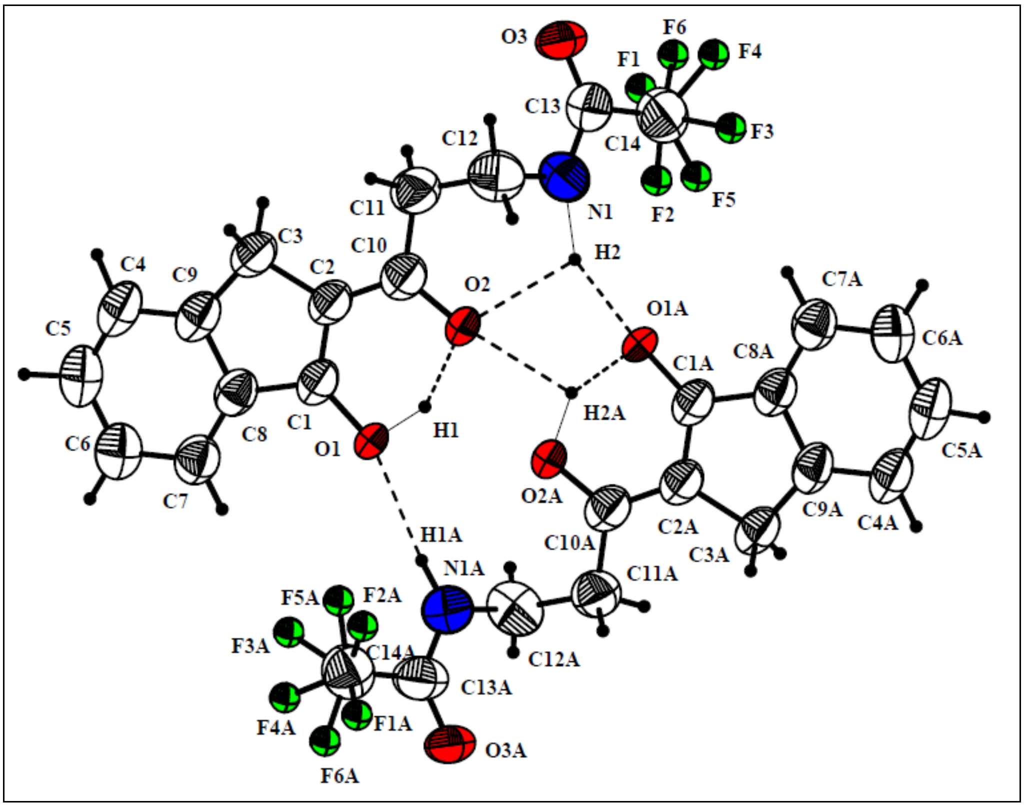

Figure S-2. Two independent molecules in the crystal structure of 2-[3-(*N*-trifluroacetylamino)propionyl]-1-indanone **3u** (CCDC 942537). The dash and thin lines show the shortest distance between nitrogen (N1, N1a), oxygen (O1, O1a, O2, O2a) and hydrogen (H1, H2, H2a) atoms. Fluorine atoms of the trifluoromethyl groups are disordered.

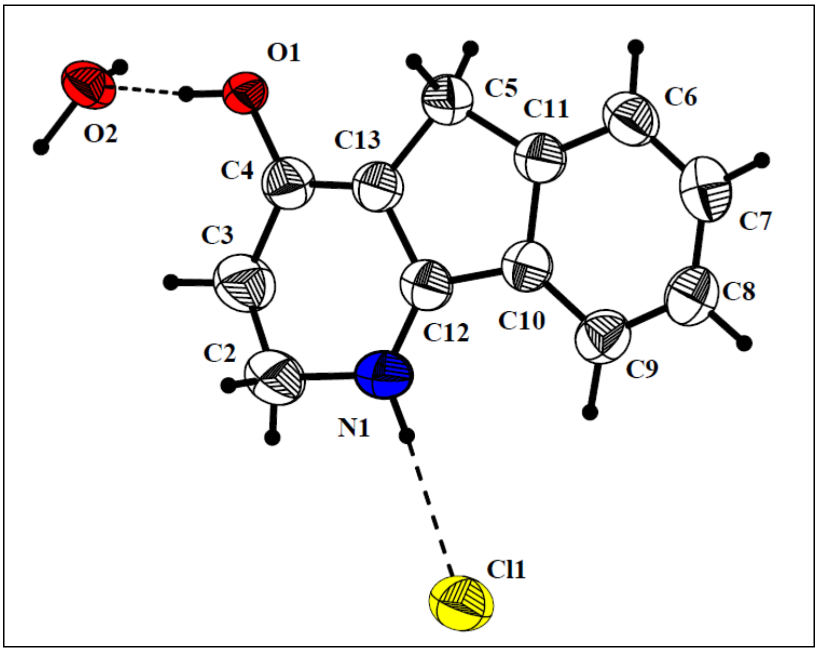

Figure S-3. Molecular structure of 1-hydroxy-4-aza-2,3-dihydrofluorene **4a** (CCDC 942536).

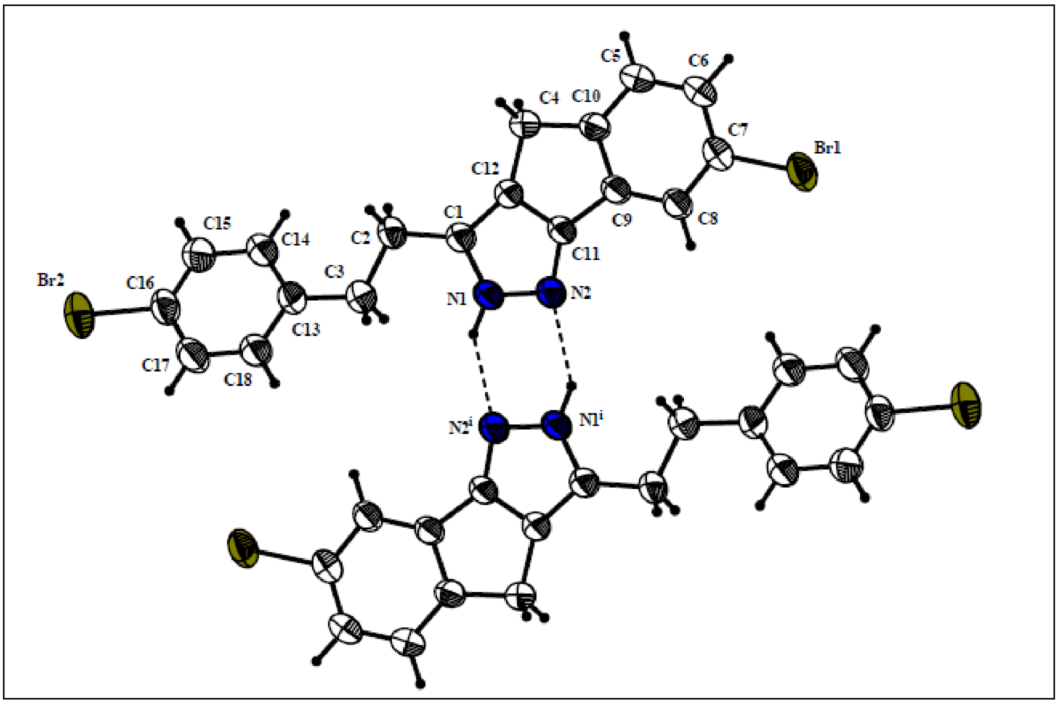

Figure S-4. Part of the crystal structure of 7-bromo-3-[2-(4-bromophenyl)-ethyl]-1,4-dihydroindeno[1,2-*c*]pyrazole **6b** (CCDC 950017) showing centrosymmetric dimer as result of hydrogen bonding N1-H1…N2^{i}.

4. Copies of ^{1}H, ^{13}C and ^{31}P spectra (Fig. S5-S69).

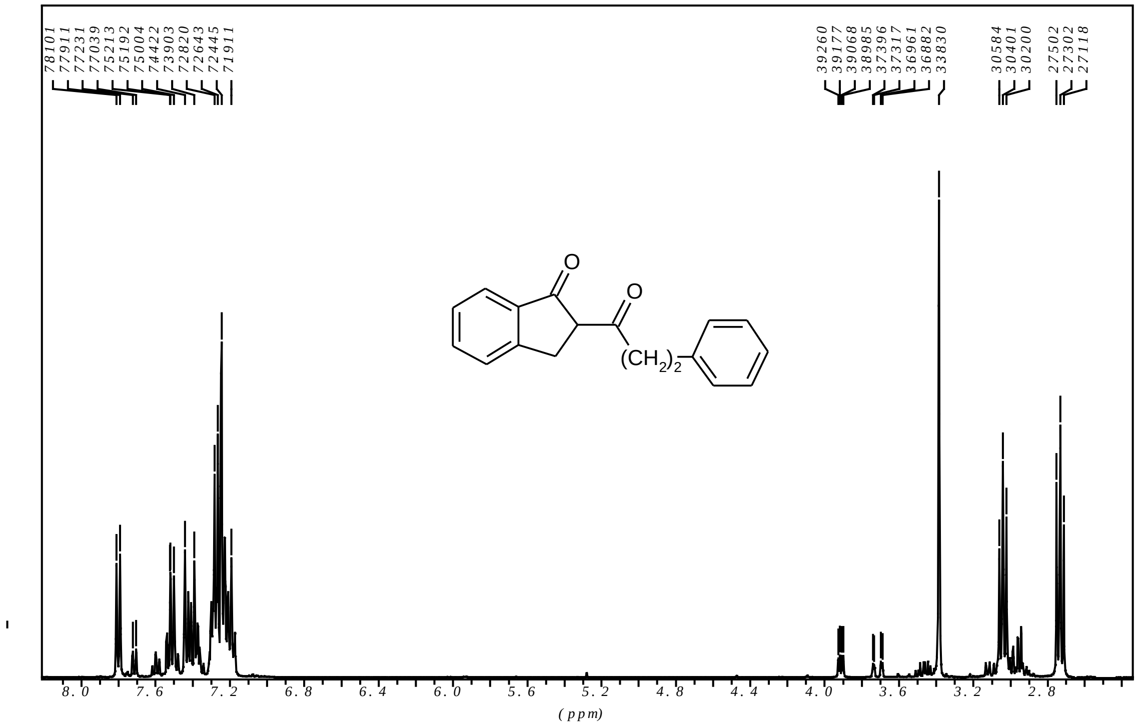

Figure S-5. ^{1}H NMR spectrum of 2-(3-phenylpropionyl)-1-indanone **3a**.

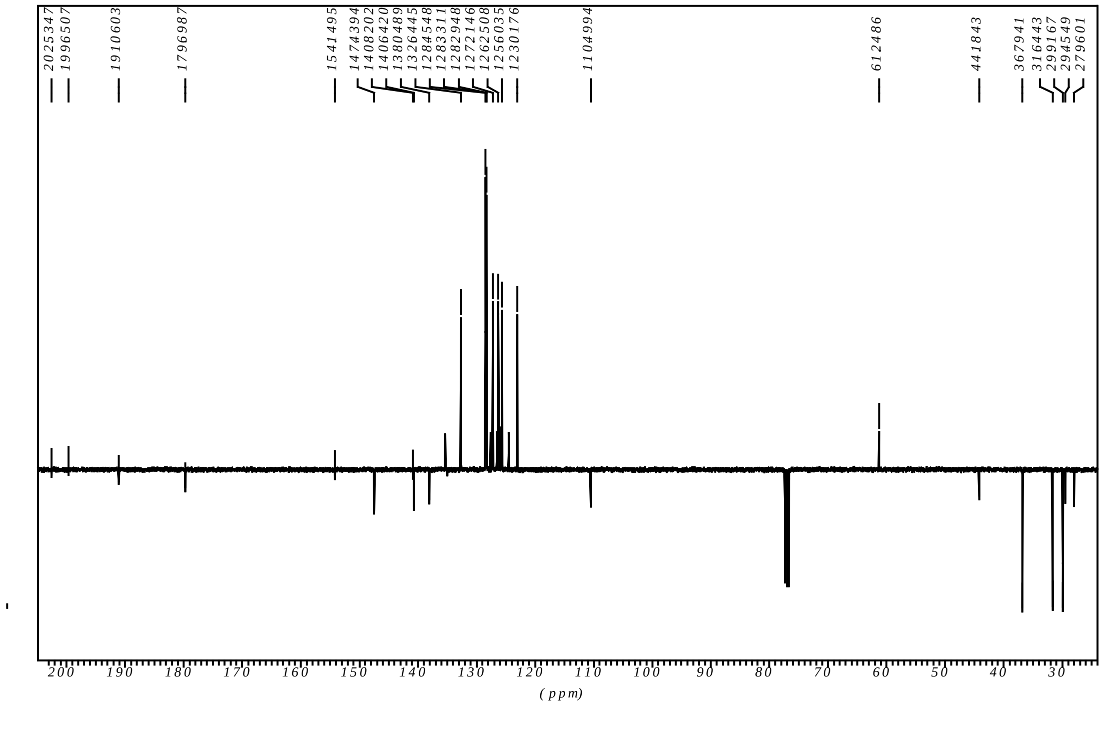

Figure S-6. ^{13}C NMR spectrum of 2-(3-phenylpropionyl)-1-indanone **3a**.

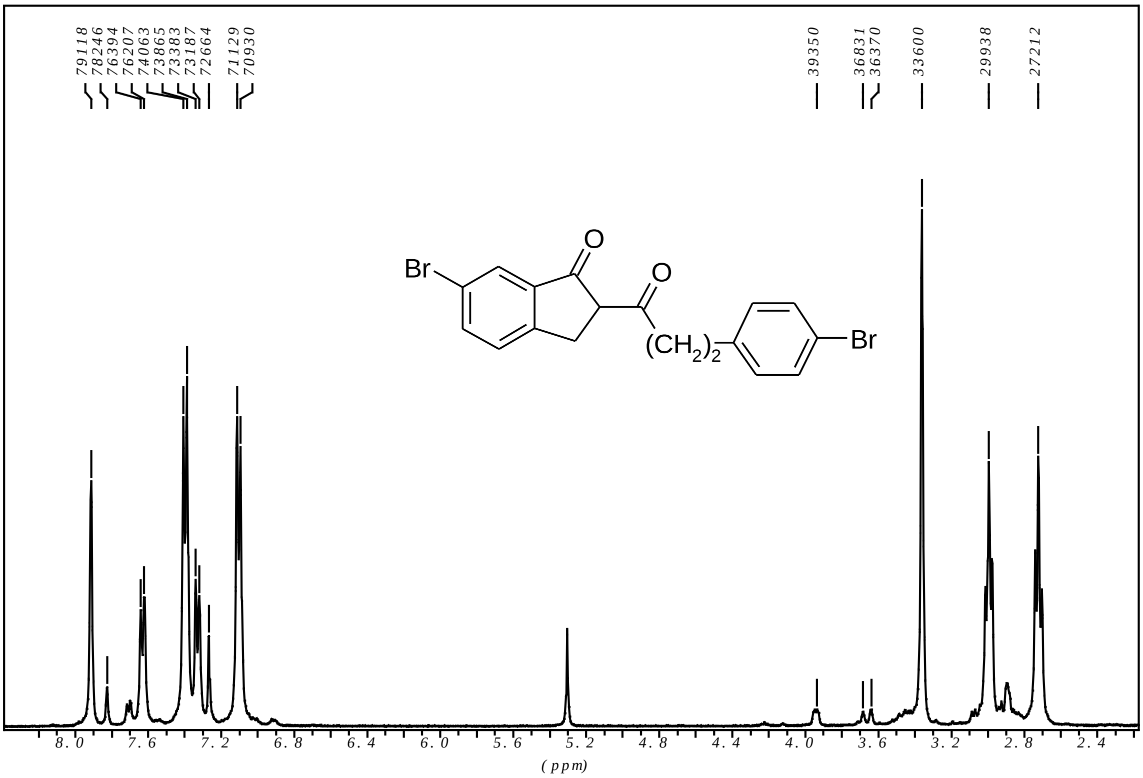

Figure S-7. ^{1}H NMR spectrum of 6-bromo-2-[3-(4-bromophenyl)propionyl]-1-indanone **3b**.

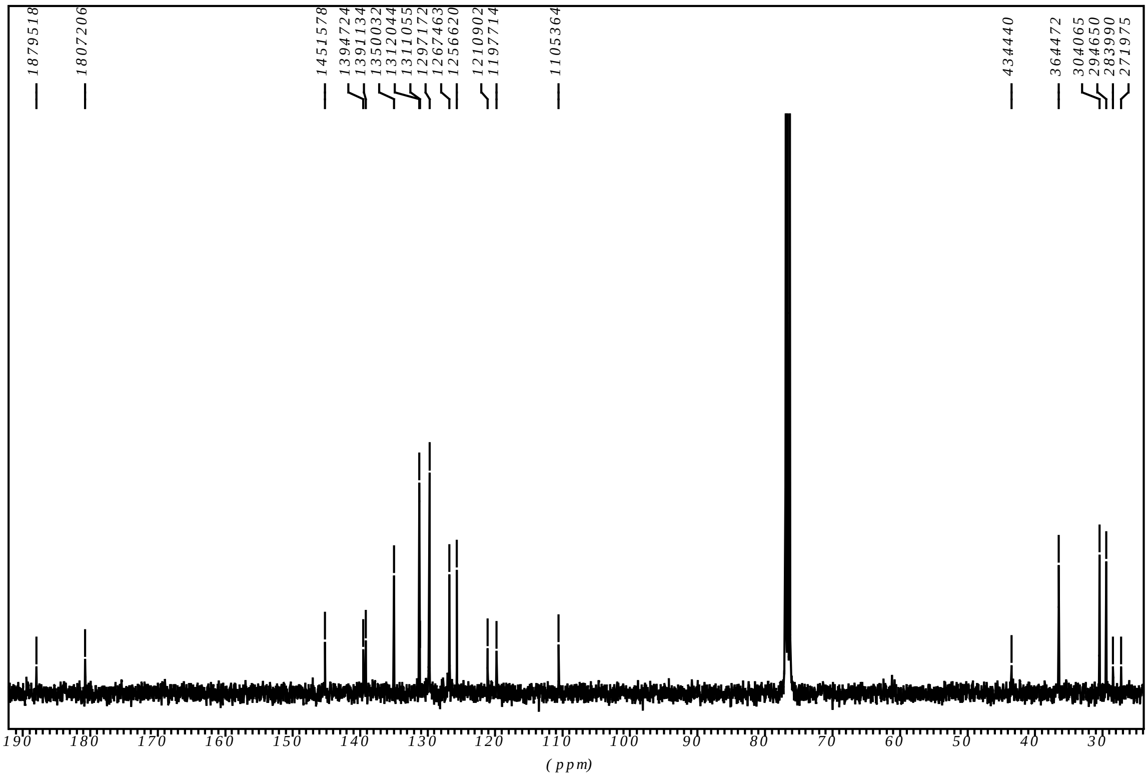

Figure S-8. ^{13}C NMR spectrum of 6-bromo-2-[3-(4-bromophenyl)propionyl]-1-indanone **3b**.

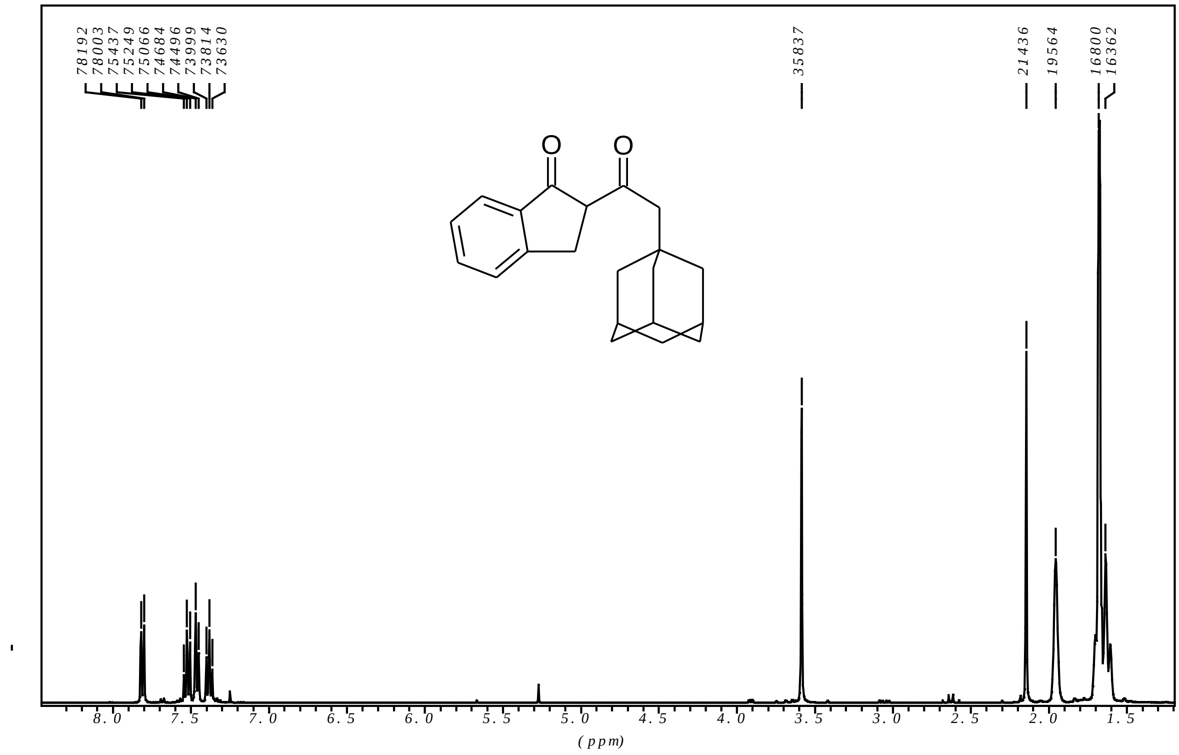

Figure S-9. ^{1}H NMR spectrum of 2-[2-(1-adamantyl)acetyl]-1-indanone **3c**.

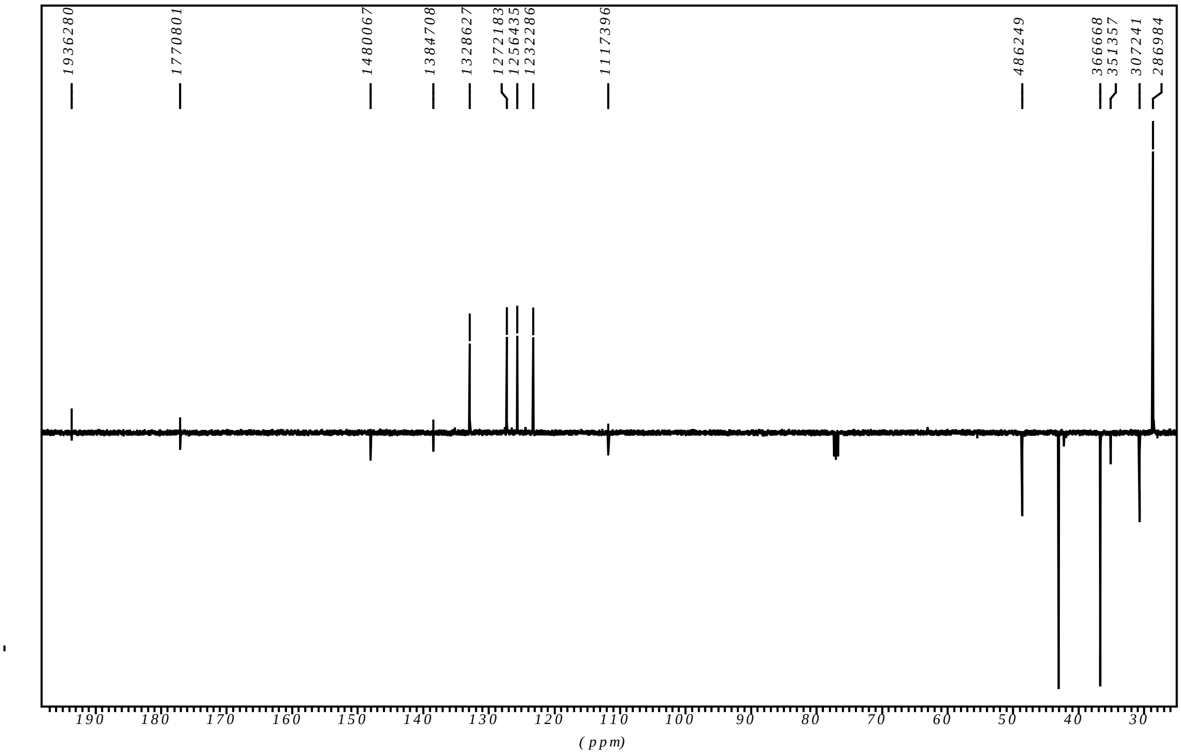

Figure S-10. ^{13}C NMR spectrum of 2-[2-(1-adamantyl)acetyl]-1-indanone **3c**.

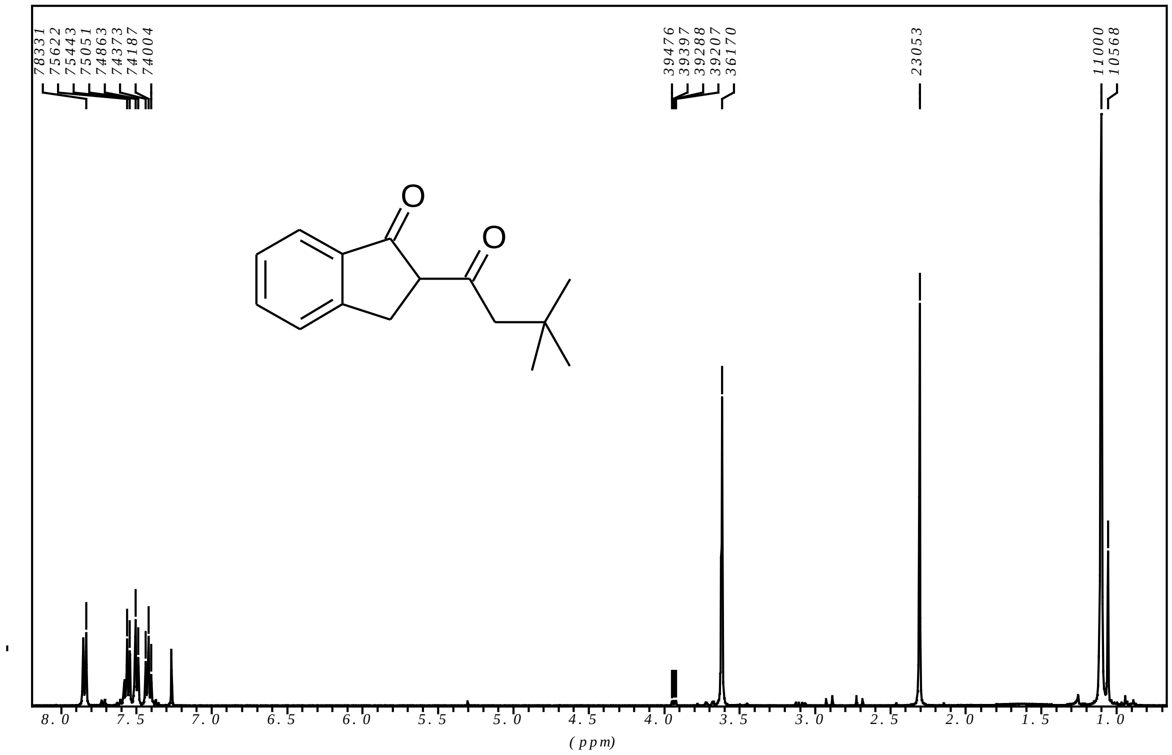

Figure S-11. ^{1}H NMR spectrum of 2-(3,3-dimethylbutyryl)-1-indanone **3d**.

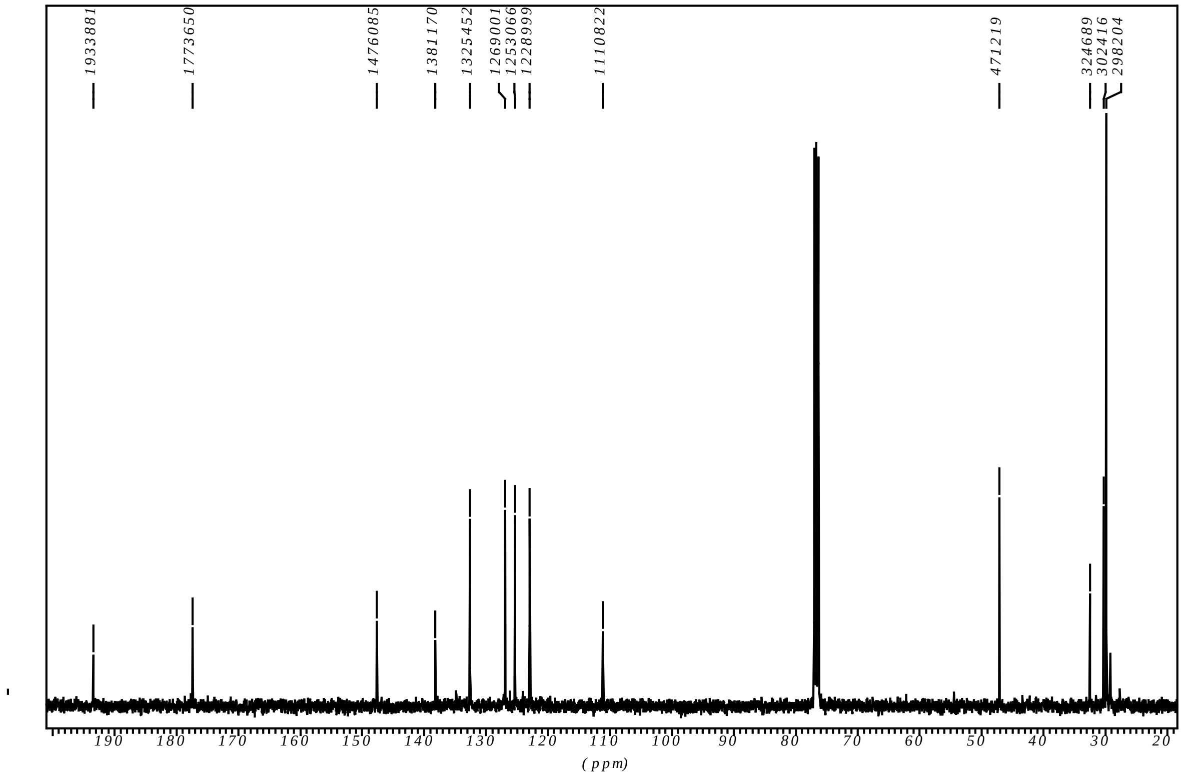

Figure S-12. ^{13}C NMR spectrum of 2-(3,3-dimethylbutyryl)-1-indanone **3d**.

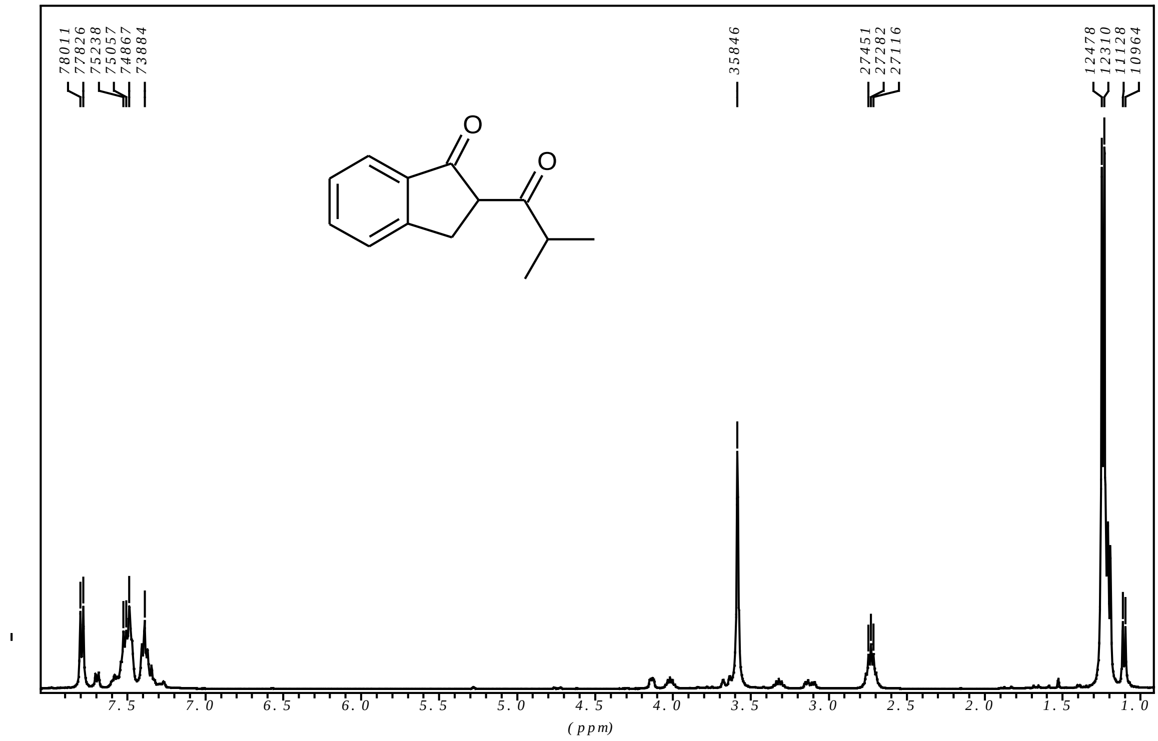

Figure S-13. ^{1}H NMR spectrum of 2-*iso*-butyryl-1-indanone **3e**.

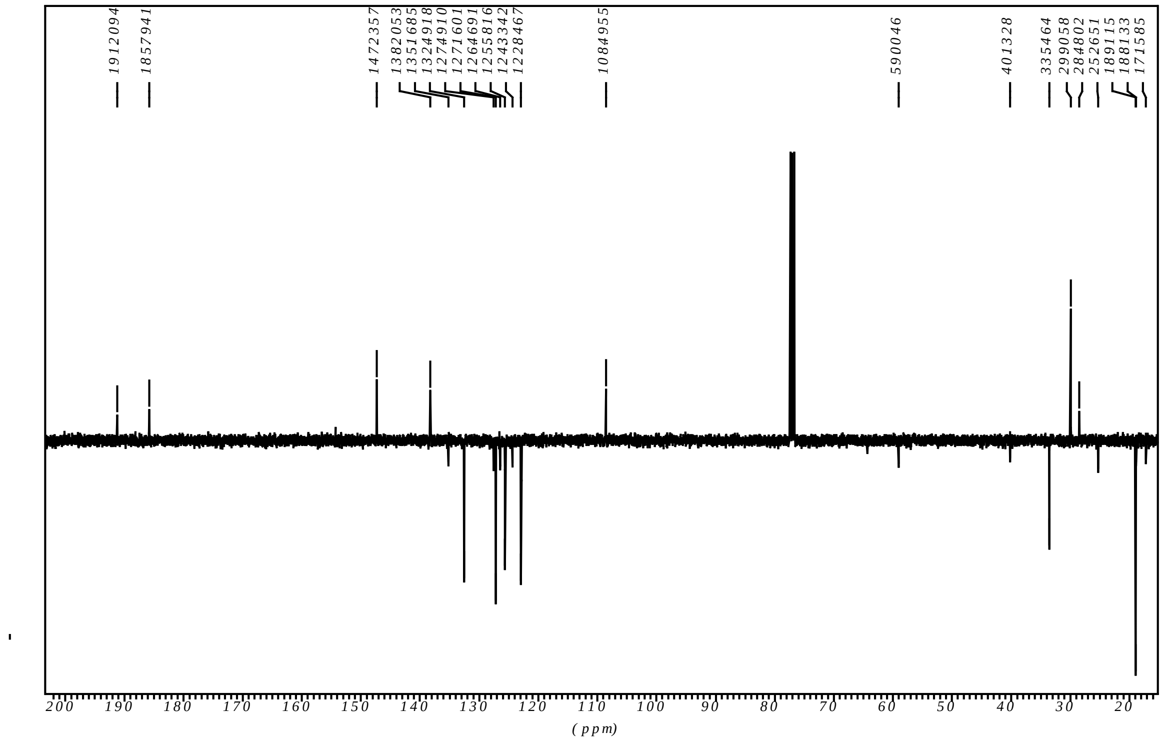

Figure S-14. ^{13}C NMR spectrum of 2-*iso*-butyryl-1-indanone **3e**.

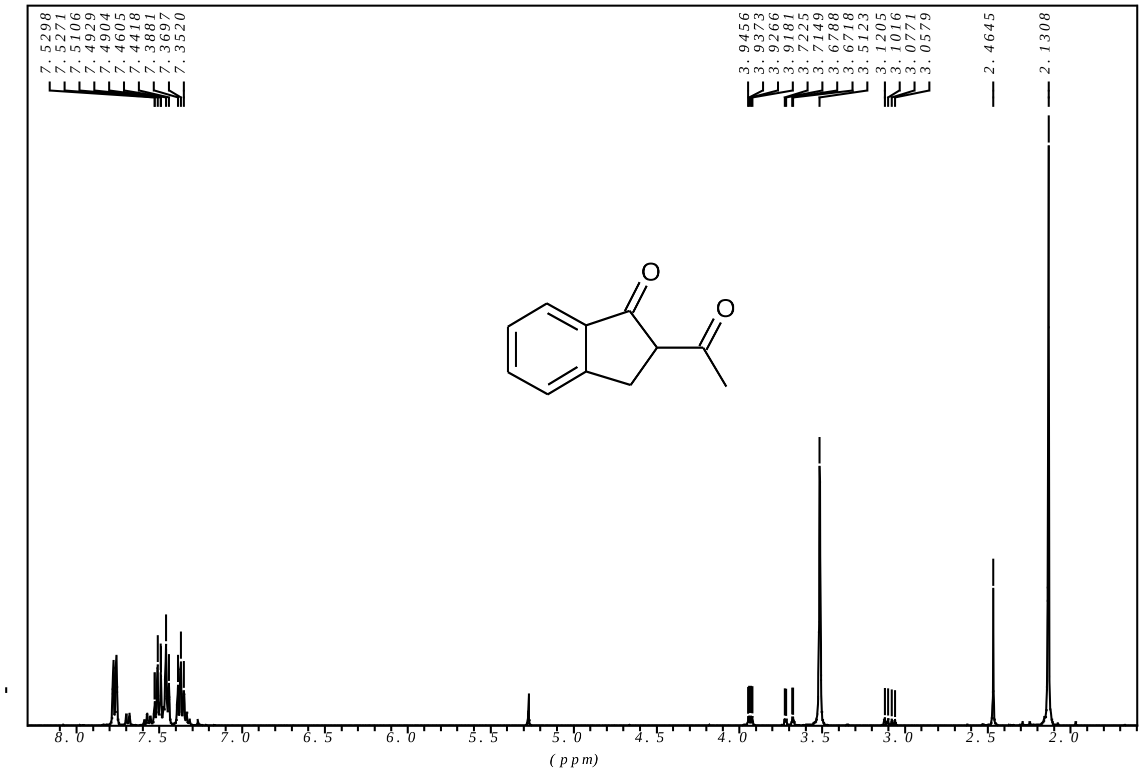

Figure S-15. ^{1}H NMR spectrum of 2-acetyl-1-indanone **3f**.

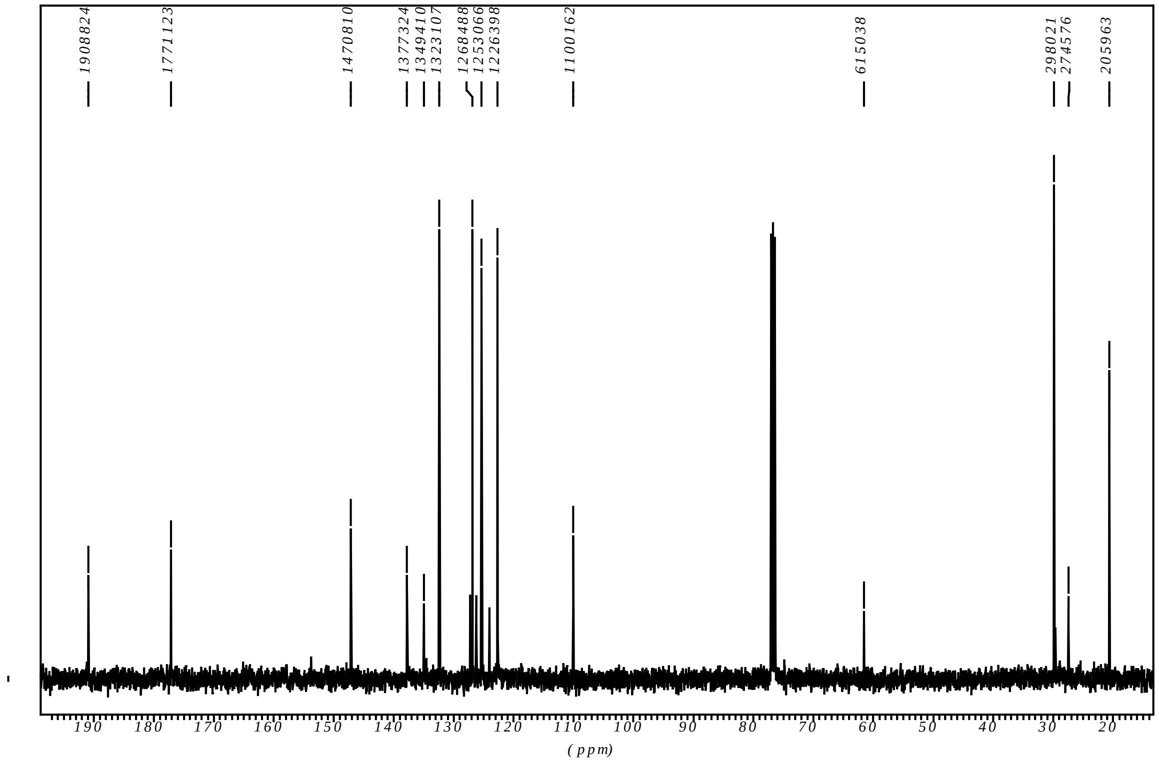

Figure S-16. ^{13}C NMR spectrum of 2-acetyl-1-indanone **3f**.

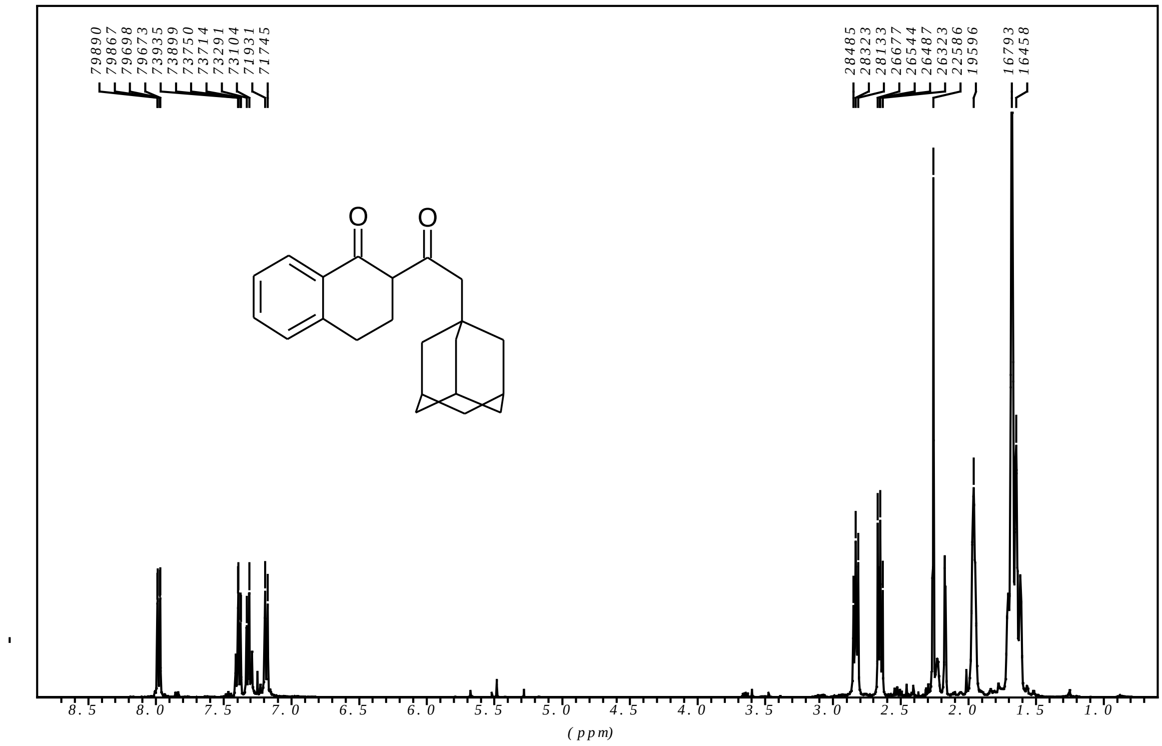

Figure S-17. ^{1}H NMR spectrum of 2-[2-(1-adamantnyl)acetyl]-1-tetralone **3h**.

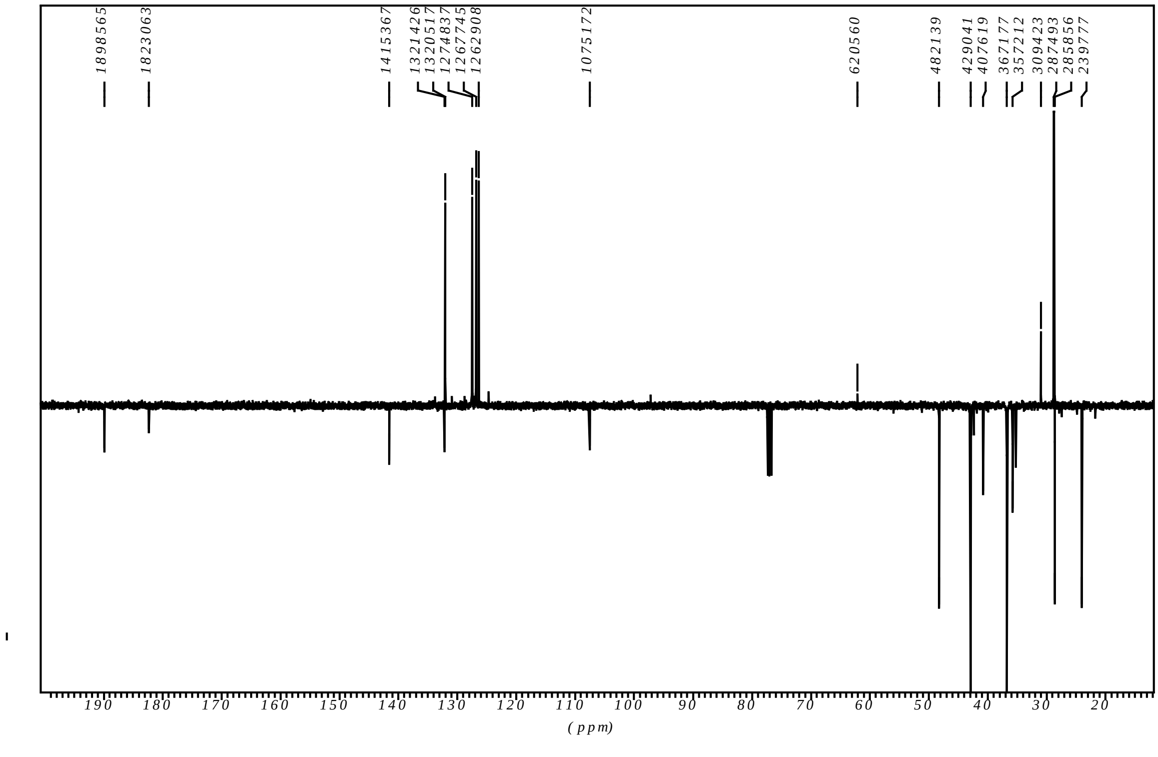

Figure S-18. ^{13}C NMR spectrum of 2-[2-(1-adamantnyl)acetyl]-1-tetralone **3h**.

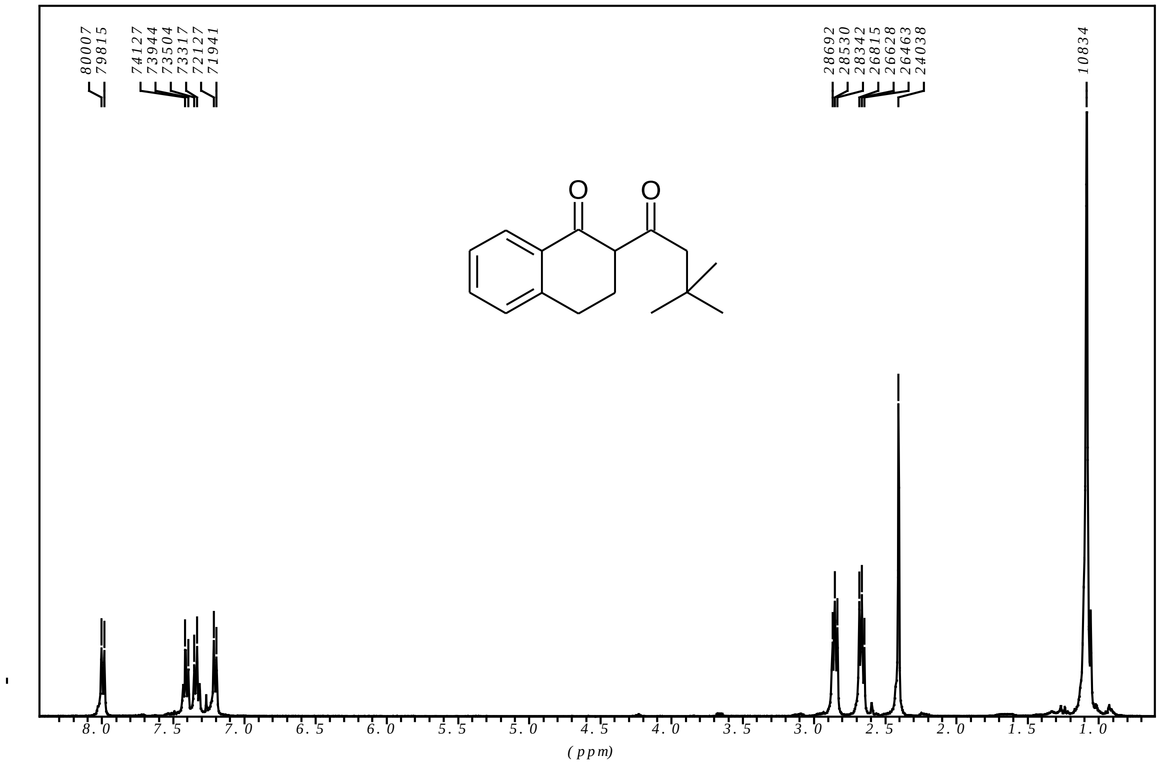

Figure S-19. ^{1}H NMR spectrum of 2-(3,3-dimethylbutyryl)-1-tetralone **3i**.

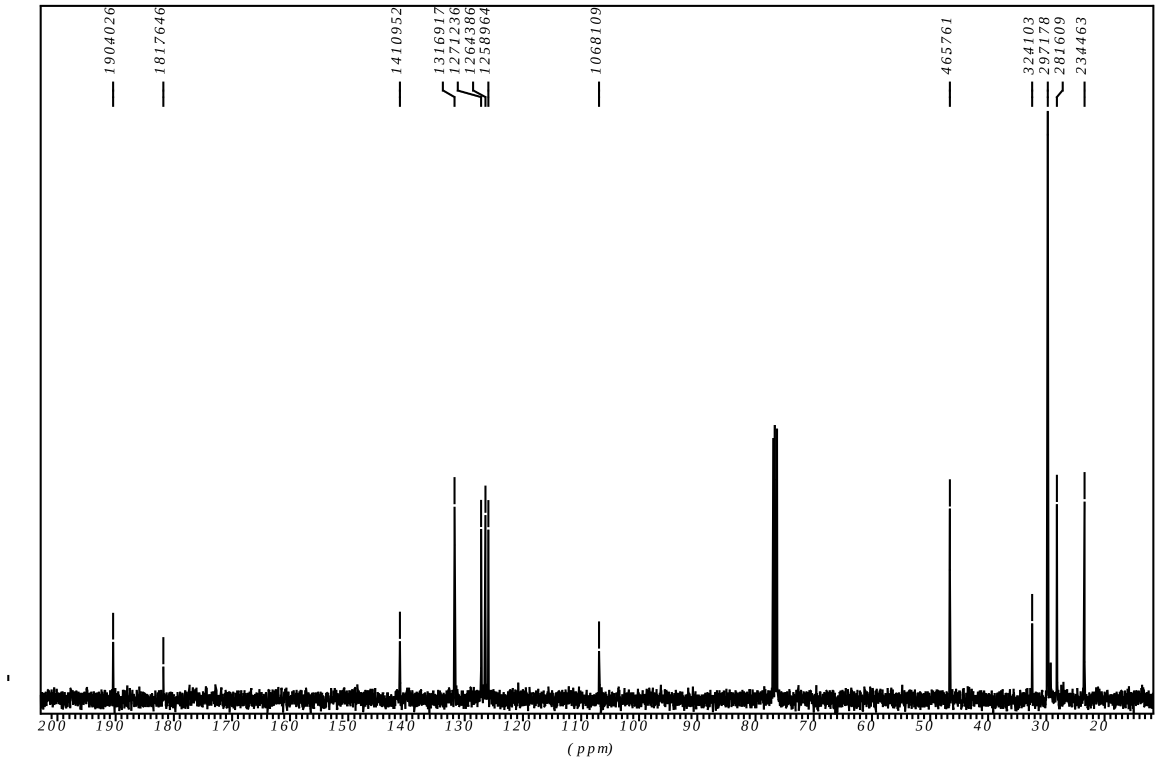

Figure S-20. ^{13}C NMR spectrum of 2-(3,3-dimethylbutyryl)-1-tetralone **3i**.

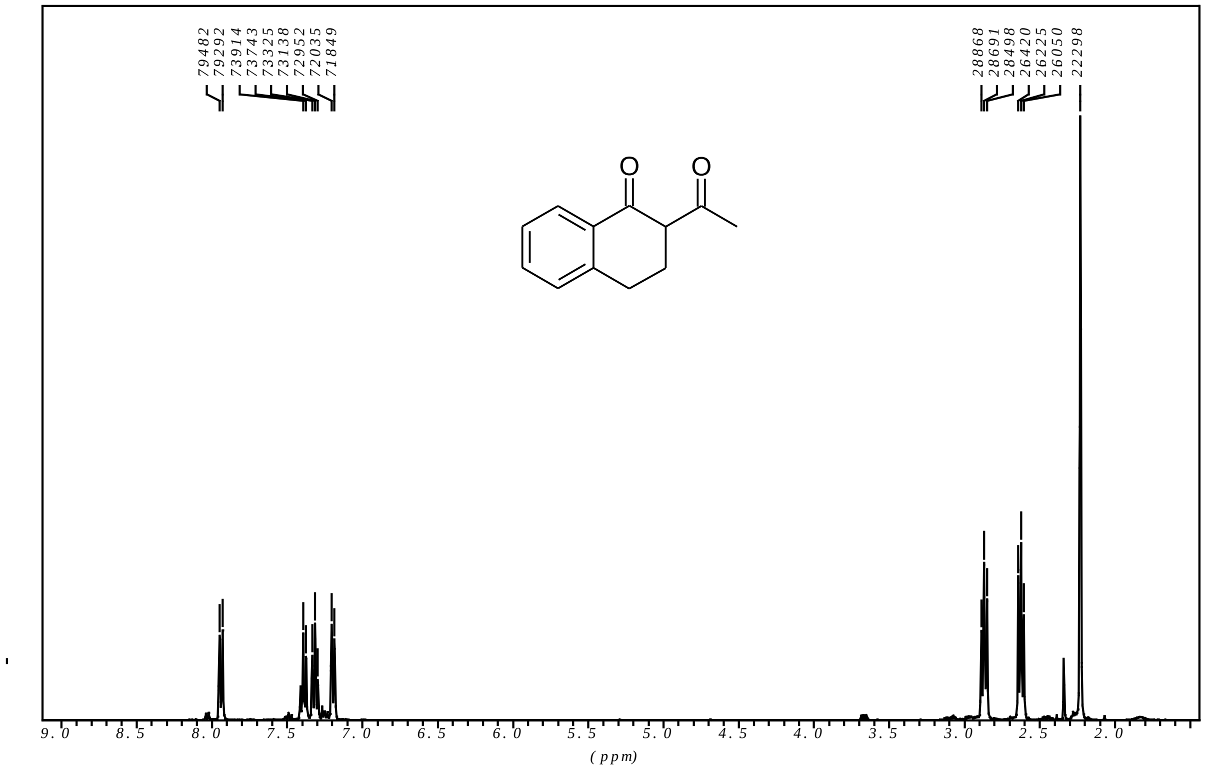

Figure S-21. ^{1}H NMR spectrum of 2-acetyl-1-tetralone **3j**.

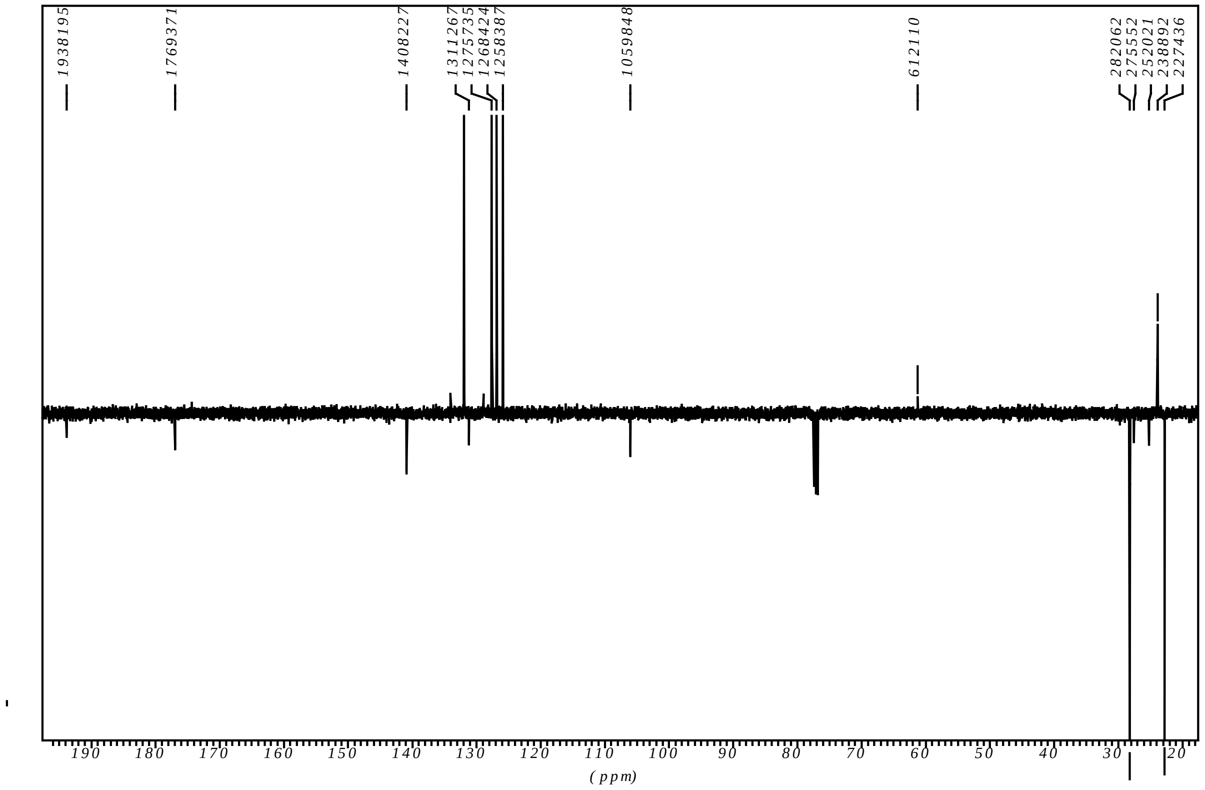

Figure S-22. ^{13}C NMR spectrum of 2-acetyl-1-tetralone **3j**.

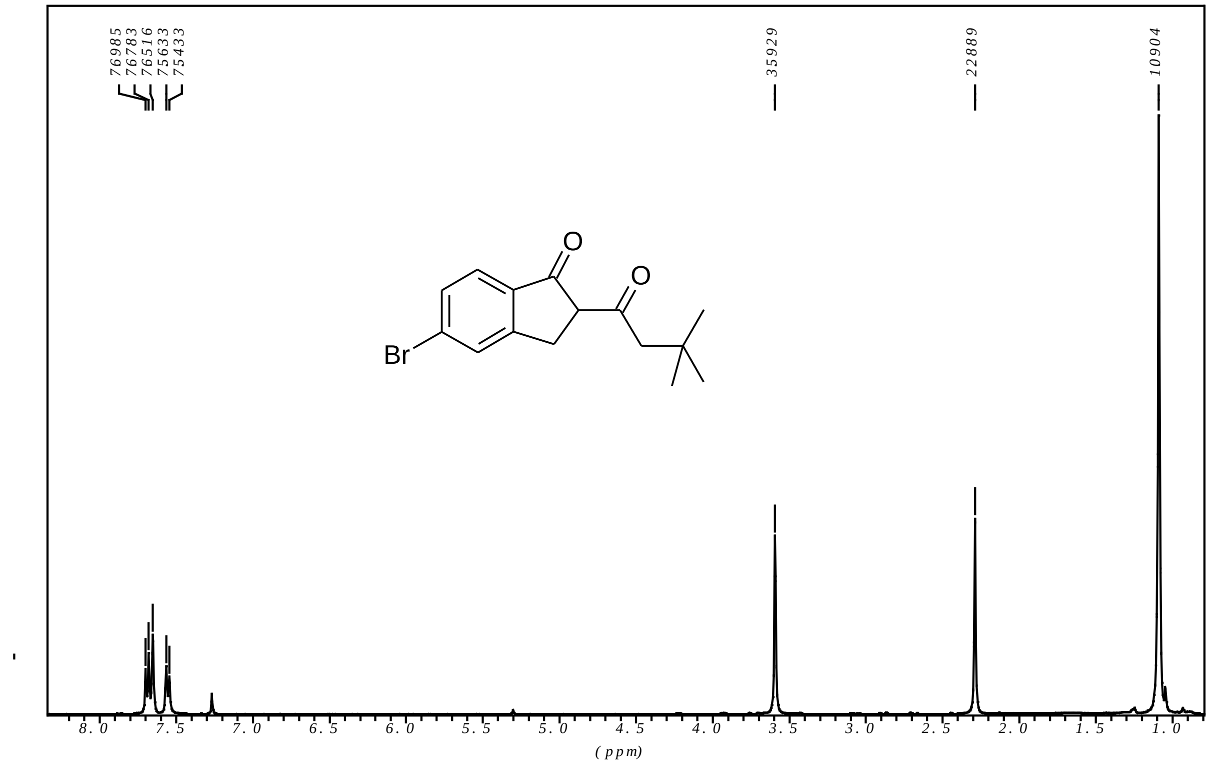

Figure S-23. ^{1}H NMR spectrum of 5-bromo-2-(3,3-dimethylbutyryl)-1-indanone **3k**.

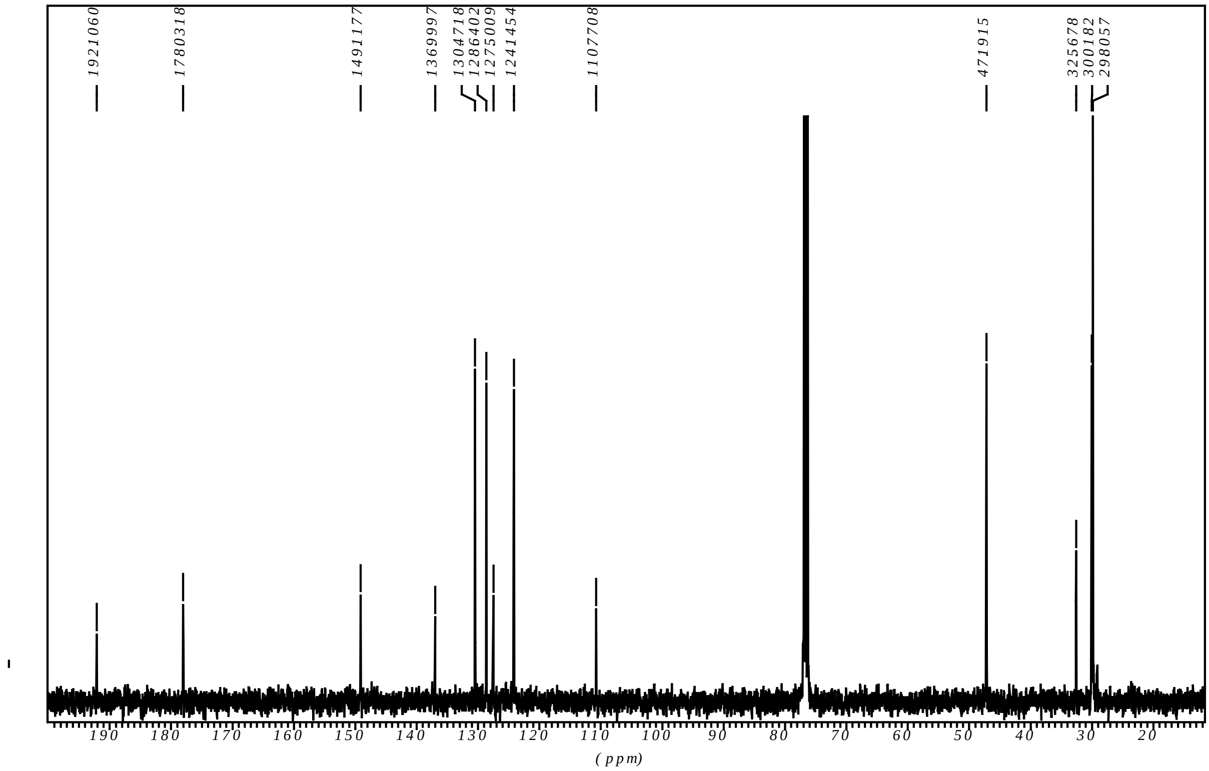

Figure S-24. ^{13}C NMR spectrum of 5-bromo-2-(3,3-dimethylbutyryl)-1-indanone **3k**.

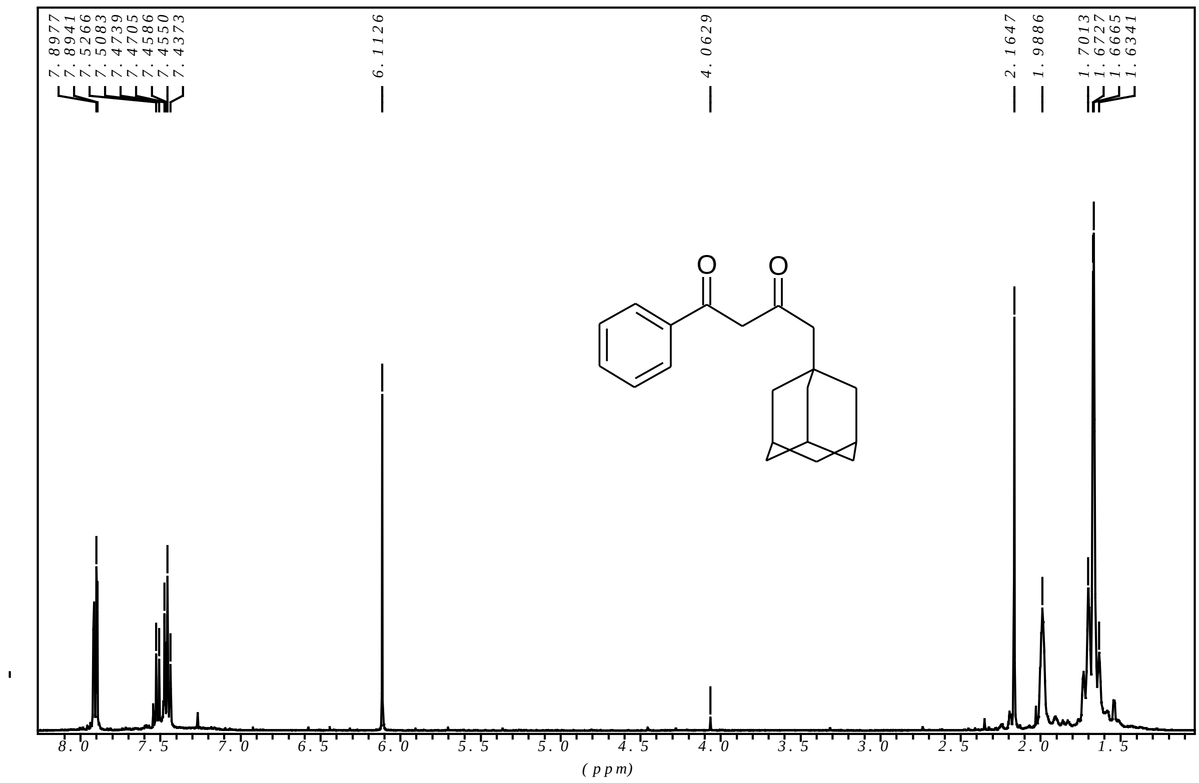

Figure S-25. ^{1}H NMR spectrum of 4-(1-adamantyl)-1-phenylbutane-1,3-dione **3l**.

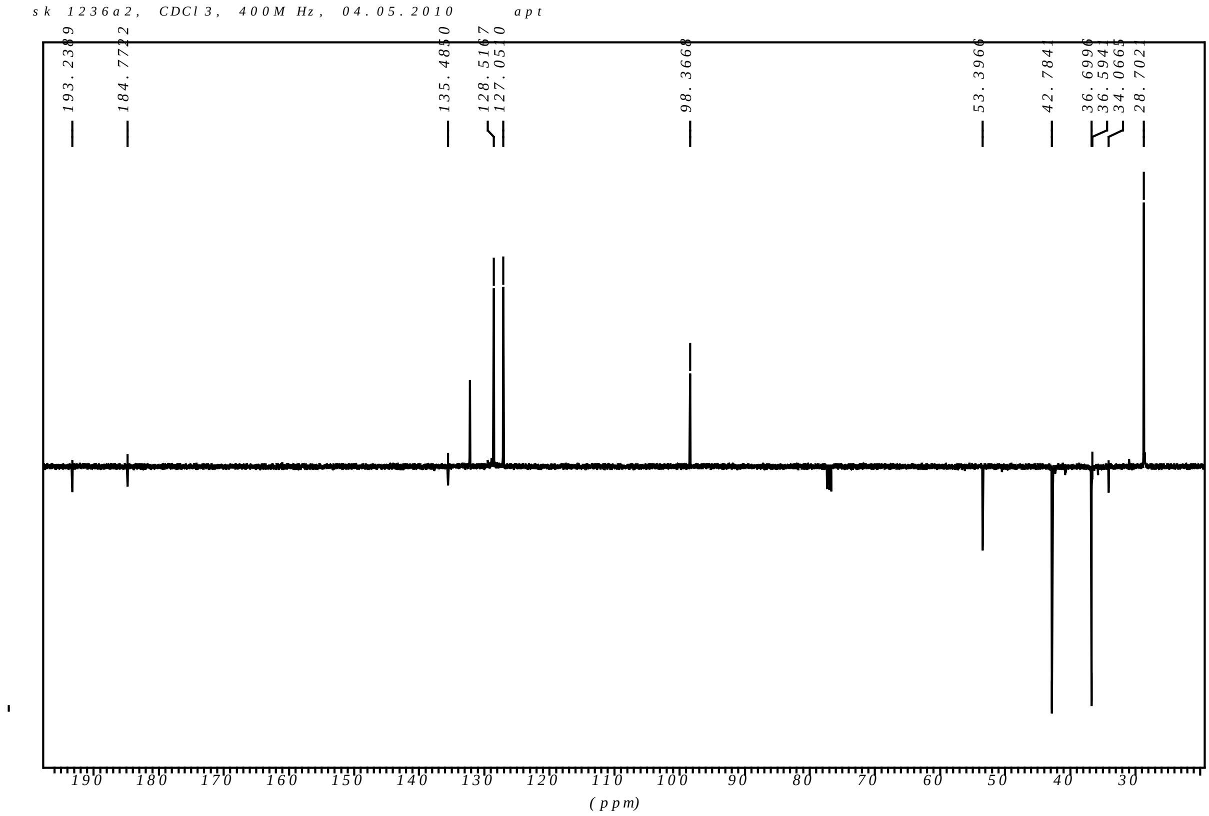

Figure S-26. ^{13}C NMR spectrum of 4-(1-adamantyl)-1-phenylbutane-1,3-dione **3l**.

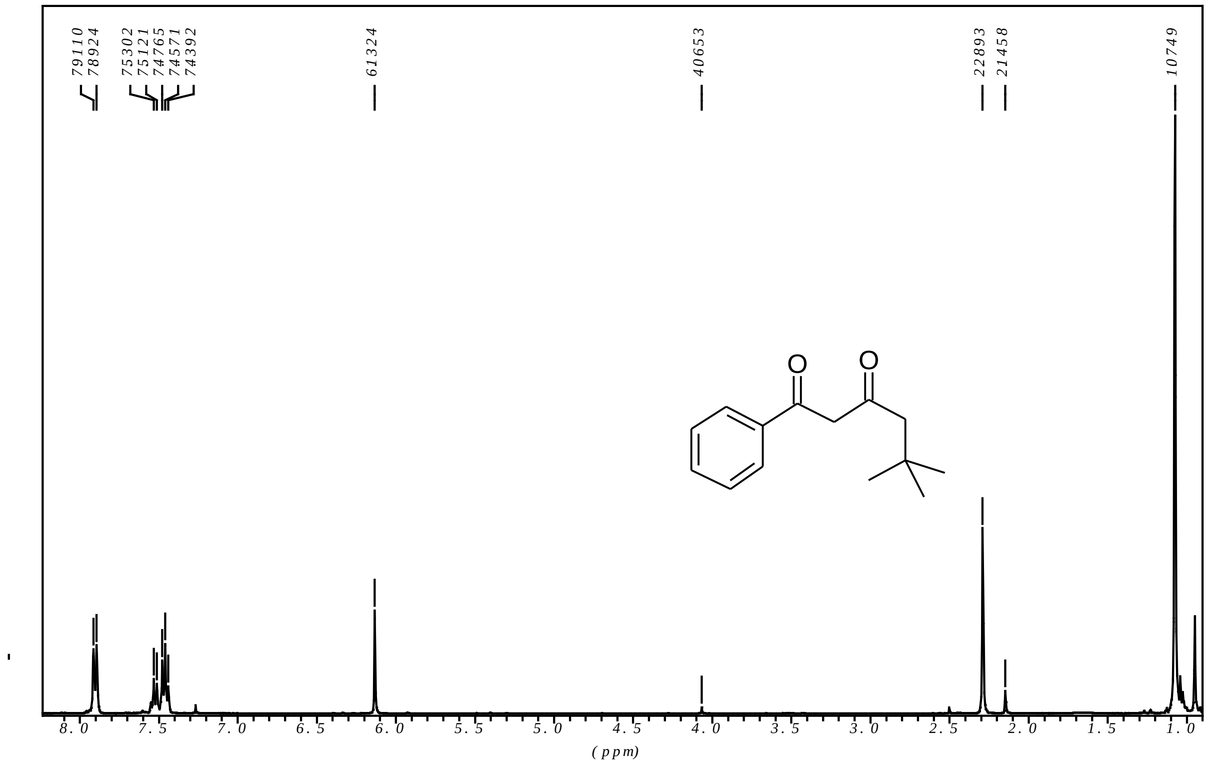

Figure S-27. ^{1}H NMR spectrum of 5,5-dimethyl-1-phenylhexane-1,3-dione **3m**.

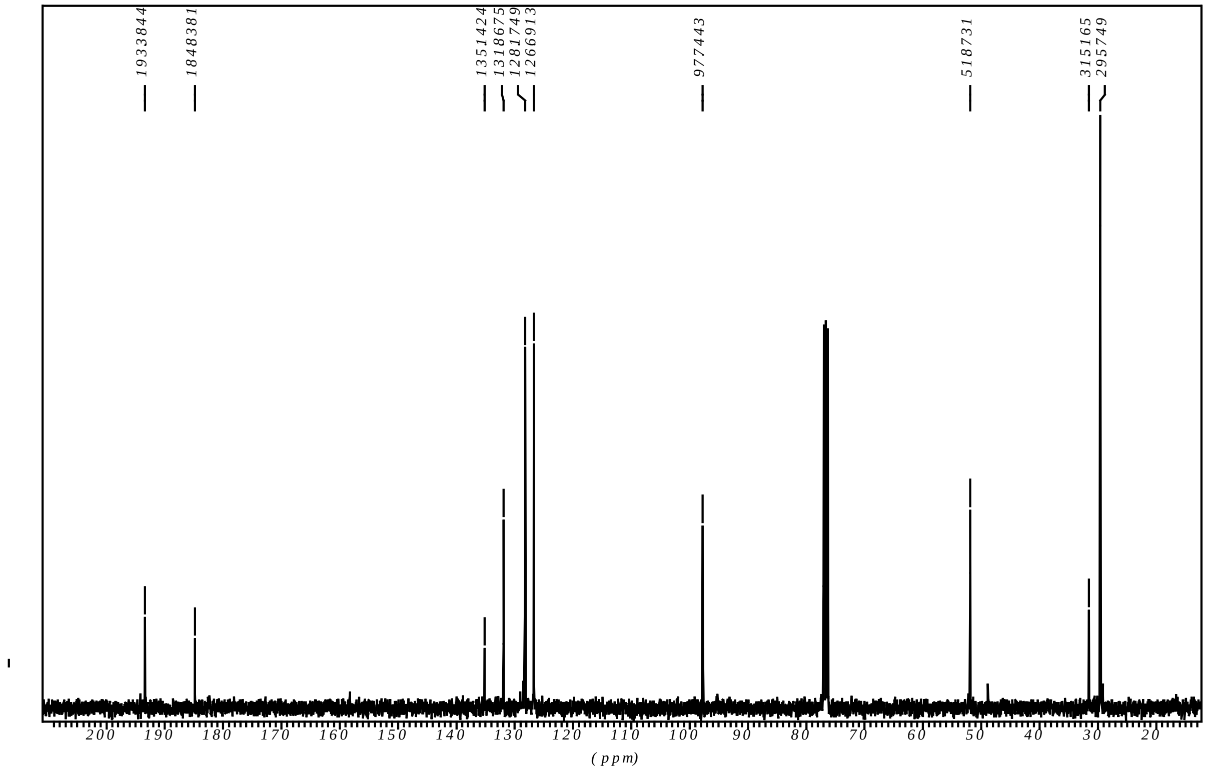

Figure S-28. ^{13}C NMR spectrum of 5,5-dimethyl-1-phenylhexane-1,3-dione **3m**.

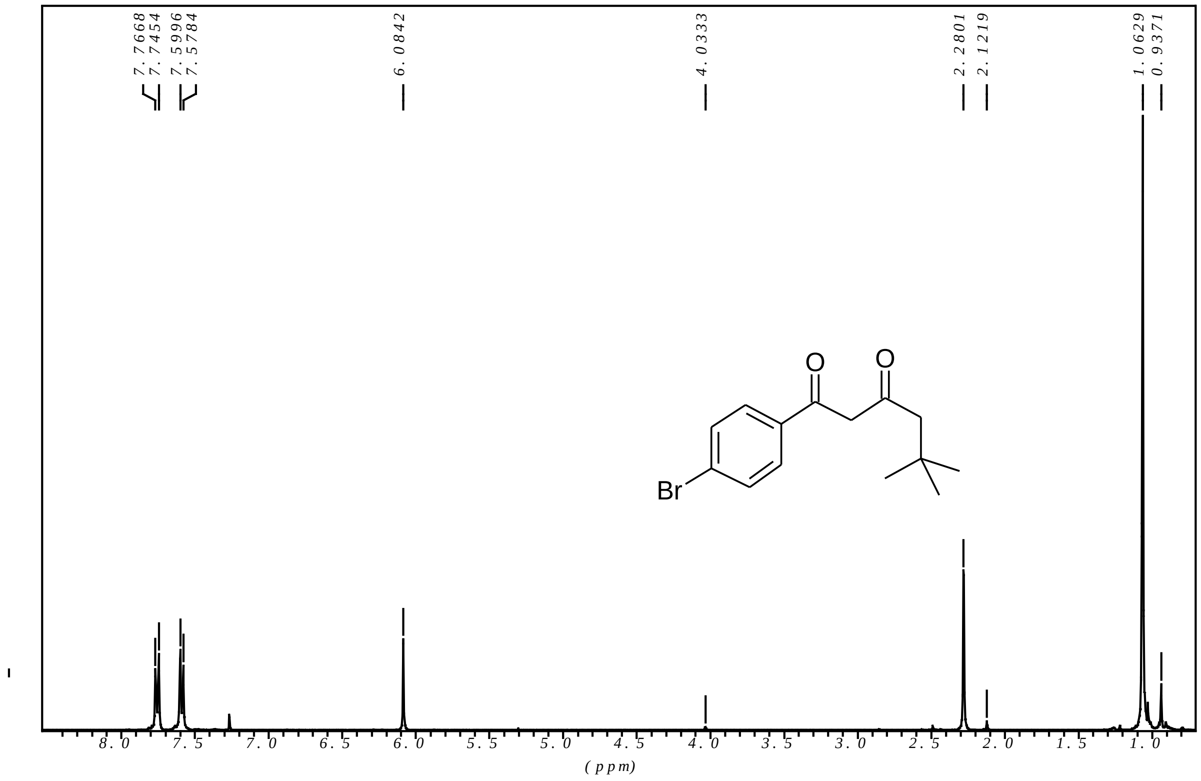

Figure S-29. ^{1}H NMR spectrum of 1-(4-bromophenyl)-5,5-dimethylhexane-1,3-dione **3n**.

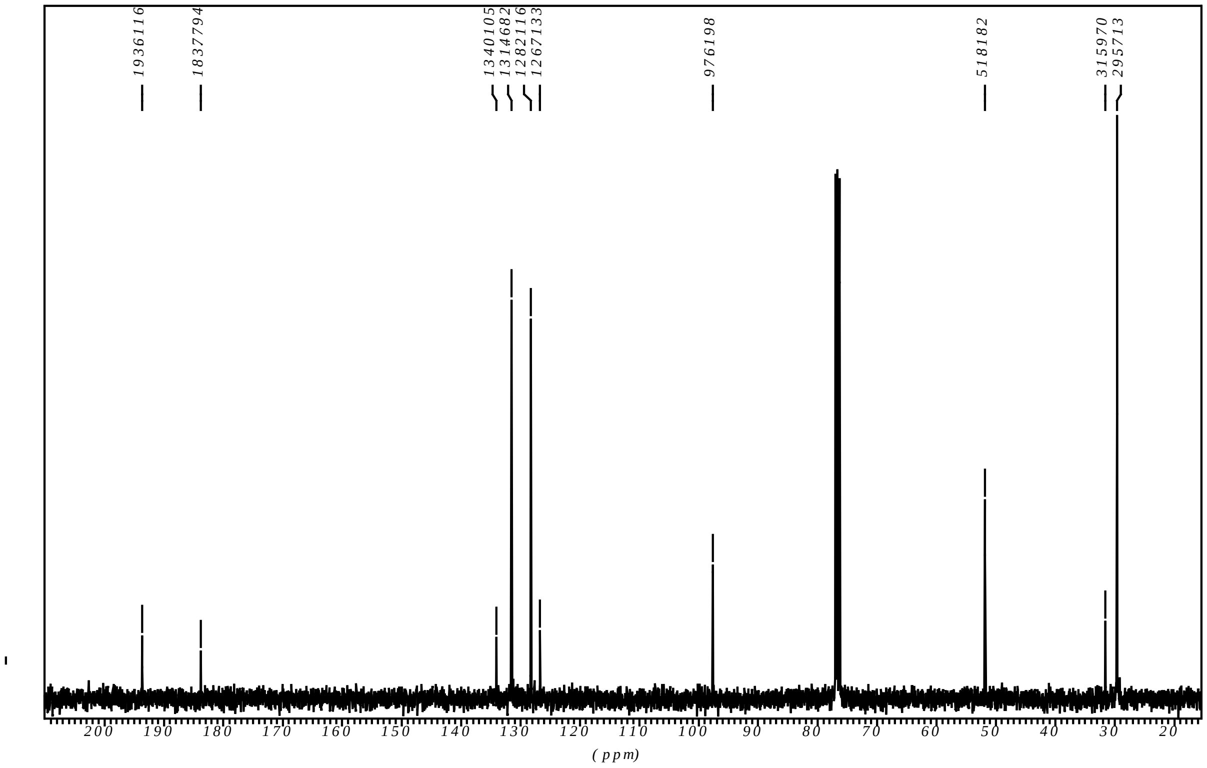

Figure S-30. ^{13}C NMR spectrum of 1-(4-bromophenyl)-5,5-dimethylhexane-1,3-dione **3n**.

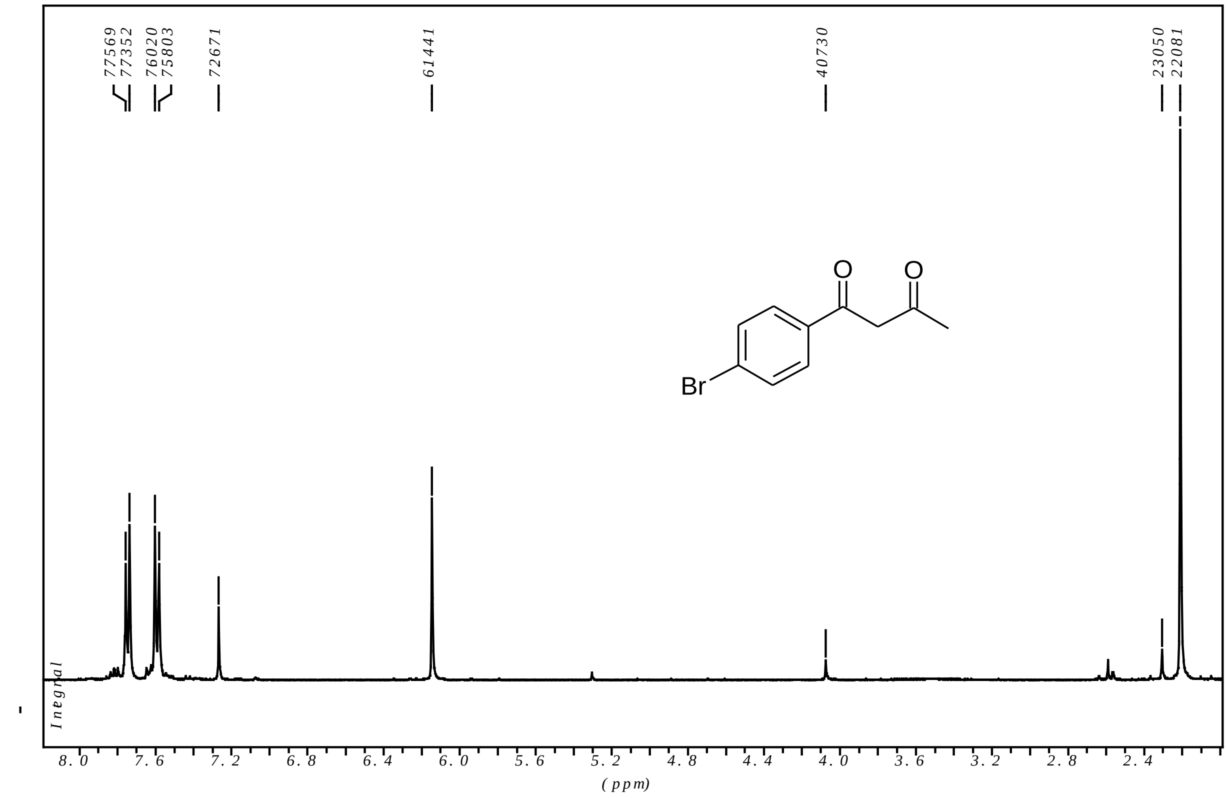

Figure S-31. ^{1}H NMR spectrum of 1-(4-bromophenyl)butane-1,3-dione **3o**.

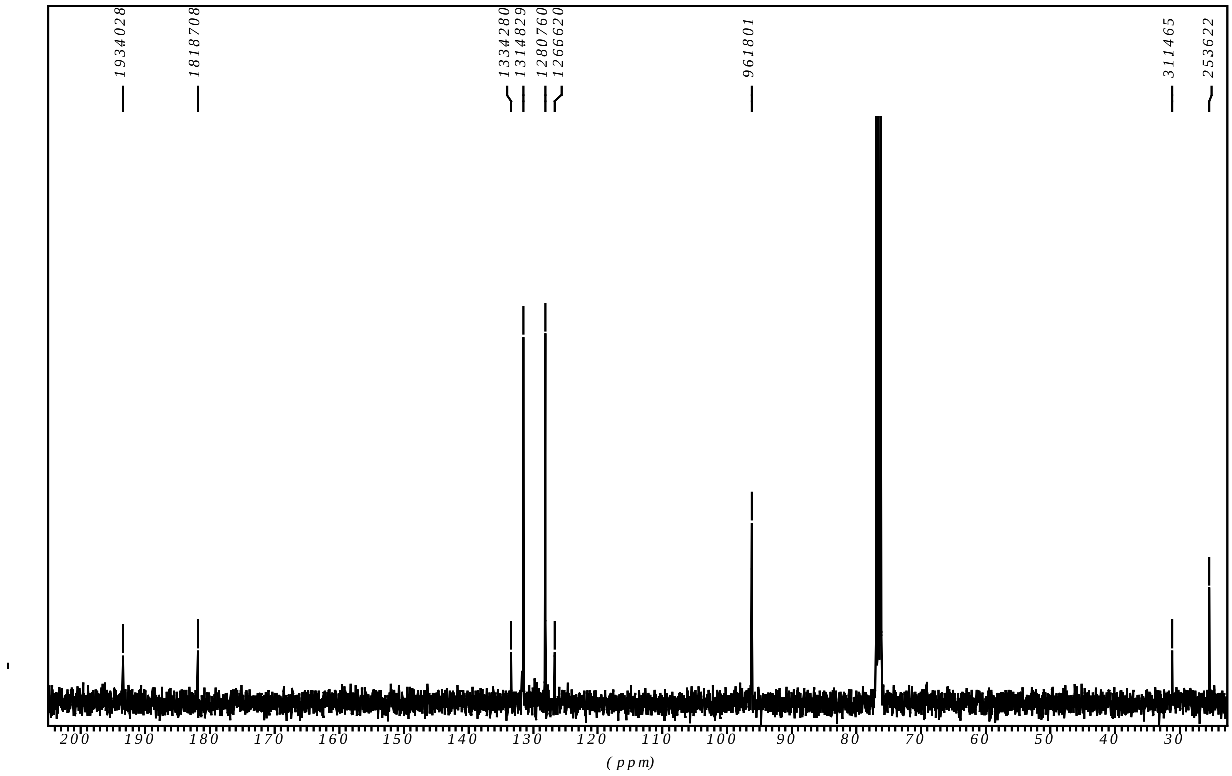

Figure S-32. ^{13}C NMR spectrum of 1-(4-bromophenyl)butane-1,3-dione **3o**.

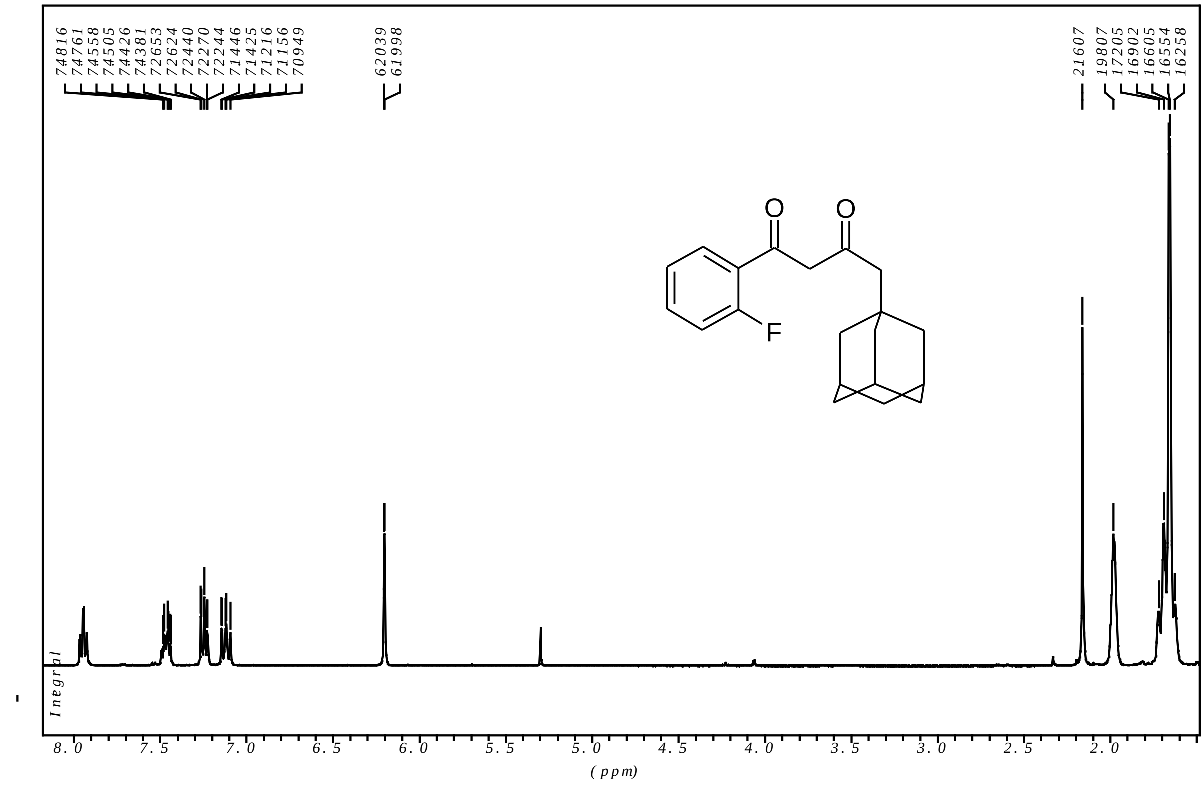

Figure S-33. ^{1}H NMR spectrum of 4-(1-adamantyl)-1-(2-fluorophenyl)butane-1,3-dione **3p**.

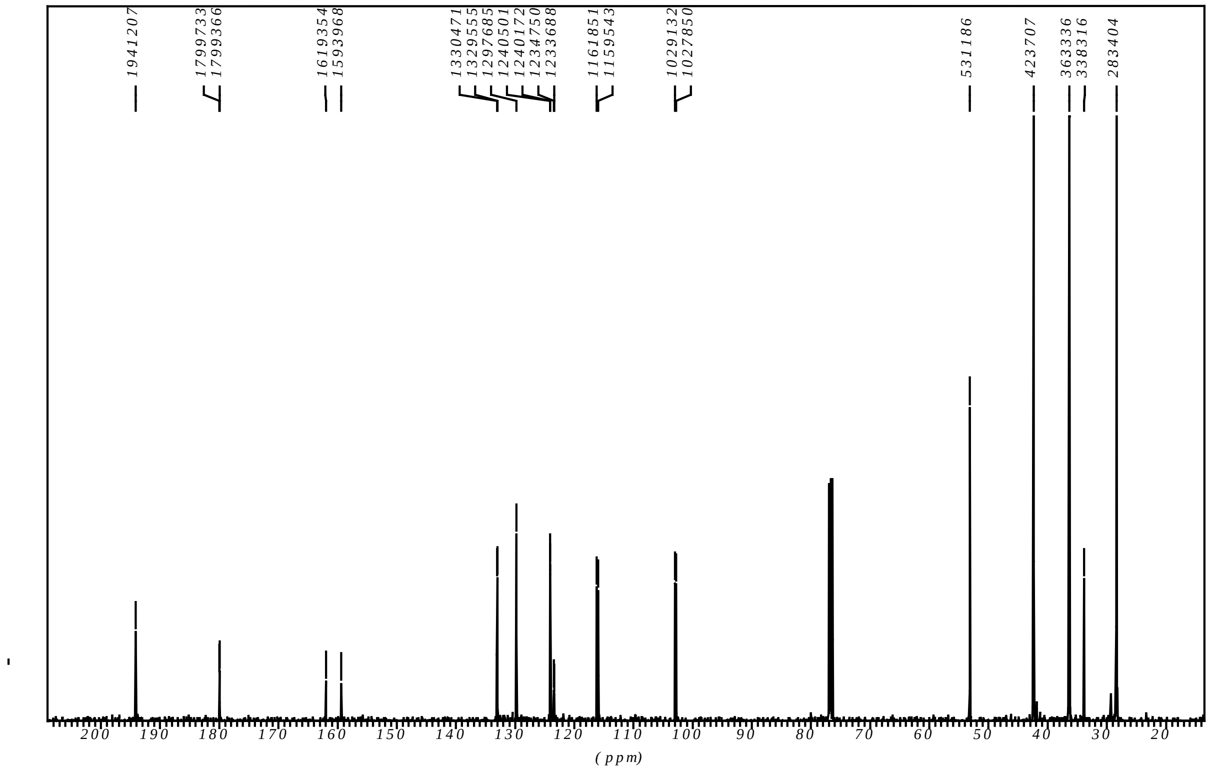

Figure S-34. ^{13}C NMR spectrum of 4-(1-adamantyl)-1-(2-fluorophenyl)butane-1,3-dione **3p**.

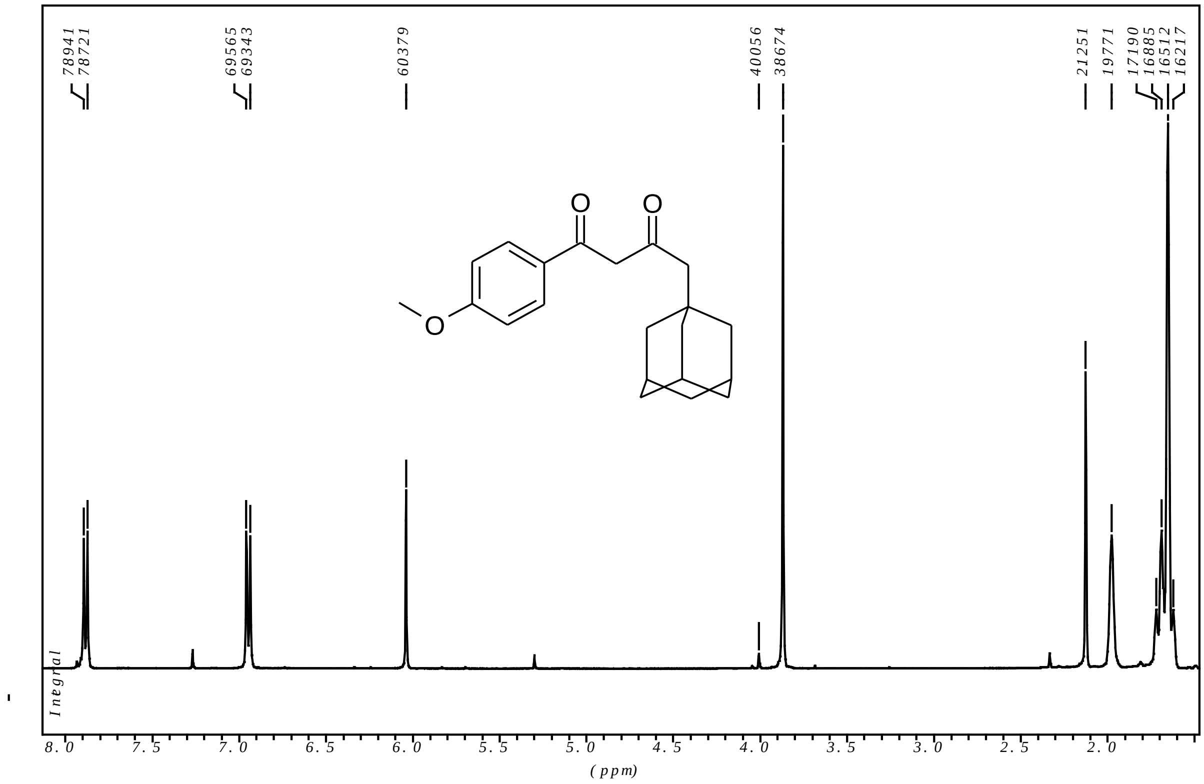

Figure S-35. ^{1}H NMR spectrum of 4-(1-adamantyl)-1-(4-methoxyphenyl)butane-1,3-dione **3q**.

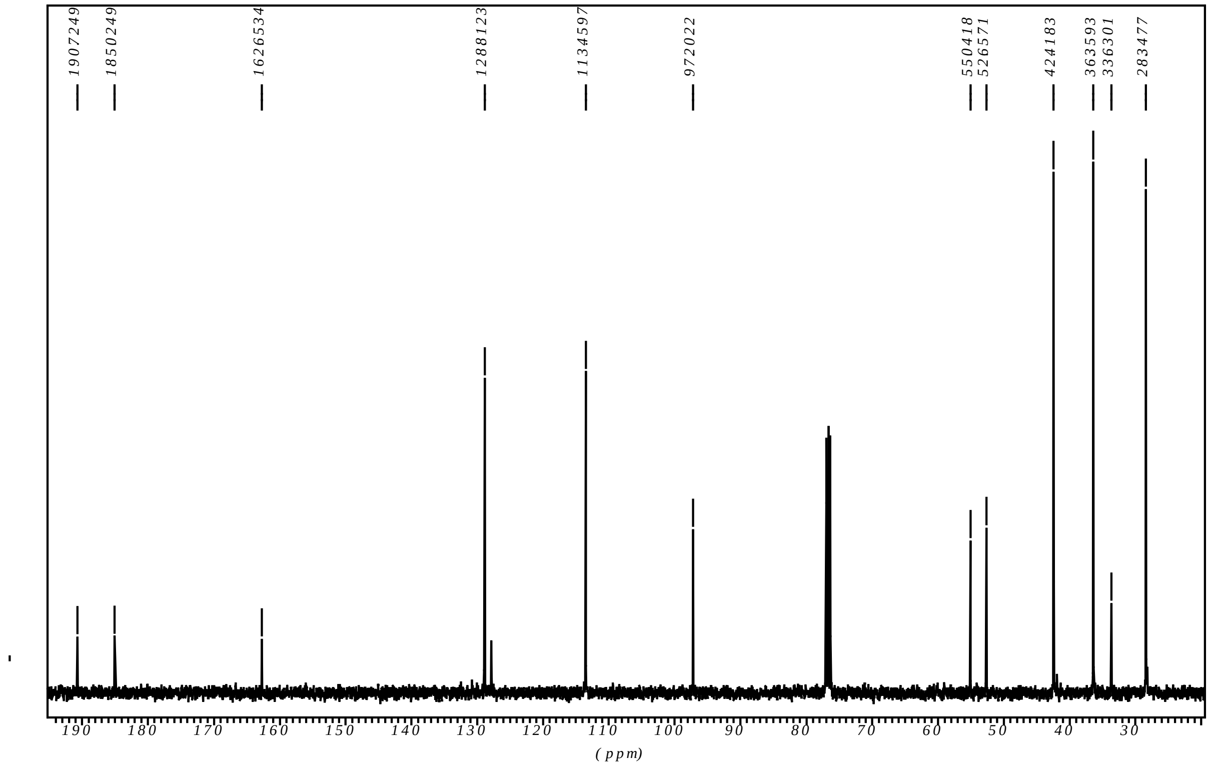

Figure S-36. ^{13}C NMR spectrum of 4-(1-adamantyl)-1-(4-methoxyphenyl)butane-1,3-dione **3q**.

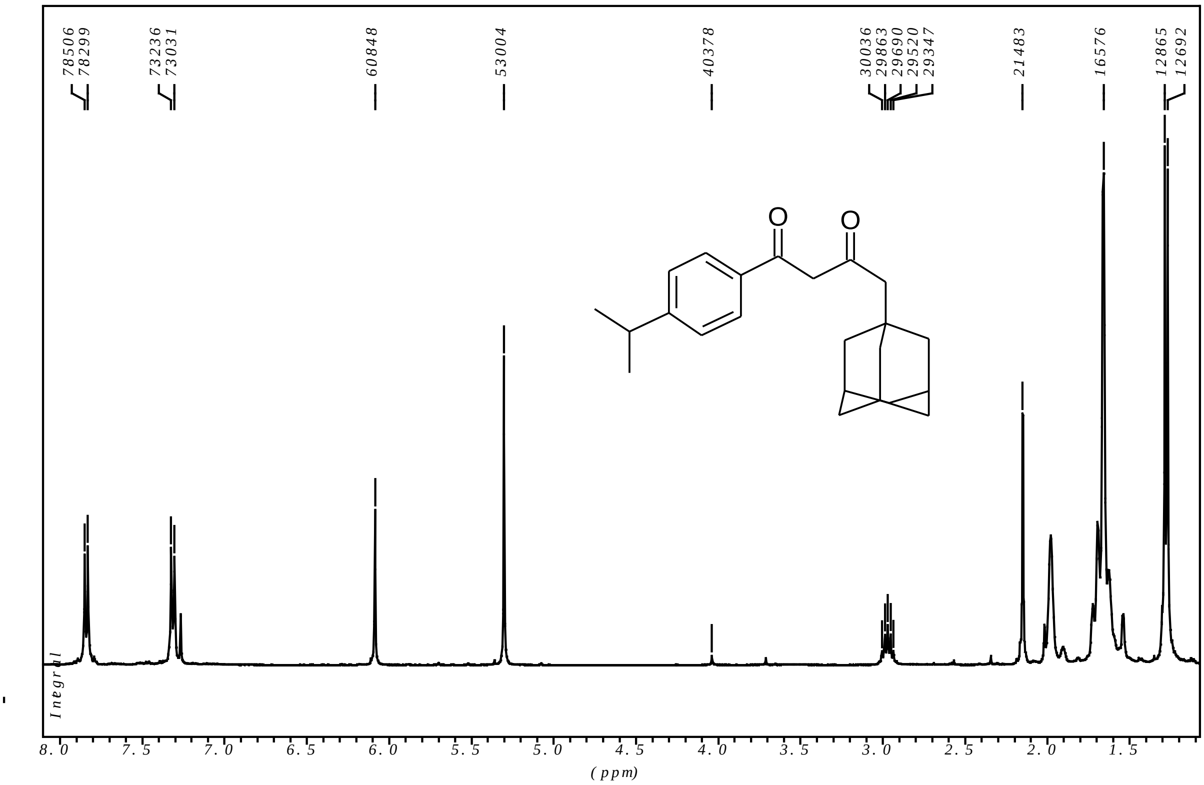

Figure S-37. ^{1}H NMR spectrum of 4-(1-adamantyl)-1-(4-*iso*-propylphenyl)butane-1,3-dione **3r**.

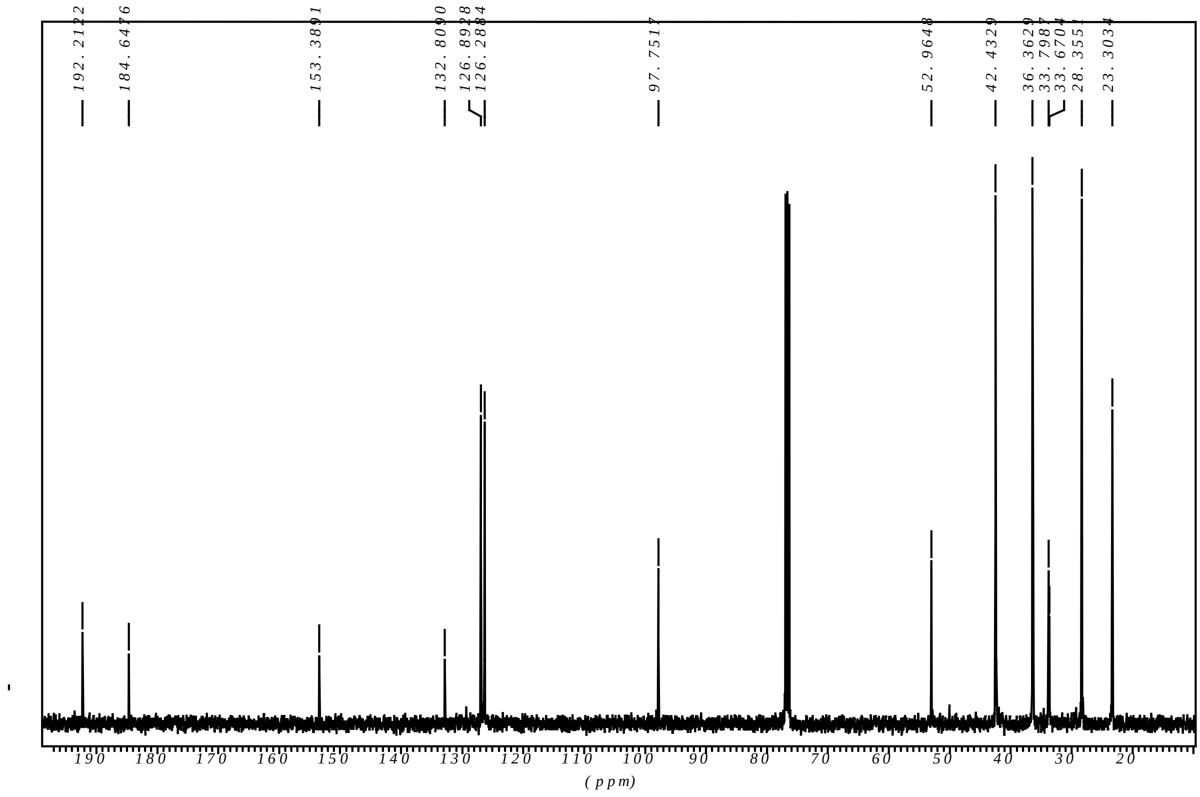

Figure S-38. ^{13}C NMR spectrum of 4-(1-adamantyl)-1-(4-*iso* propylphenyl)butane-1,3-dione **3r**.

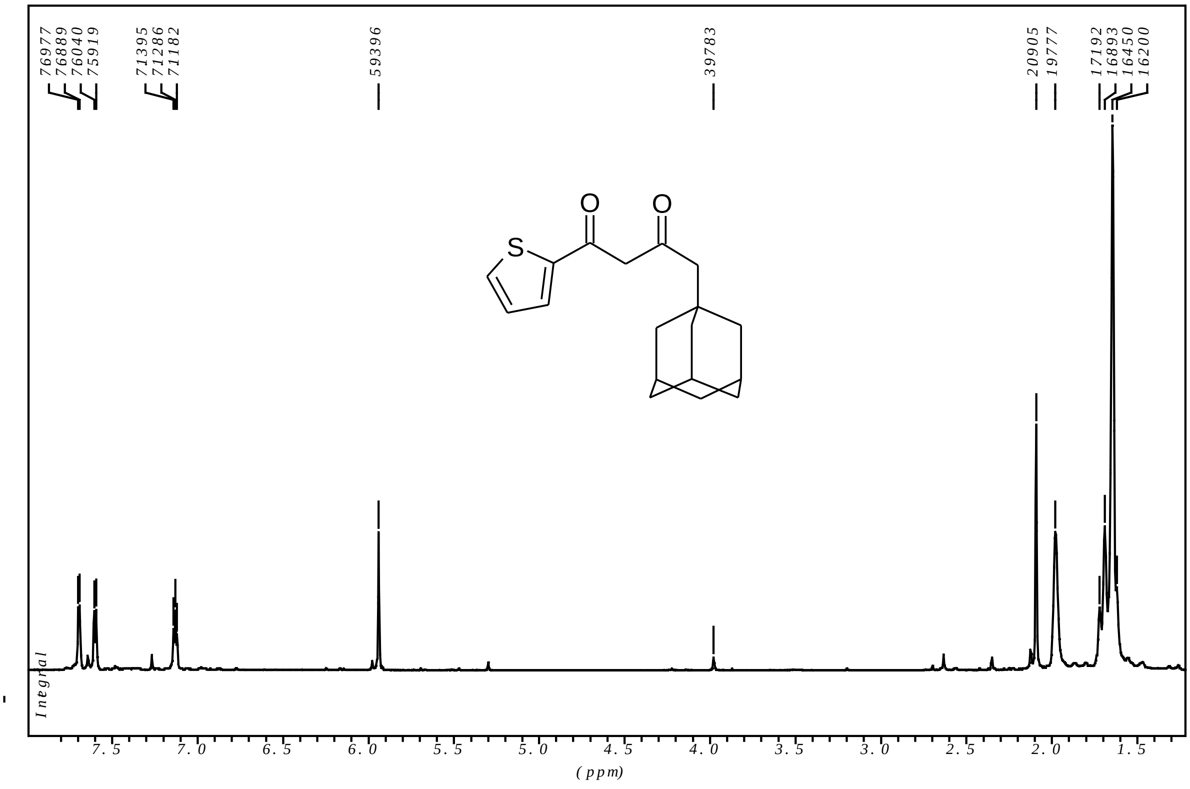

Figure S-39. ^{1}H NMR spectrum of 4-(1-adamantyl)-1-(2-thienyl)butane-1,3-dione **3s**.

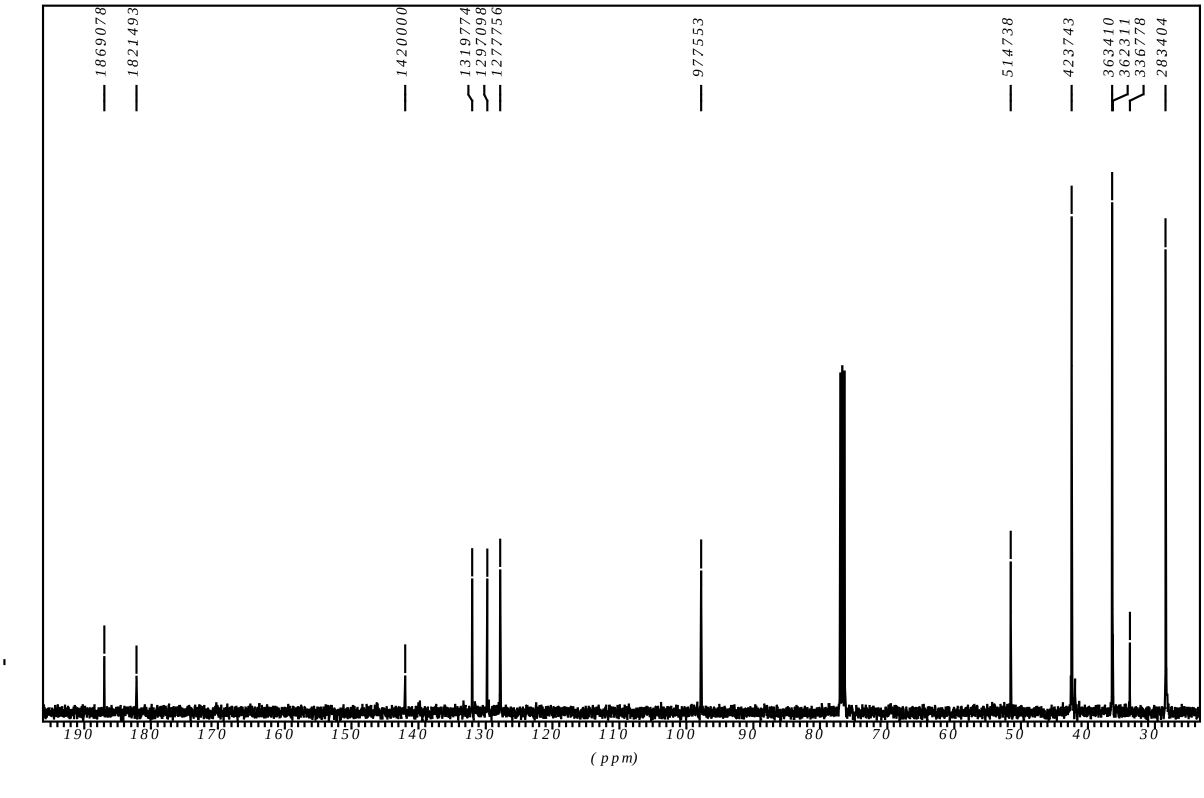

Figure S-40. ^{13}C NMR spectrum of 4-(1-adamantyl)-1-(2-thienyl)butane-1,3-dione **3s**.

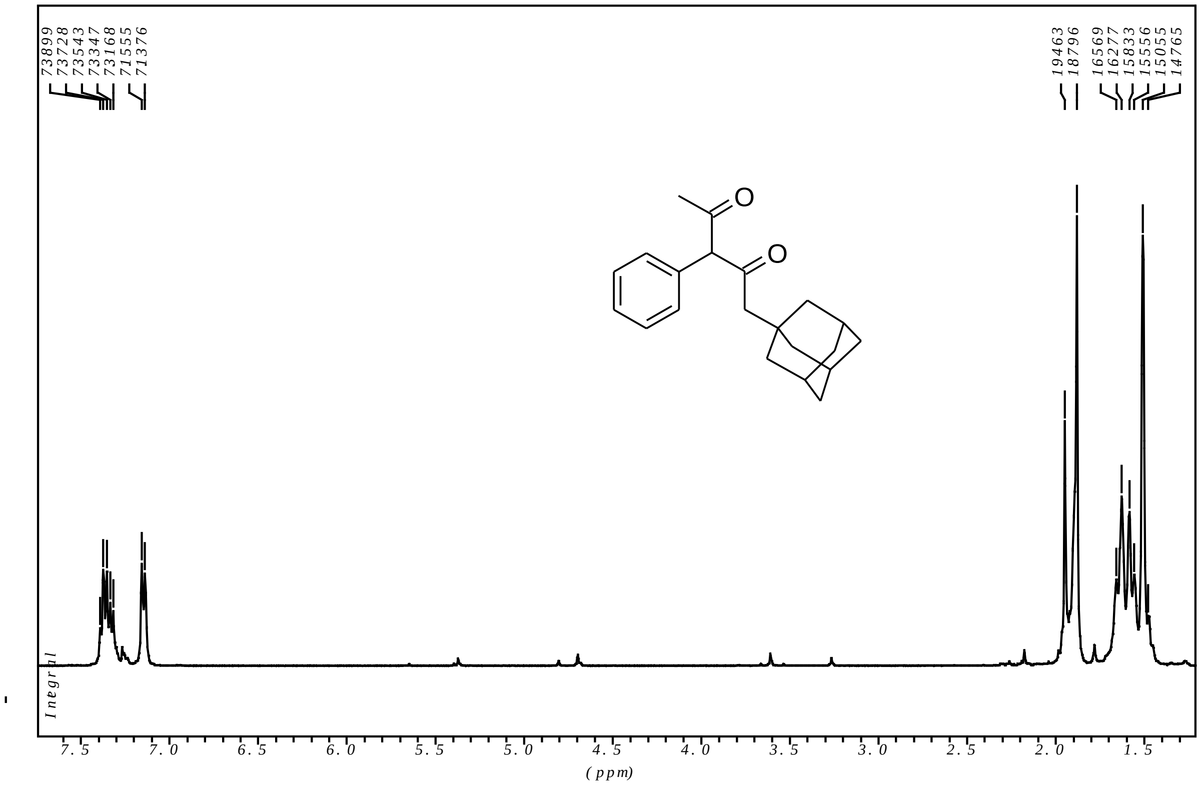

Figure S-41. ^{1}H NMR spectrum of 1-(1-adamantyl)-3-phenylpentane-2,4-dione **3t**.

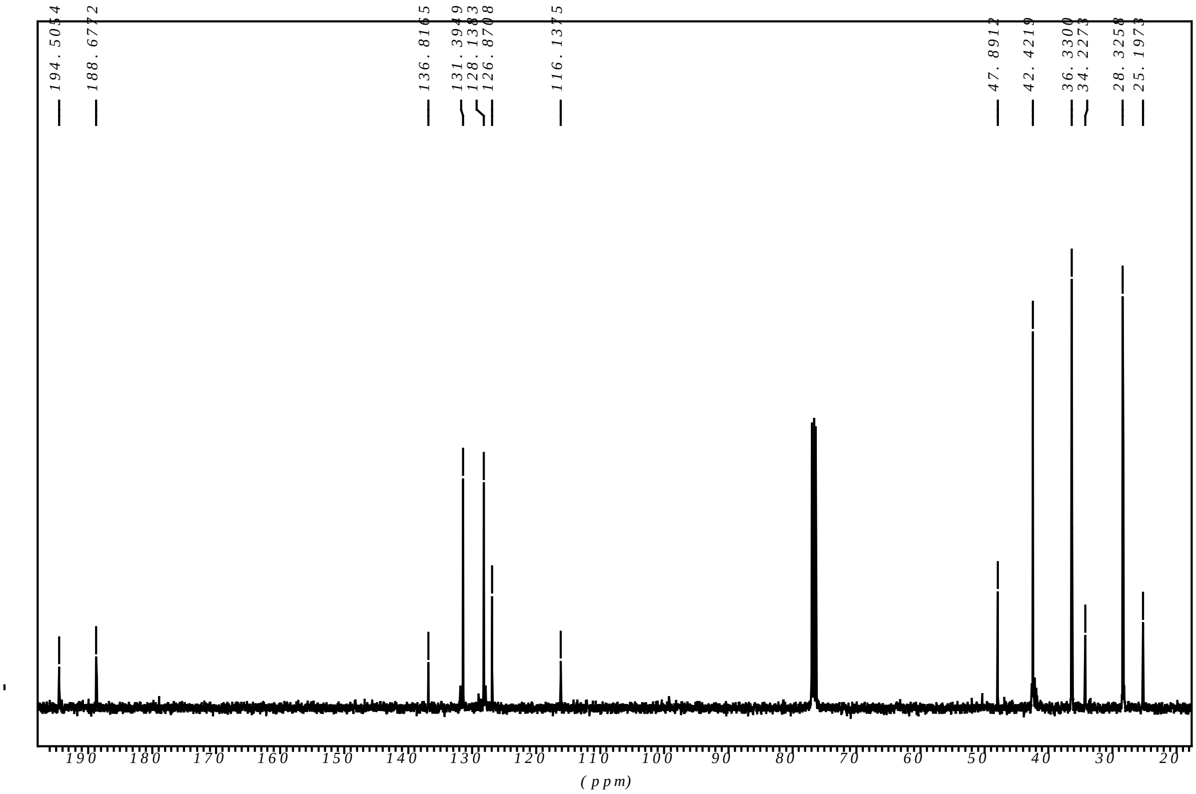

Figure S-42. ^{13}C NMR spectrum of 1-(1-adamantyl)-3-phenylpentane-2,4-dione **3t**.

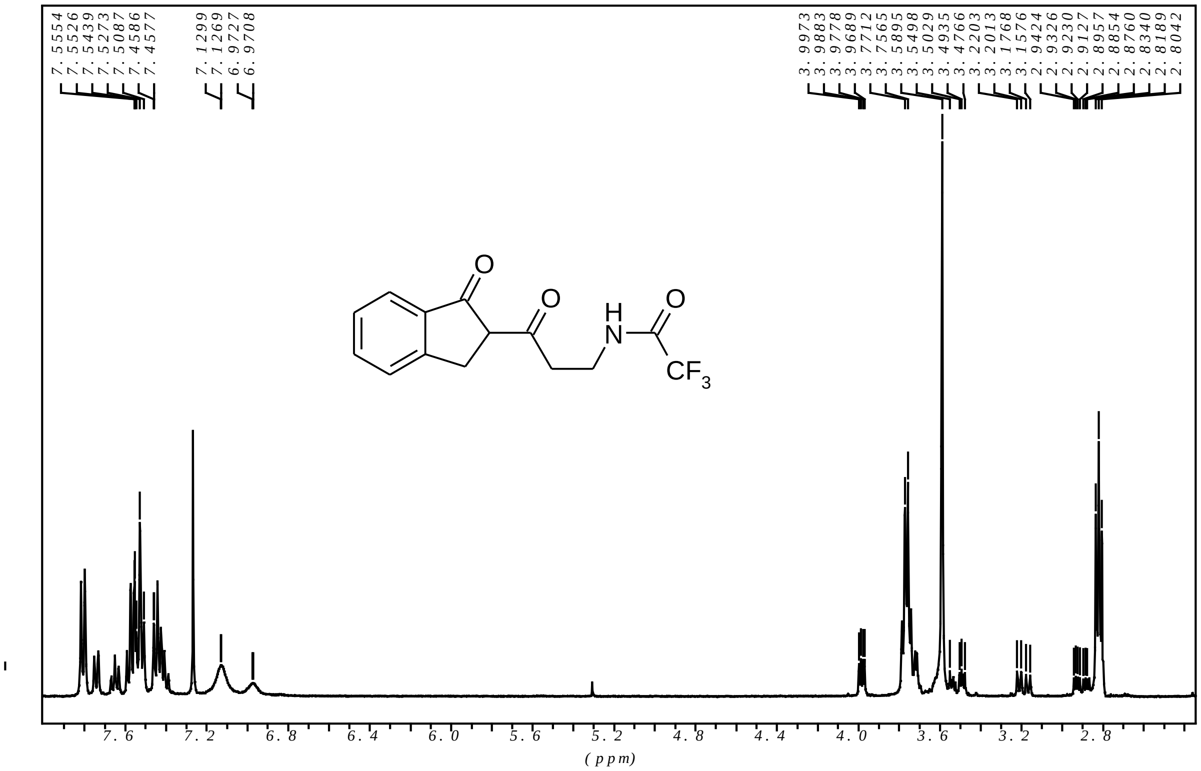

Figure S-43. ^{1}H NMR spectrum of 2-[3-(*N*-trifluroacetylamino)propionyl]-1-indanone **3u**.

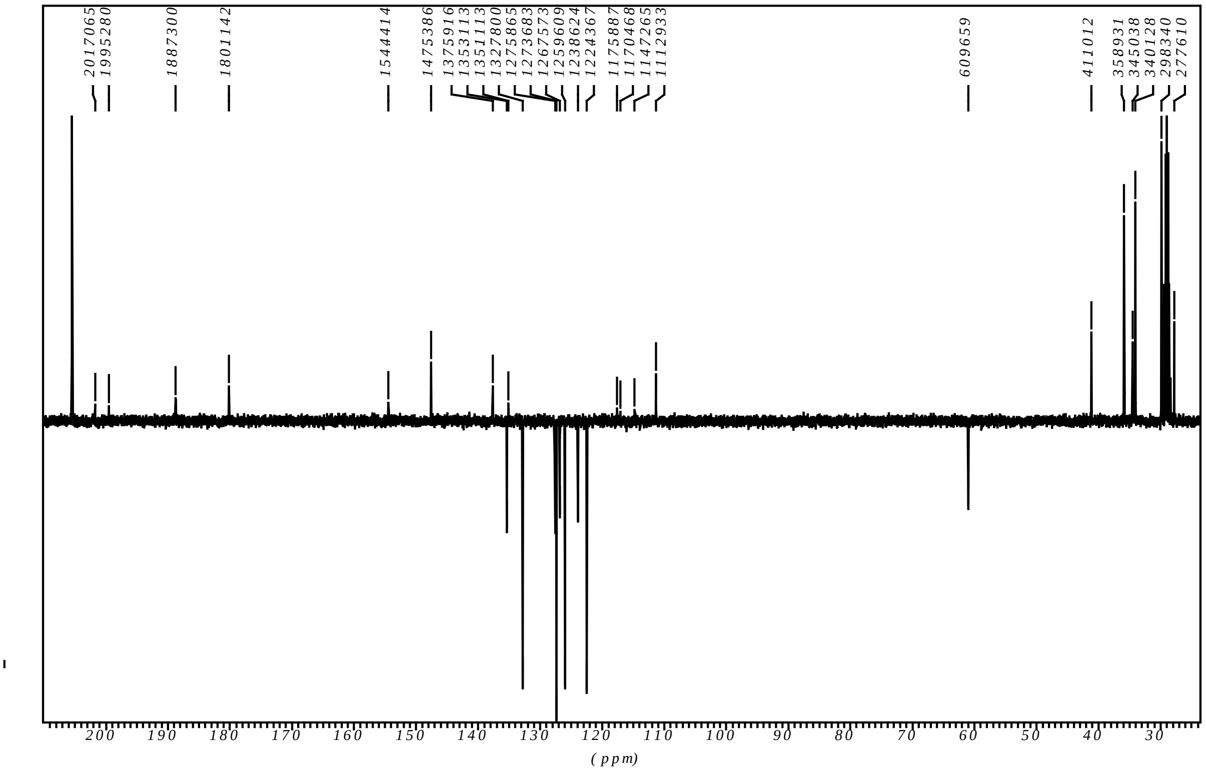

Figure S-44. ^{13}C NMR spectrum of 2-[3-(*N*-trifluroacetylamino)propionyl]-1-indanone **3u**.

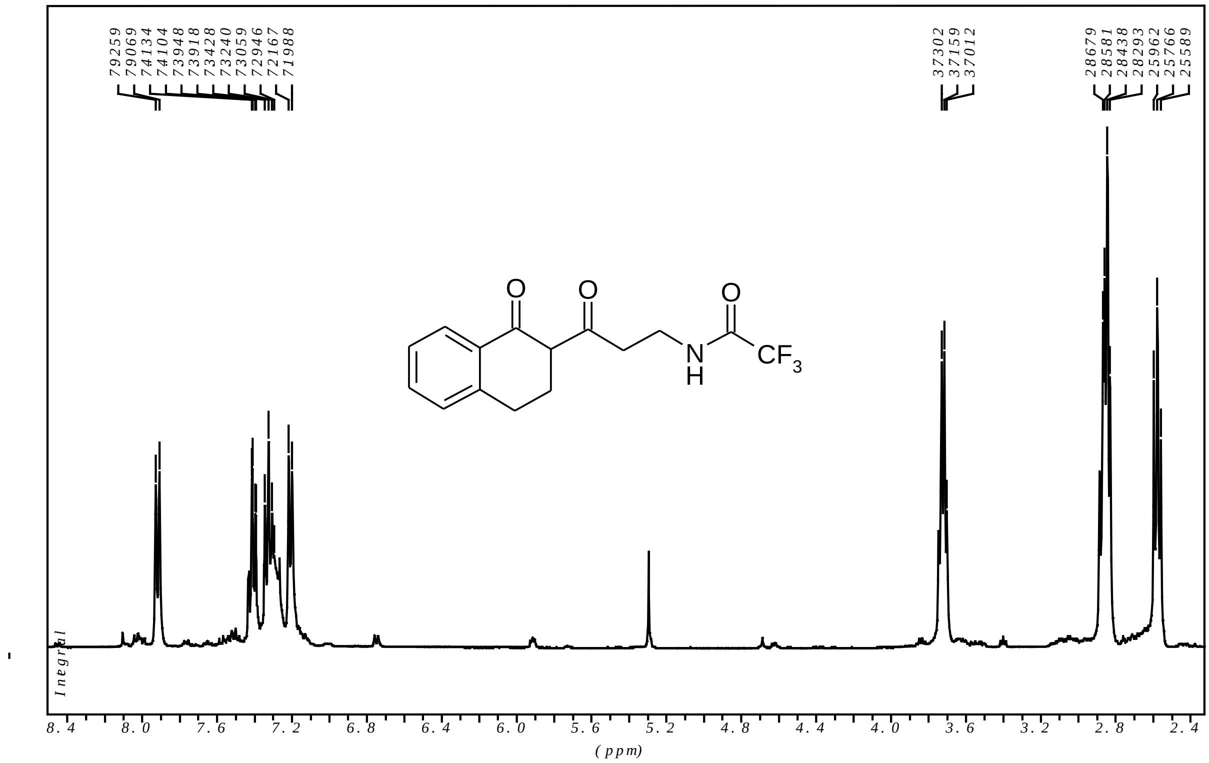

Figure S-45. ^{1}H NMR spectrum of 2-[3-(*N*-trifluoroacetylamino)propionyl]-1-tetralone **3v**.

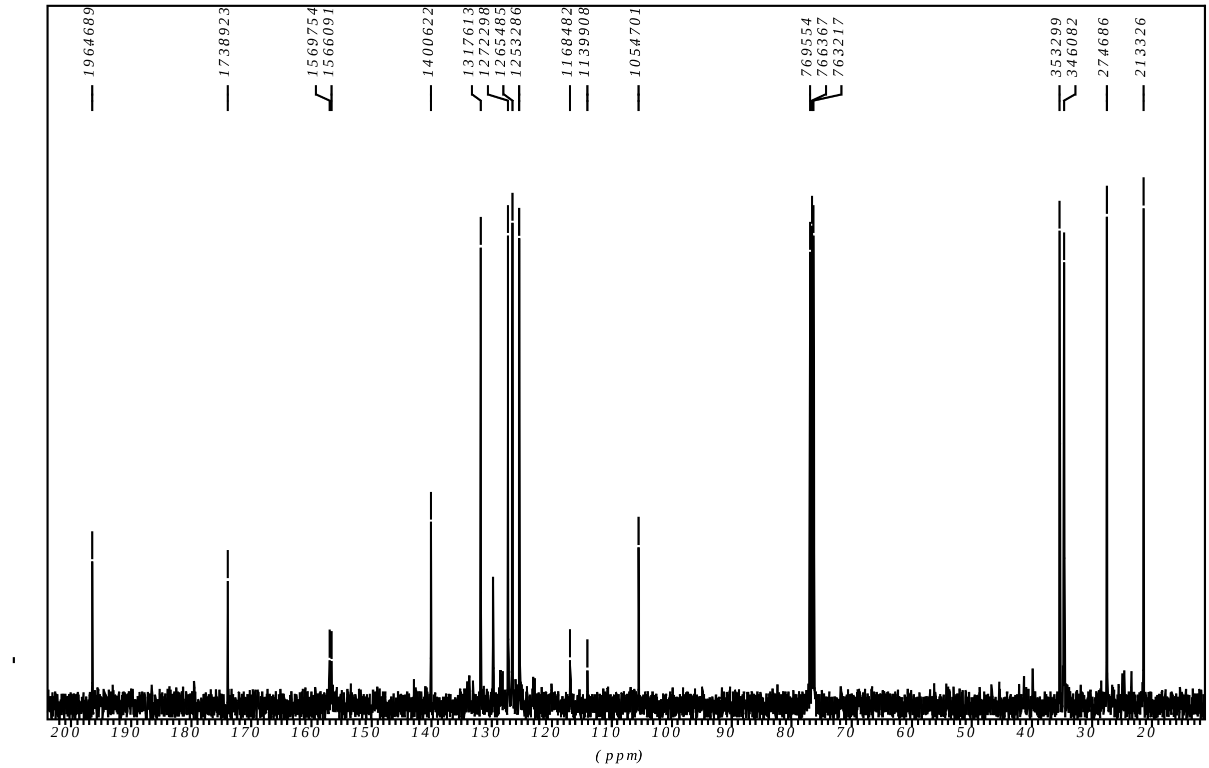

Figure S-46. ^{13}C NMR spectrum of 2-[3-(*N*-trifluoroacetylamino)propionyl]-1-tetralone **3v**.

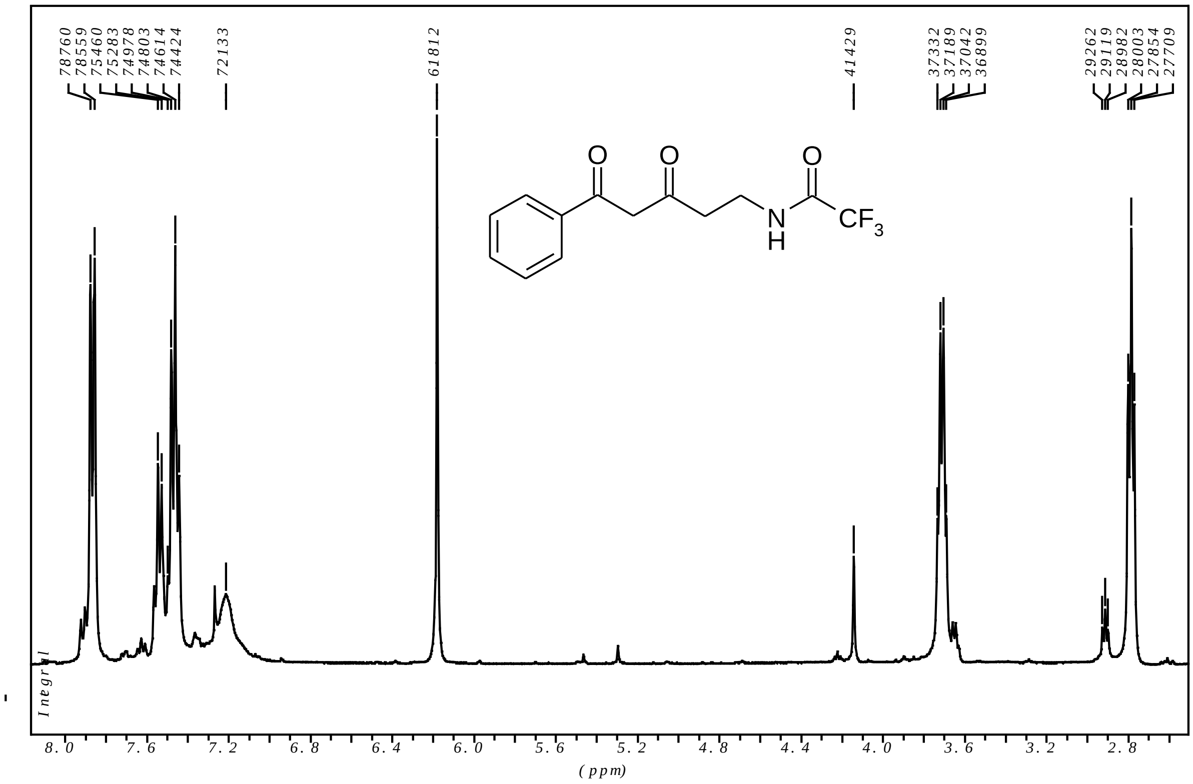

Figure S-47. ^{1}H NMR spectrum of 5-(*N*-trifluoroacetylamino)-1-phenylpentan-1,3-dione **3w**.

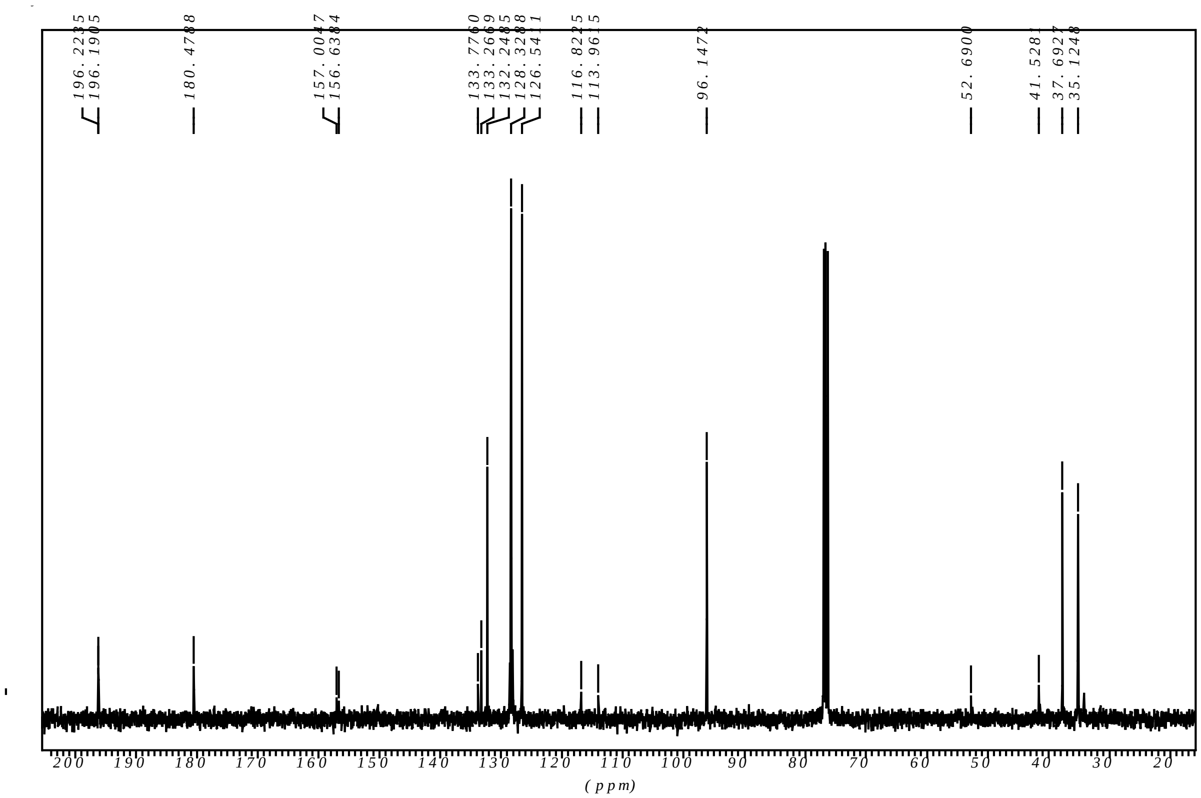

Figure S-48. ^{13}C NMR spectrum of 5-(*N*-trifluoroacetylamino)-1-phenylpentan-1,3-dione **3w.**

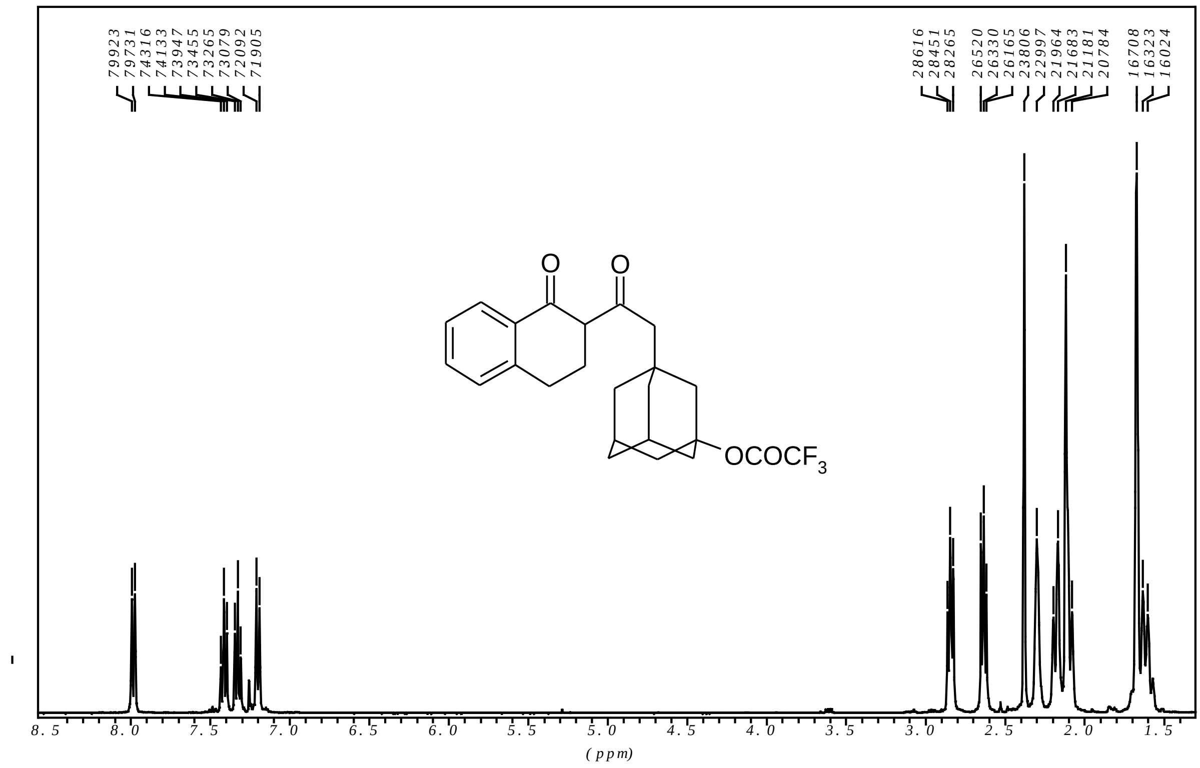

Figure S-49. ^{1}H NMR spectrum of 2-[2-(3-trifluoroacetoxy-1-adamantyl)acetyl]-1-tetralone **3x**.

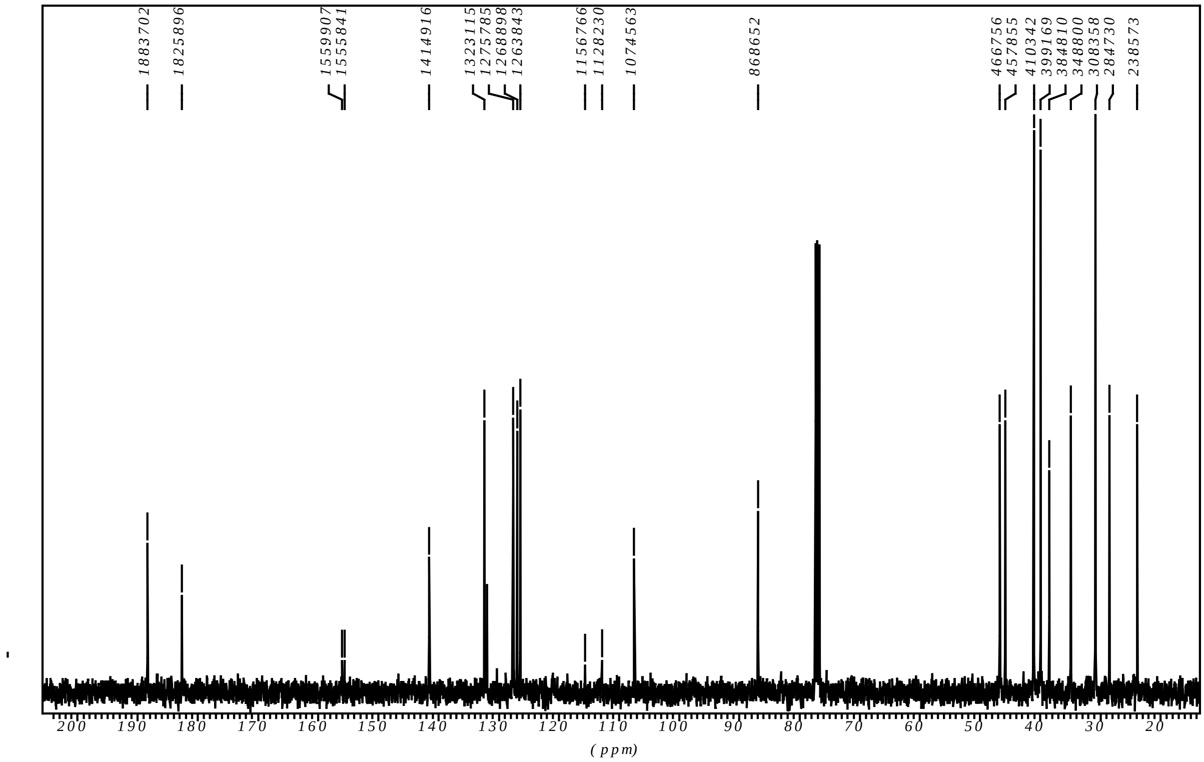

Figure S-50. ^{13}C NMR spectrum of 2-[2-(3-trifluoroacetoxy-1-adamantyl)acetyl]-1-tetralone **3x**.

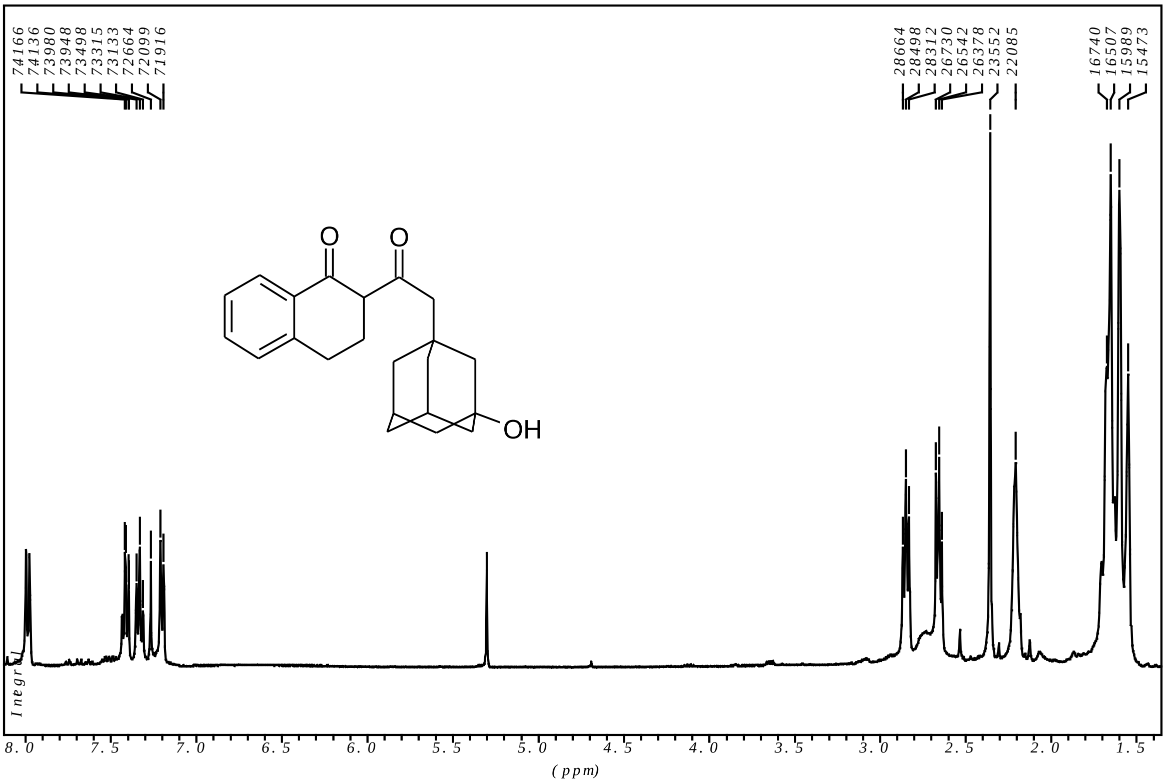

Figure S-51. ^{1}H NMR spectrum of 2-[2-(3-hydroxy-1-adamantyl)acetyl]-1-tetralone **3y**.

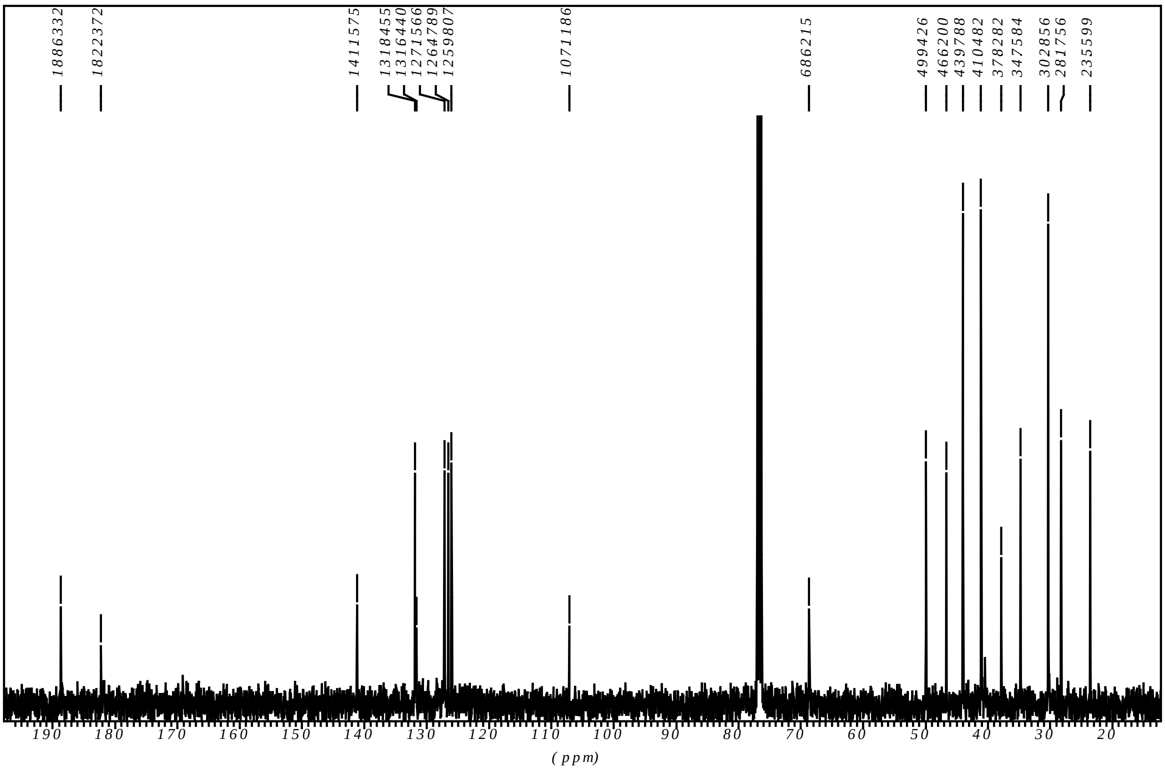

Figure S-52. ^{13}C NMR spectrum of 2-[2-(3-hydroxy-1-adamantyl)acetyl]-1-tetralone **3y**.

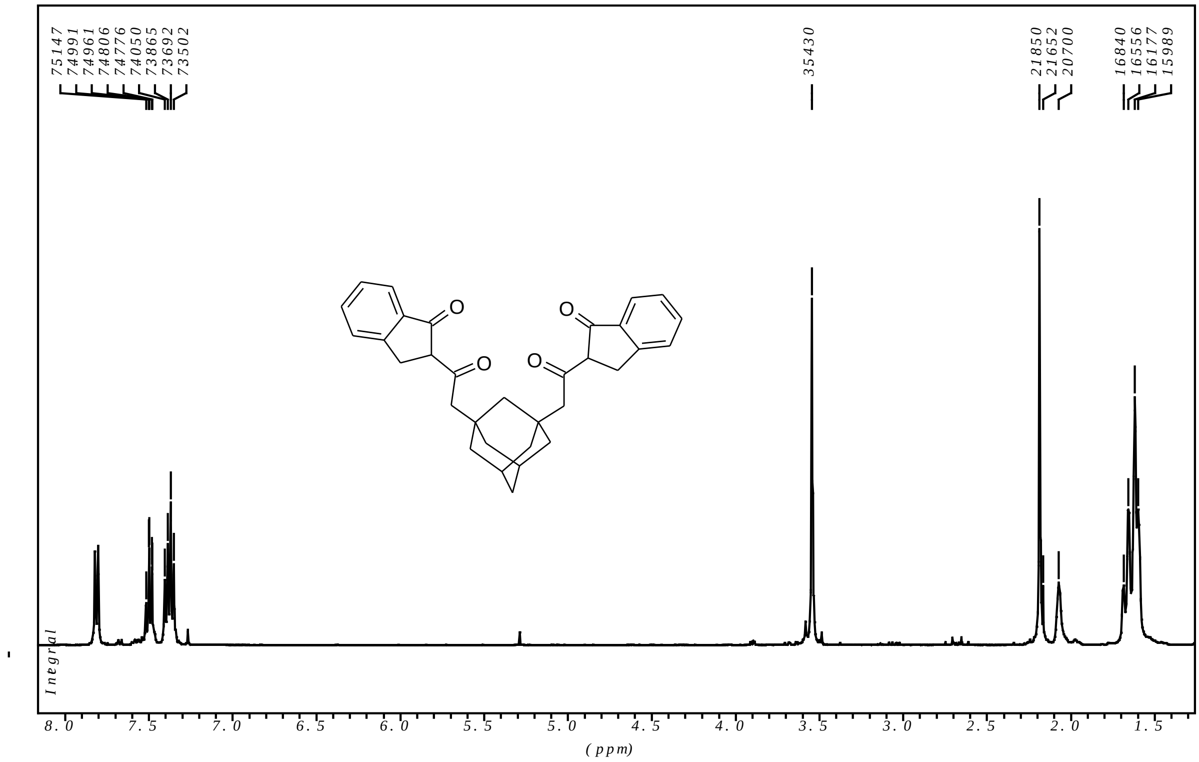

Figure S-53. ^{1}H NMR spectrum of 1,3-di(indanone-1-yl-2)adamantane **3z**.

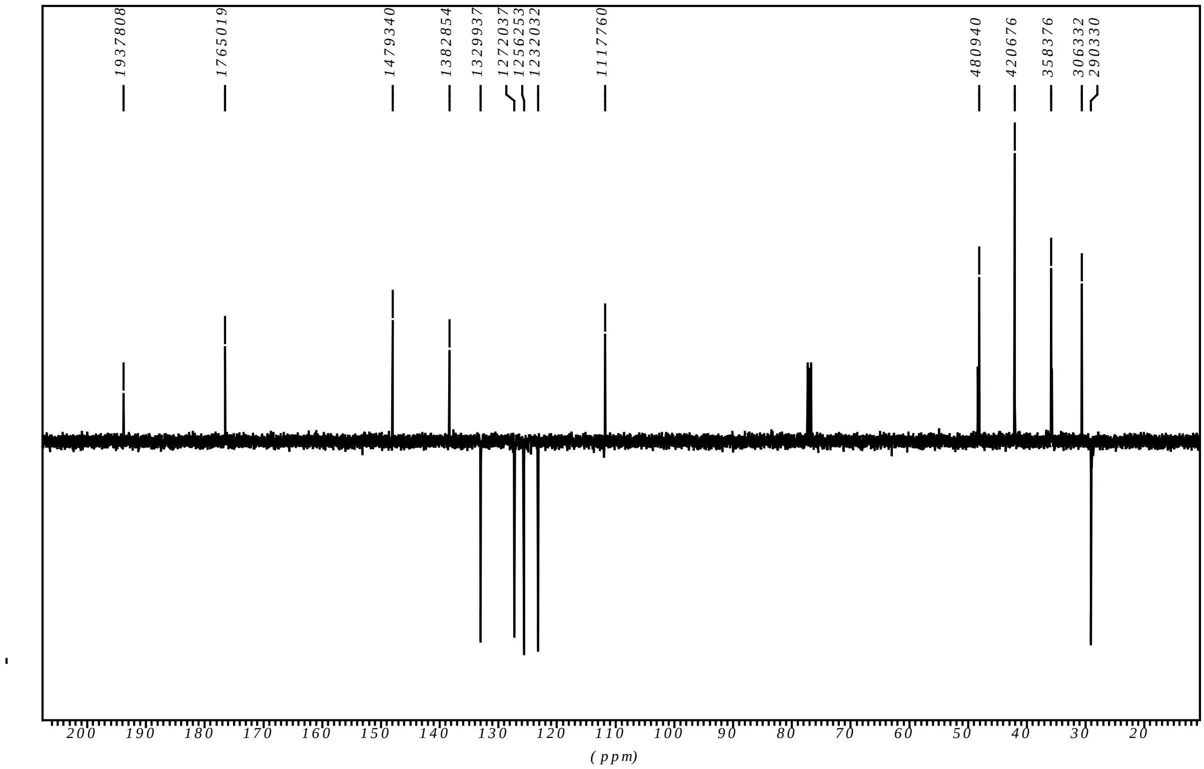

Figure S-54. ^{13}C NMR spectrum of 1,3-di(indanone-1-yl-2)adamantine **3z.**

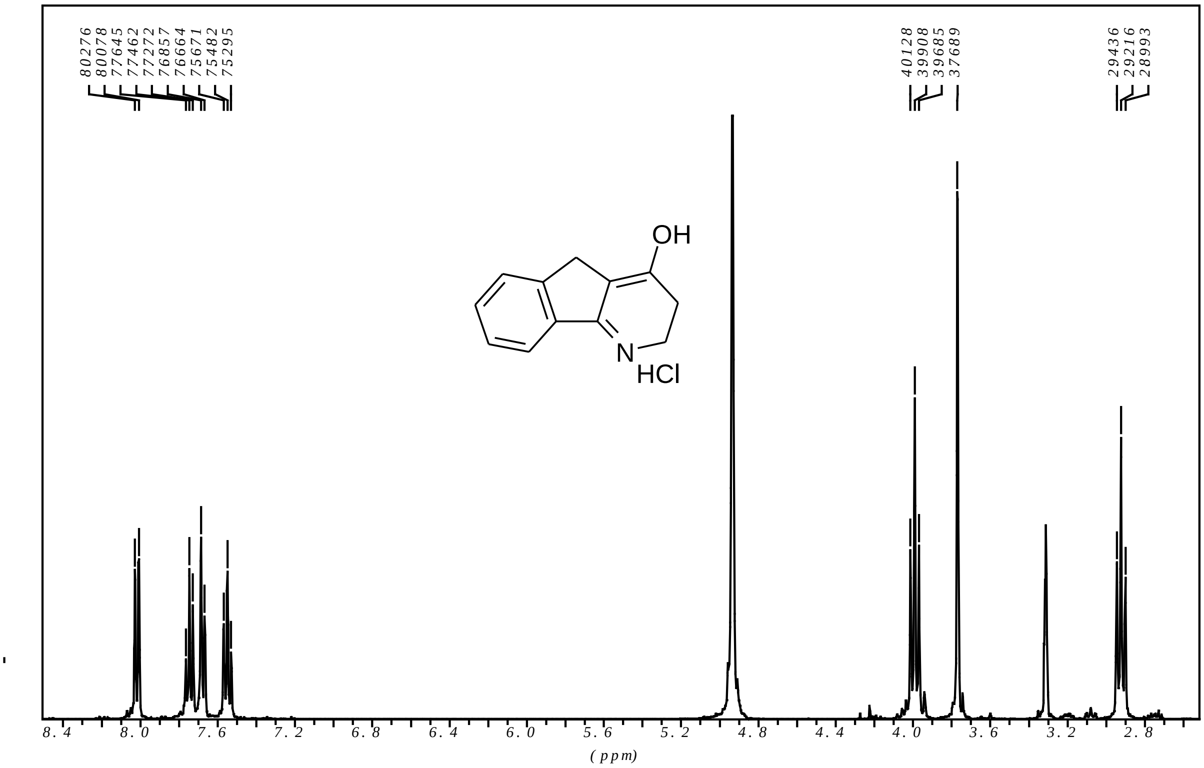

Figure S-55. ^{1}H NMR spectrum of 1-hydroxy-4-aza-2,3-dihydrofluorene hydrochloride **4a**.

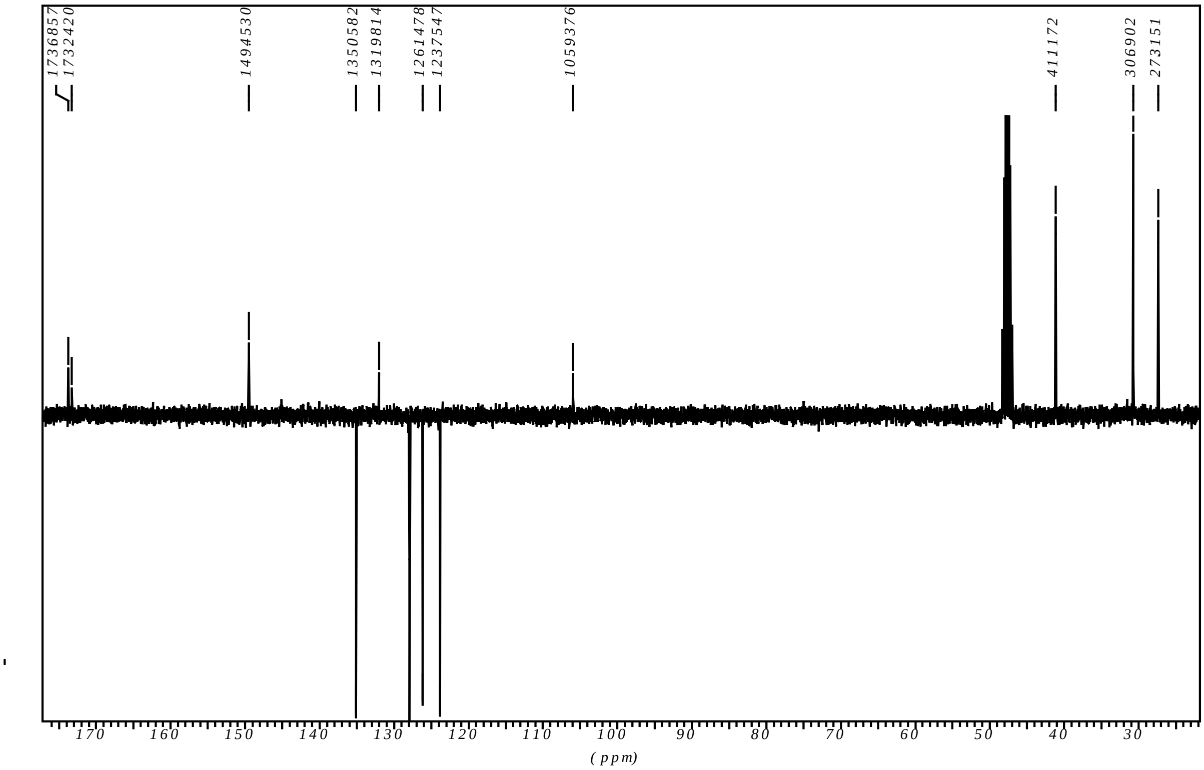

Figure S-56. ^{13}C NMR spectrum of 1-hydroxy-4-aza-2,3-dihydrofluorene hydrochloride **4a**.

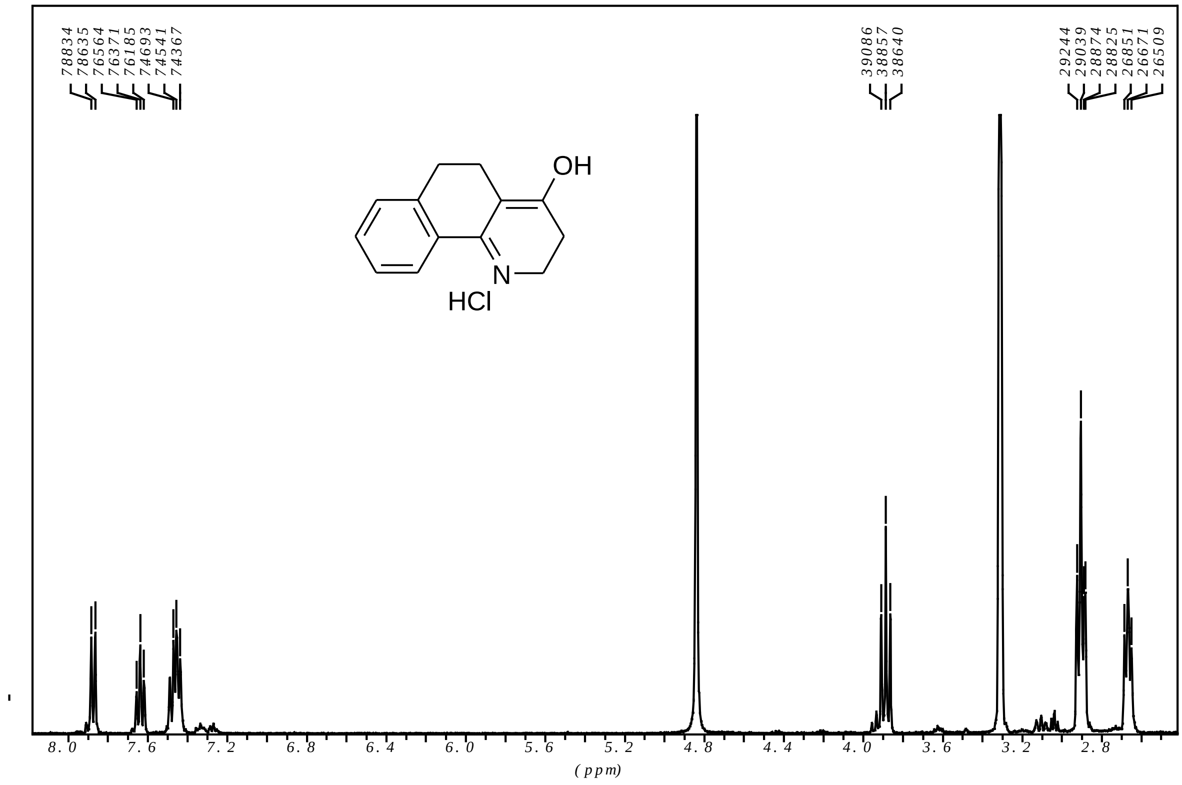

Figure S-57. ^{1}H NMR spectrum of 1-hydroxy-4-aza-2,3,9,10-tetrahydrophenanthrene hydrochloride **4b**.

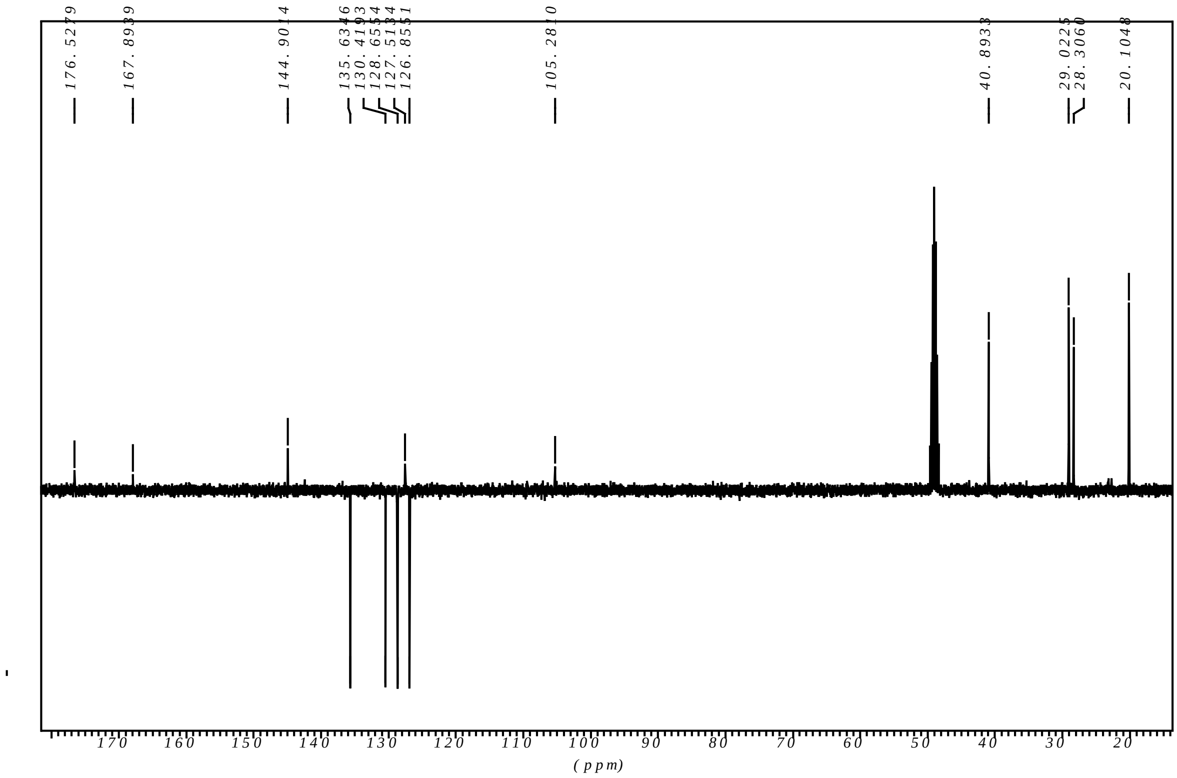

Figure S-58. ^{13}C NMR spectrum of 1-hydroxy-4-aza-2,3,9,10-tetrahydrophenanthrene hydrochloride **4b**.

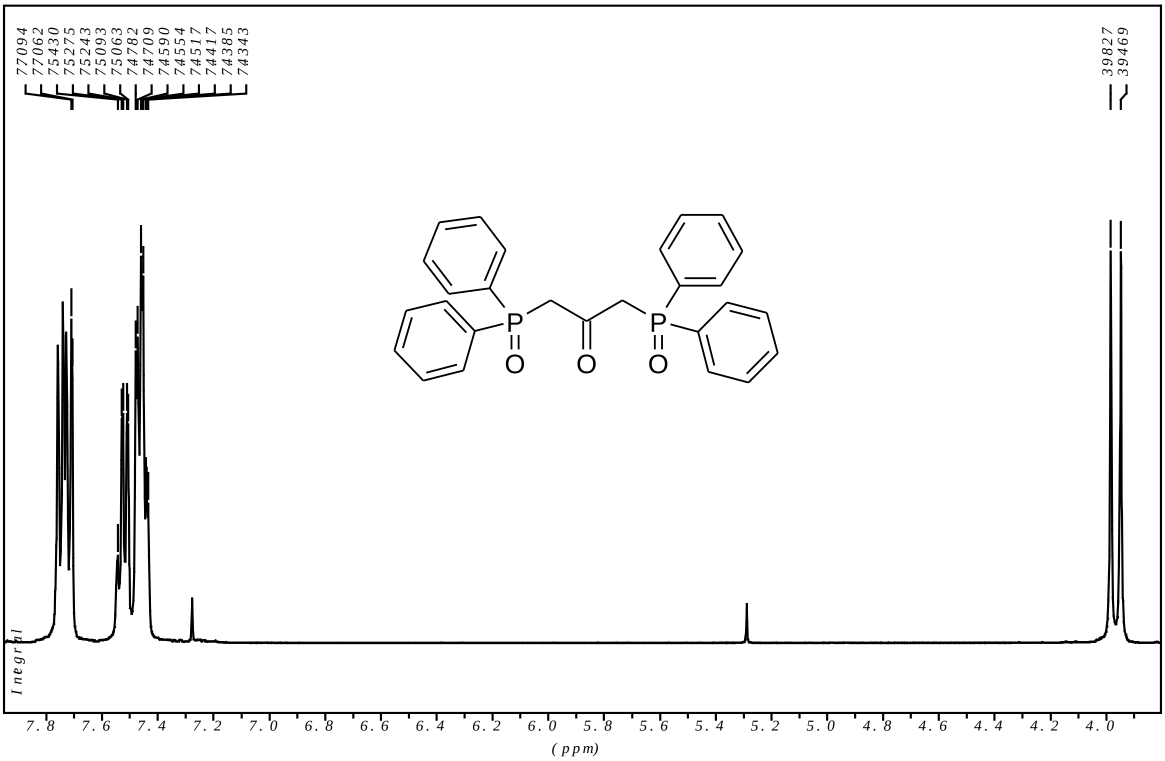

Figure S-59. ^{1}H NMR spectrum of 1,3-di-(diphenylphosphoryl)acetone **5**.

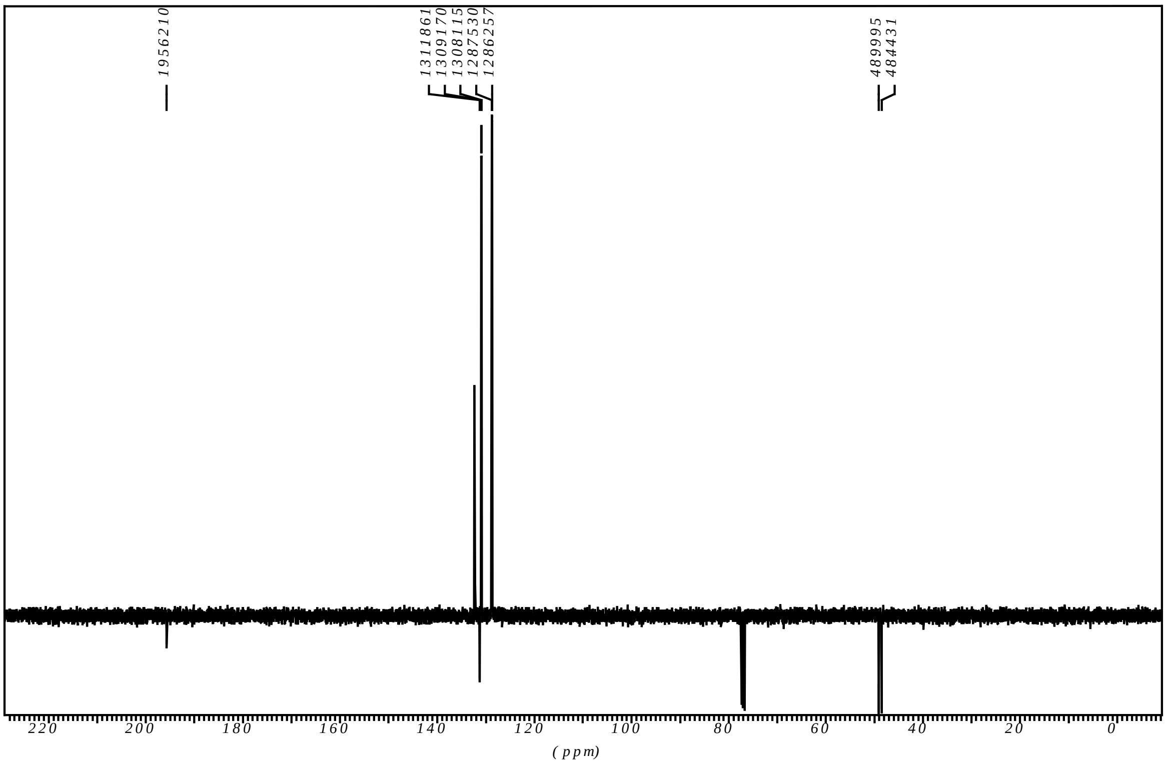

Figure S-60. ^{13}C NMR spectrum of 1,3-di-(diphenylphosphoryl)acetone **5**.

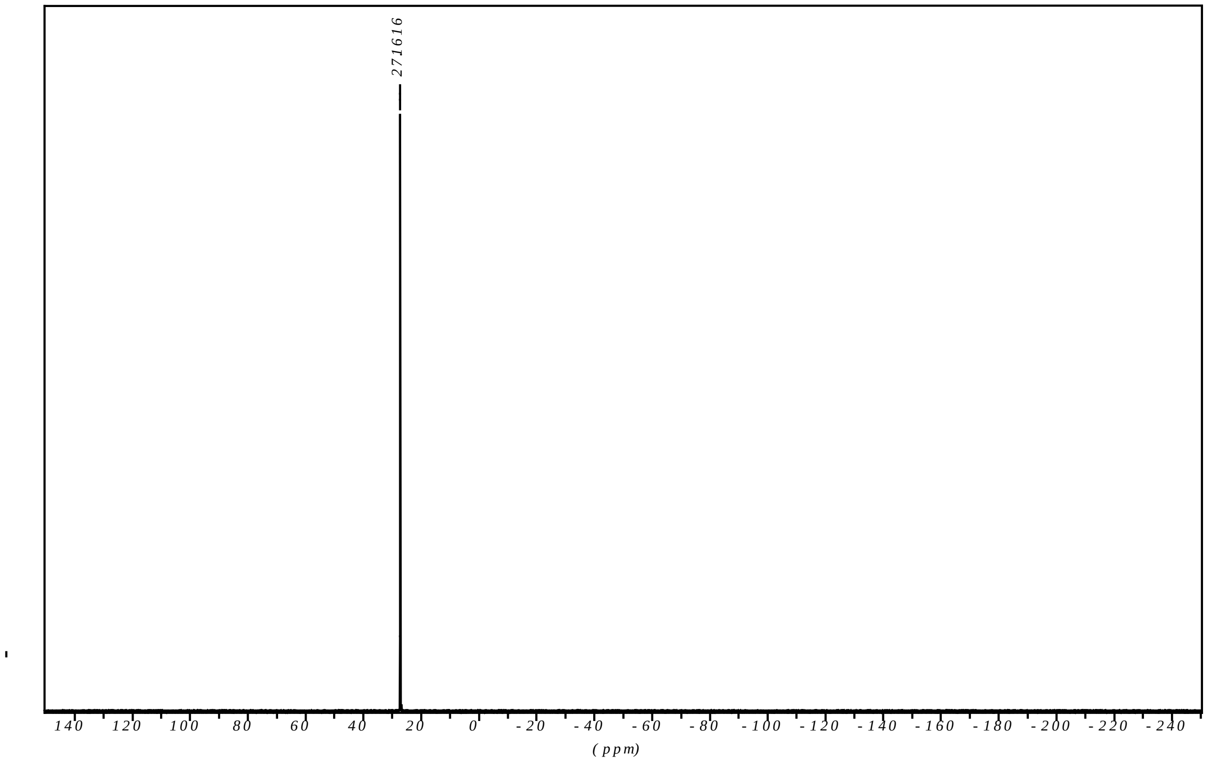

Figure S-61. ^{31}P NMR spectrum of 1,3-di-(diphenylphosphoryl)acetone **5**.

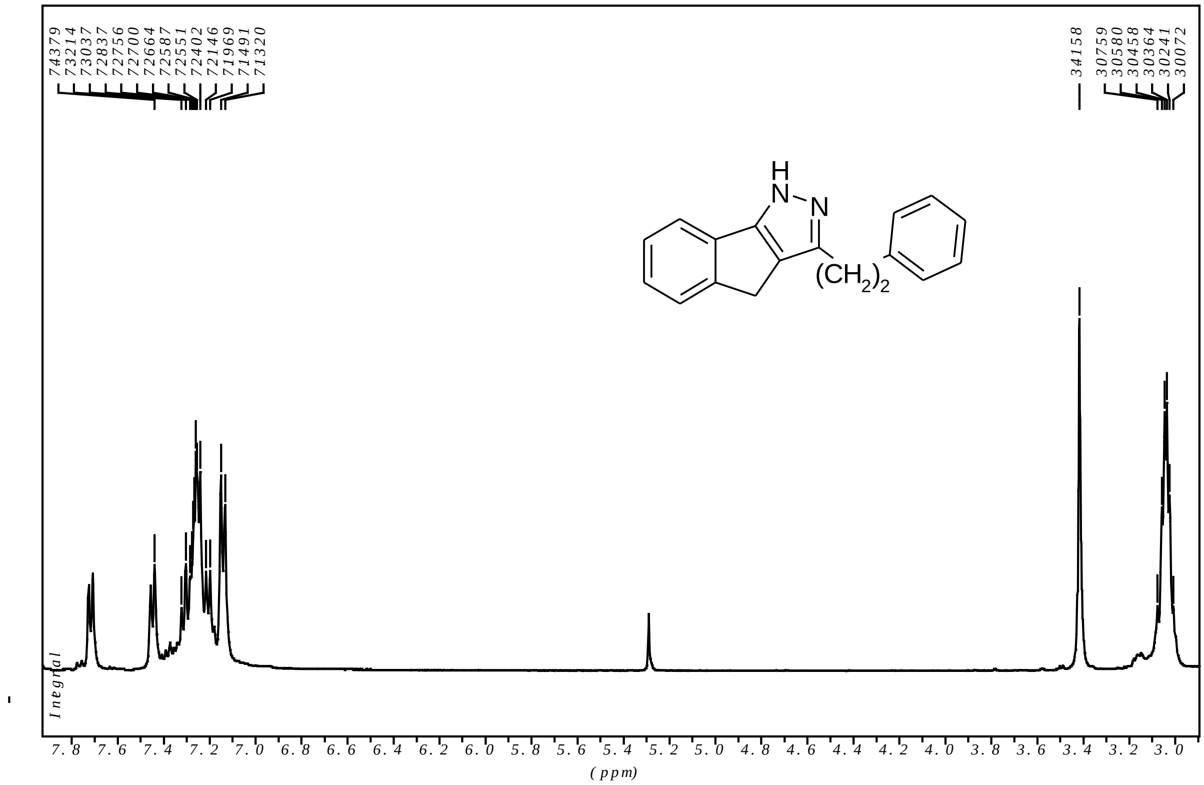

Figure S-62. ^{1}H NMR spectrum of 3-phenethyl-1,4-dihydroindeno[1,2-*c*]pyrazole **6a**.

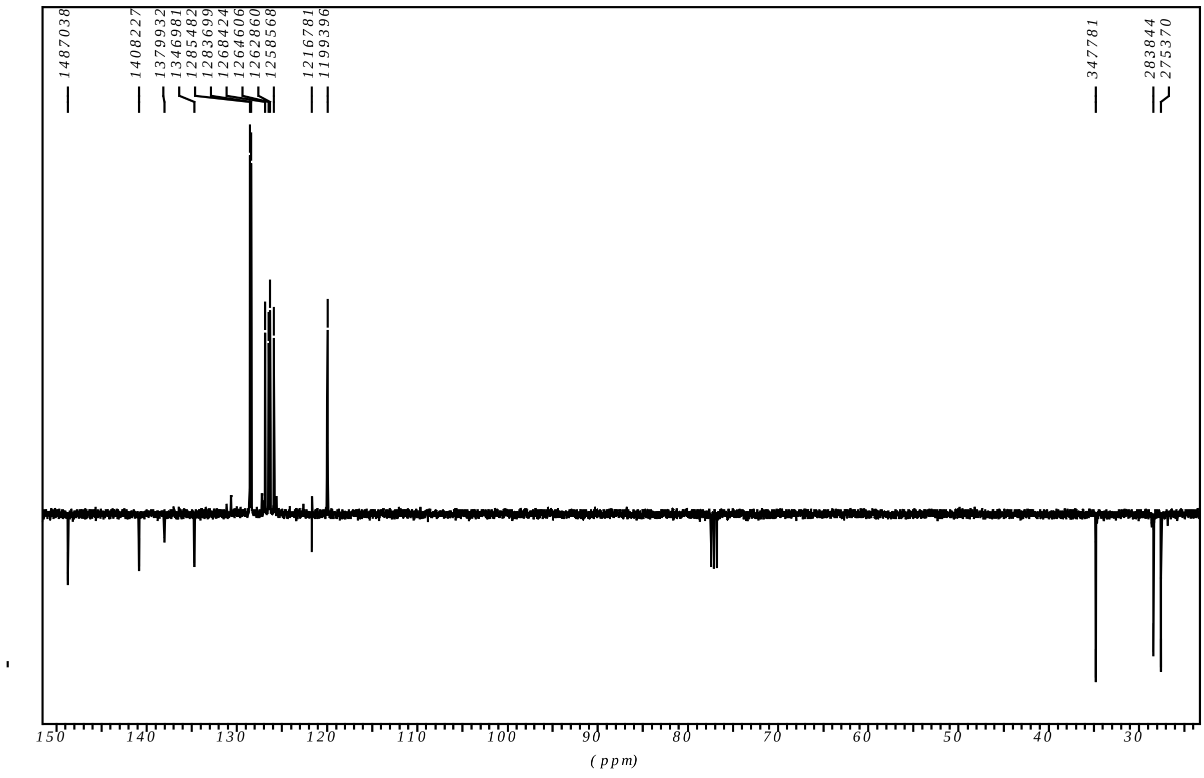

Figure S-63. ^{13}C NMR spectrum of 3-phenethyl-1,4-dihydroindeno[1,2-*c*]pyrazole **6a**.

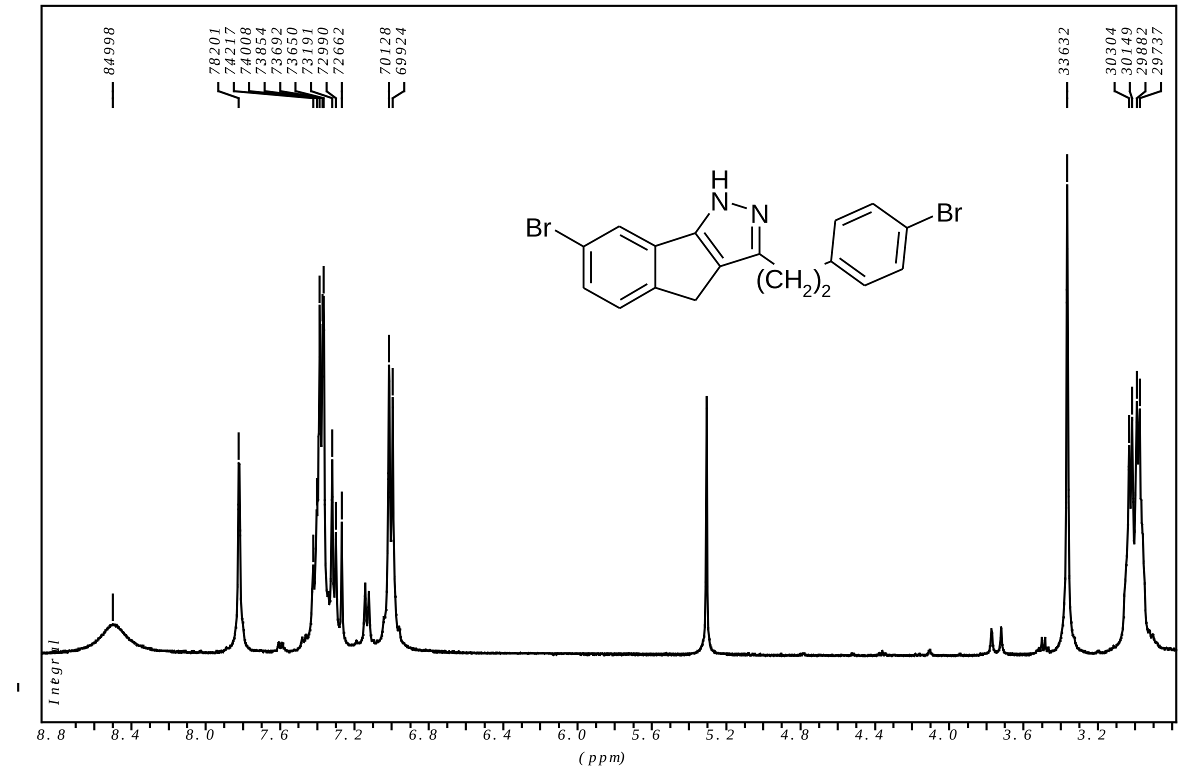

Figure S-64. ^{1}H NMR spectrum of 7-bromo-3-[2-(4-bromophenyl)ethyl]-1,4-dihydroindeno[1,2-*c*]pyrazole **6b**.

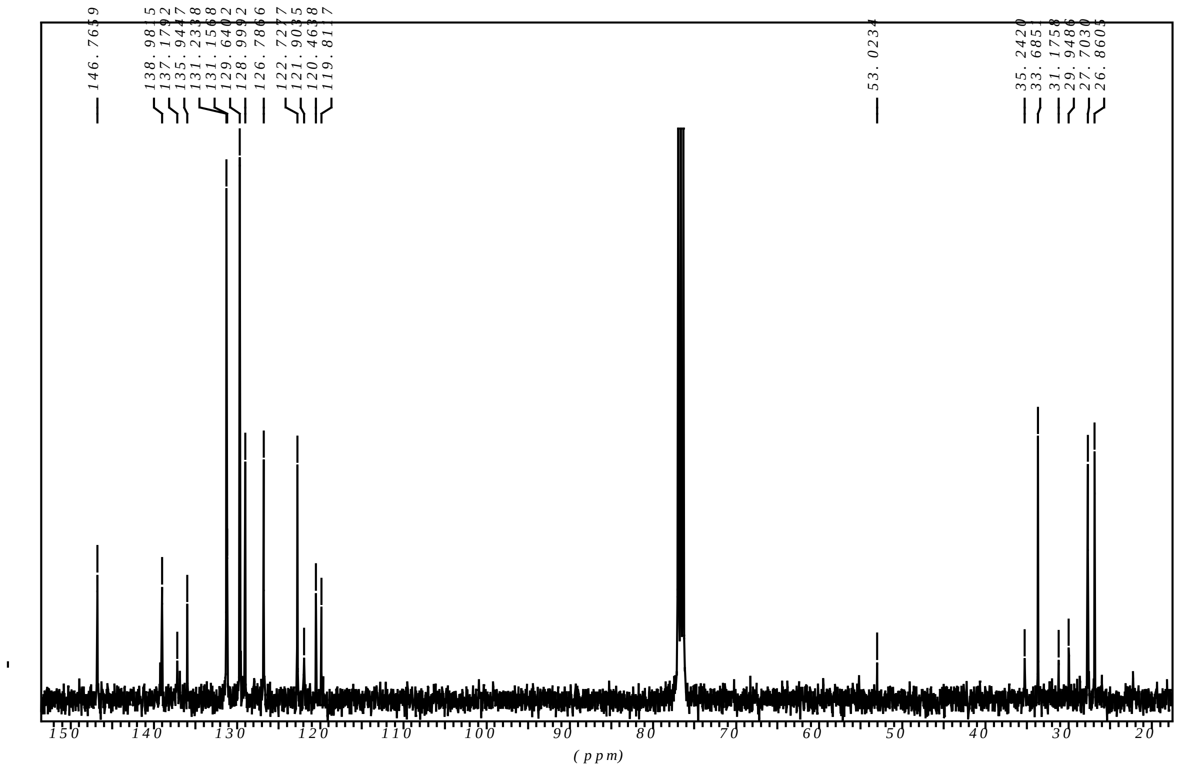

Figure S-65. ^{13}C NMR spectrum of 6-bromo-3-[2-(4-bromophenyl)ethyl]-1,4-dihydroindeno[1,2-*c*]pyrazole **6b**.

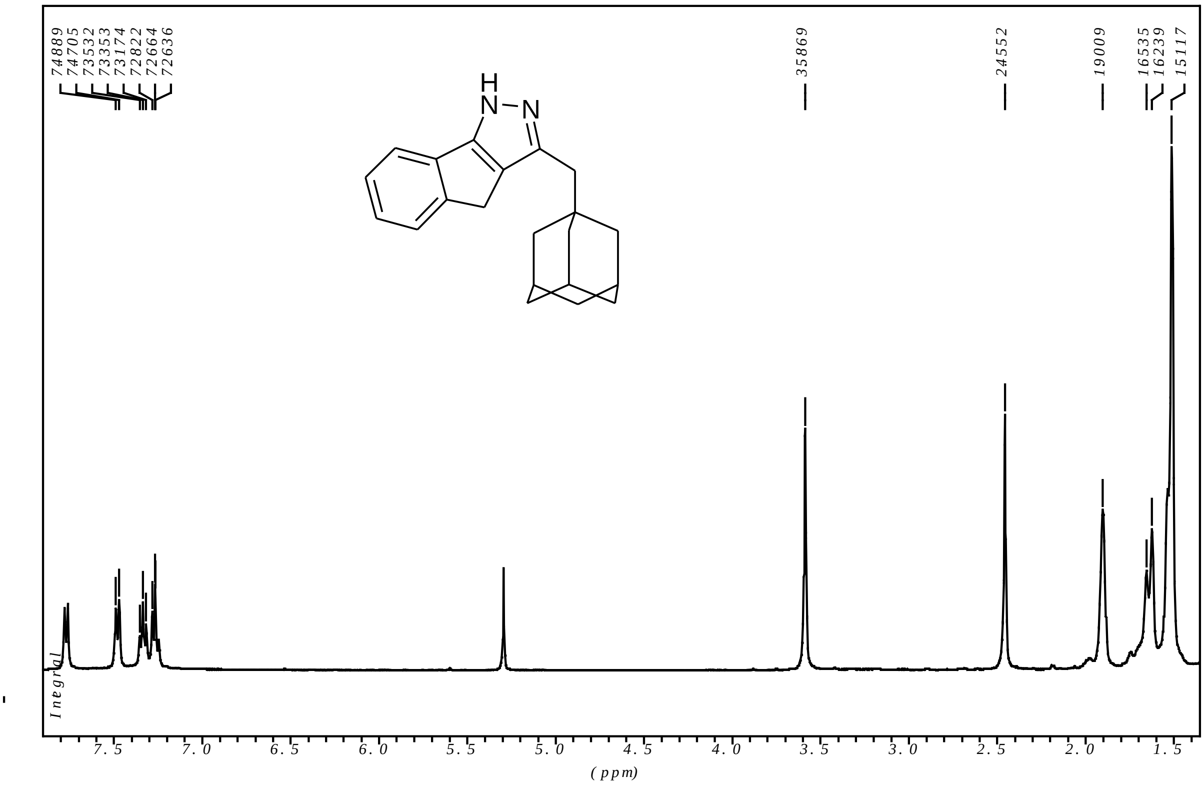

Figure S-66. ^{1}H NMR spectrum of 3-(1-adamantylmethyl)-1,4-dihydroindeno[1,2-*c*]pyrazole **6c**.

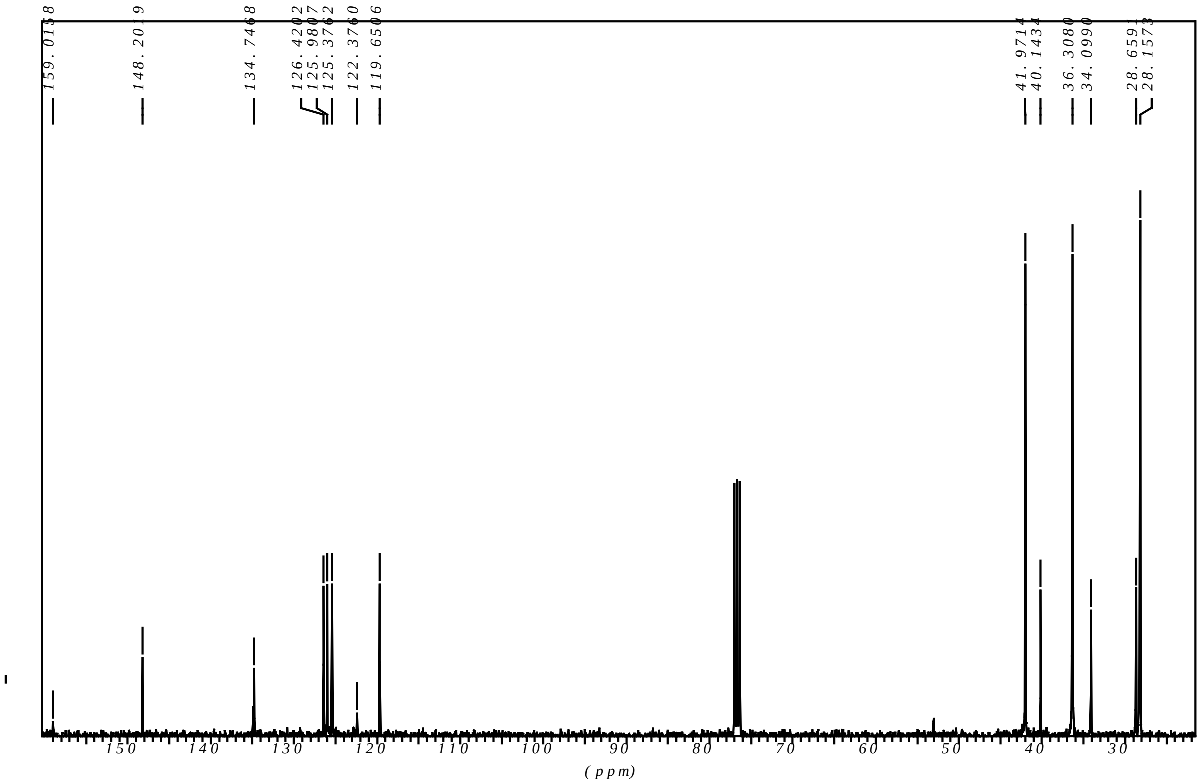

Figure S-67. ^{13}C NMR spectrum of 3-(1-adamantylmethyl)-1,4-dihydroindeno[1,2-*c*]pyrazole 6c.

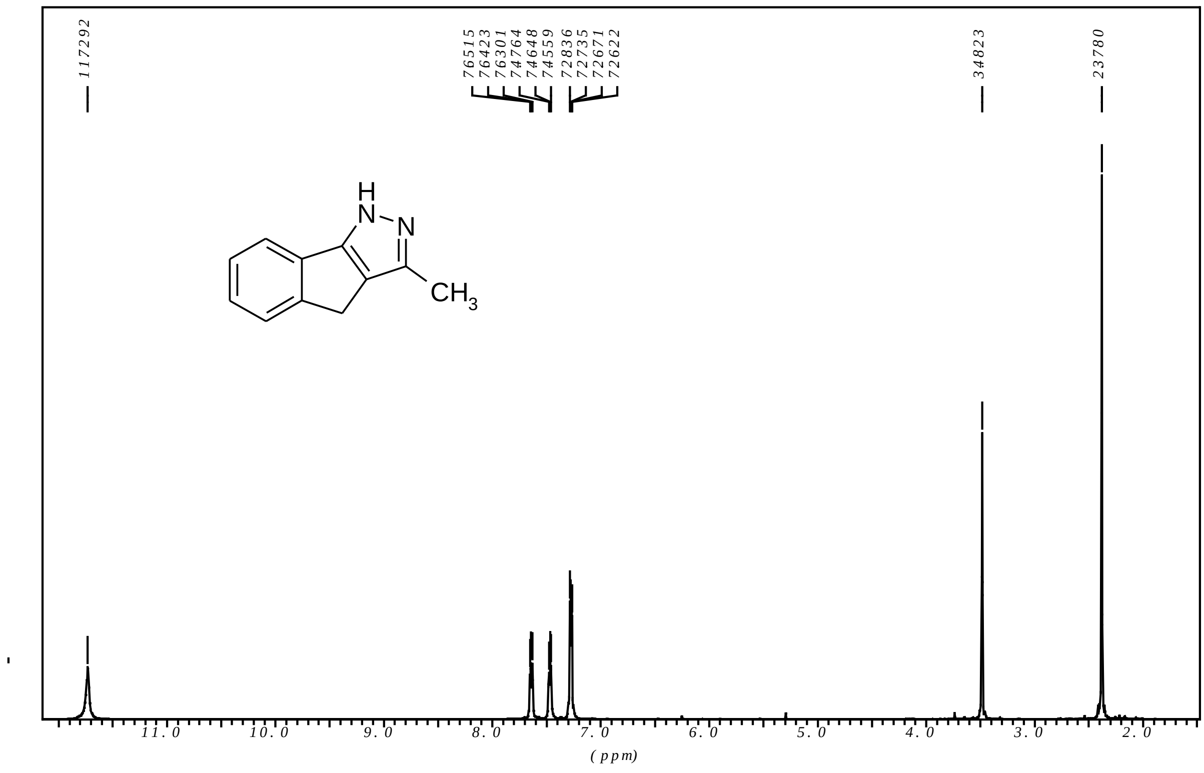

Figure S-68. ^{1}H NMR spectrum of 3-methyl-1,4-dihydroindeno[1,2-*c*]pyrazole **6d**.

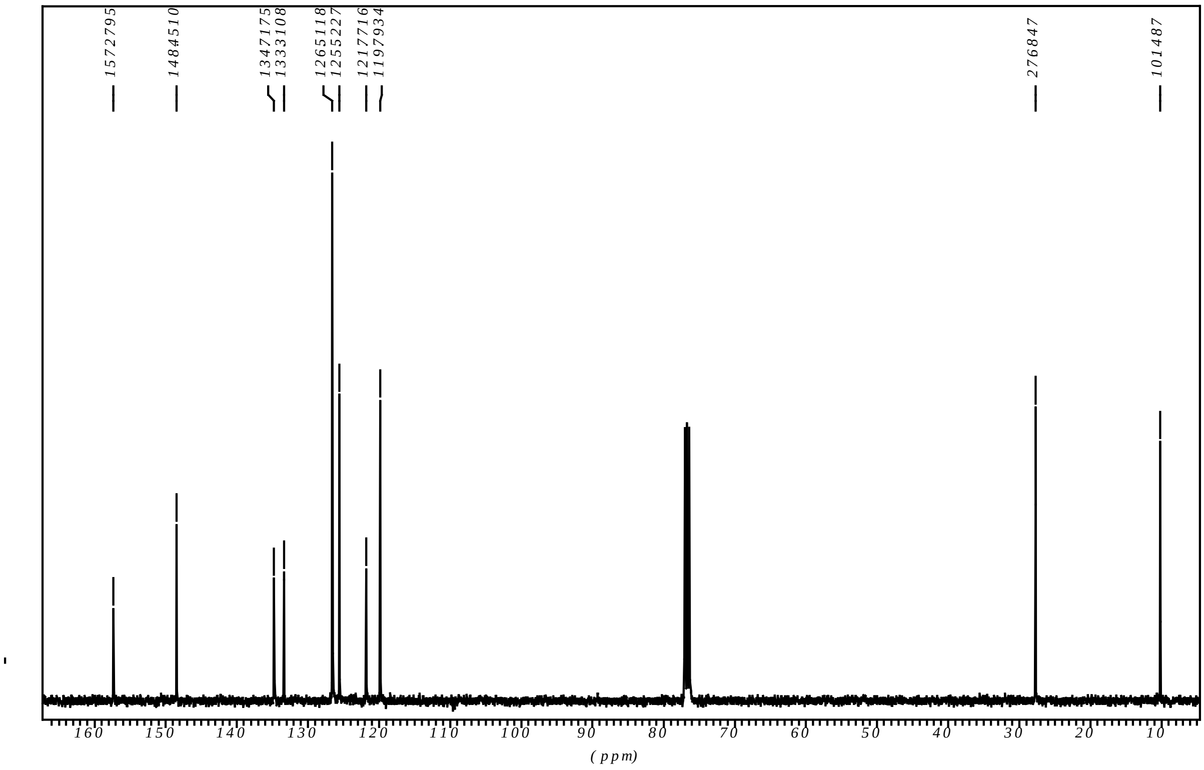

Figure S-69. ^{13}C NMR spectrum of 3-methyl-1,4-dihydroindeno[1,2-*c*]pyrazole **6d**.
